# Research on Moringa (*Moringa oleifera* Lam.) in Africa

**DOI:** 10.3390/plants13121613

**Published:** 2024-06-11

**Authors:** Hamid El Bilali, Iro Dan Guimbo, Romaric Kiswendsida Nanema, Hamidou Falalou, Zakaria Kiebre, Veli-Matti Rokka, Sheirita Reine Fanta Tietiambou, Jacques Nanema, Lawali Dambo, Francesca Grazioli, Abdel Kader Naino Jika, Maria Gonnella, Filippo Acasto

**Affiliations:** 1 International Centre for Advanced Mediterranean Agronomic Studies (CIHEAM-Bari), Via Ceglie 9, Valenzano, 70010 Bari, Italy; 2 Department of Rural Engineering, Water and Forests, Faculty of Agronomy, Abdou Moumouni University, Niamey P.O. Box 237, Niger; danguimbo@yahoo.fr; 3 Department of Plant Biology and Physiology, Joseph Ki-Zerbo University, PB 7021, Ouagadougou 03, Burkina Faso; romaric.nanema@ujkz.bf (R.K.N.); zakaria.kiebre@ujkz.bf (Z.K.); 4 International Crops Research Institute for the Semi-Arid Tropics (ICRISAT), Niamey BP 12404, Niger; falalou.hamidou@icrisat.org; 5 Natural Resources Institute Finland (Luke), Myllytie 1, 31600 Jokioinen, Finland; veli-matti.rokka@luke.fi; 6 University Centre of Gaoua, Nazi BONI University, 01 BP, Bobo-Dioulasso 1091, Burkina Faso; tietiambou.fanta@gmail.com; 7 Programme Agrinovia, Joseph Ki-Zerbo University, 03 BP, Ouagadougou 7021, Burkina Faso; jacquesnanema@yahoo.fr; 8 Department of Geography, Faculty of Letters and Human Sciences, Abdou Moumouni University, Niamey P.O. Box 237, Niger; lawali.dambo@gmail.com; 9 Alliance Bioversity International—CIAT (Centro Internacional de Agricultura Tropical), Via San Domenico 1, 00153 Rome, Italy; 10 Department of Crop Production, Faculty of Agronomy, Abdou Moumouni University, Niamey P.O. Box 237, Niger; kaderjika@gmail.com; 11 Institute of Sciences of Food Production, National Research Council of Italy (CNR), Via G. Amendola 122/O, 70126 Bari, Italy; maria.gonnella@ispa.cnr.it; 12 Italian Agency for Development Cooperation (AICS), Ouaga 2000—Secteur 54, Arrondissement n. 12, Ouagadougou 01, Burkina Faso; filippo.acasto@aics.gov.it

**Keywords:** neglected and underutilized species, NUS, orphan crops, *M. oleifera*, drumstick tree, horseradish tree, bibliometric, food security, livelihoods, climate change, SUSTLIVES

## Abstract

While *Moringa oleifera* Lam. is gaining importance in Africa, especially sub-Saharan Africa, it is unclear whether research is following the quick pace of its development on the continent. Therefore, this article analyzes the landscape of research dealing with moringa in Africa. This systematic review draws upon 299 eligible articles identified through a search carried out on the Web of Science in April 2023. Research on *M. oleifera* is rather recent in Africa but interest is increasing among scholars. While the research field is multidisciplinary and cross-sectoral, the literature seems to focus on biological and environmental sciences. Moreover, research is performed mainly in South Africa, Nigeria, Egypt, and Ghana. The analysis suggests a significant potential contribution of moringa to food security and nutrition, climate change mitigation/adaptation, farming systems resilience, and livelihoods. Its versatility and diverse applications and uses make moringa particularly interesting for developing countries, such as African ones. However, this review also underscores some factors hindering its development. Therefore, there is a need to strengthen research on moringa to unlock its potential in Africa. Investments in research, innovation, and development can help address the many challenges that Africa faces and contribute to the transition towards sustainable and resilient food systems.

## 1. Introduction

Agriculture still plays an important socio-economic role in Africa. Indeed, the most recent data from the World Bank [1] show that the contribution of the primary sector (viz. agriculture, forestry, and fishing) to the gross domestic product (GDP) in African countries ranges from 1.6% in Libya and 1.8% in Botswana and Djibouti to 60.4% in Sierra Leone and 62.7% in Somalia. Data regarding the share of employment in agriculture [2] show that it ranges from 1% in Djibouti to 70% in Mozambique, 71% in Niger, 73% in Burkina Faso, 74% in Madagascar, and even 86% in Burundi (Appendix A). Food insecurity and malnutrition present significant challenges throughout the African continent [3]. The overall prevalence of undernourishment in the population remained high in the period 2020–2022, with figures ranging from 3.0% in Tunisia, 4.3% in Seychelles, and 4.9% in Ghana to 46.0% in Lesotho, 48.7% in Somalia, 48.7% in the Central African Republic (CAR), and 51.0% in Madagascar. The situation becomes even more concerning when considering the prevalence of moderate or severe food insecurity, with figures ranging from 14.7% in Seychelles and 20.3% in South Africa to 81.3% in CAR, 82.4% in Malawi, 87.3% in South Sudan, 88.2% in Congo, and 89.2% in Sierra Leone during the same period (Appendix A). Moreover, evidence suggests that Sub-Saharan Africa [4,5,6,7] will experience the most significant impacts of climate change. Agriculture, which relies primarily on rainfall, is highly susceptible to climate variability [8,9]. The above-mentioned challenges highlight the urgent need for the transition towards sustainable and resilient agri-food systems in Africa [10].

Neglected and underutilized species (NUS) are widely recognized as valuable assets in promoting sustainable development and transitioning to sustainable and resilient agri-food systems [11]. Generally, crops can be classified into two categories: major crops and underutilized crops, which are also known as orphan, neglected, minor, promising, or local crops [12]. These terms are used interchangeably to refer to crops that have received little attention from researchers, plant breeders, and policymakers [13,14]. Of the 300,000 to 500,000 plant species known worldwide, 30,000 are edible [15]. Although the number of edible plants varies, some scholars consider 10,000 as a realistic estimate [16,17]. Over 7000 crop species have been cultivated, domesticated, or collected from the wild throughout human history [15,18]. However, only about 150 species are commercially cultivated, and 103 of them provide up to 90% of dietary calories [19,20]. Accordingly, tens of thousands of edible plant species are underutilized [21].

The promotion of neglected and underutilized crop species has been reported to contribute significantly to agrobiodiversity conservation [14,22], food and nutrition security [12,14,23,24,25], climate change adaptation and mitigation [23], environmental integrity and health [23], human health [23], and rural livelihood sustainability and resilience [14]. In this regard, Mabhaudhi et al. [23] stated that “*Owing to reports of their potential under water scarcity, there is an argument to promote them to sustainably address challenges such as increasing drought and water scarcity, food and nutrition insecurity, environmental degradation, and employment creation under climate change*” (p. 695). Mabhaudhi et al. [23] argued that research, enabling policies and strategies, and investments are needed to promote NUS as an essential development tool in the Global South. Mabhaudhi et al. [26] assume that the promotion of NUS could also contribute to the achievement of the Sustainable Development Goals (SDGs), specifically SDGs 1 (No poverty), 2 (Zero hunger), 3 (Good health and well-being), and 15 (Life on land). Therefore, it is essential to conduct research to unlock the potential of NUS [27], especially in developing countries [21].

One such NUS is *Moringa oleifera* Lam. *M. oleifera* is a fast-growing, deciduous tree of the family of Moringaceae, native to the Indian subcontinent [28,29]. Common names include moringa, drumstick tree, horseradish tree, and ben oil tree or benzolive tree [29,30]. Synonyms include *Guilandina moringa* L., *Hyperanthera moringa* (L.) Vahl, and *Moringa pterygosperma* Gaertn. nom. illeg. [31]. The species was first described in 1785 by the French naturalist Jean-Baptiste Lamarck [32]. *M. oleifera* is grown mainly in semiarid, tropical, and subtropical areas [33]. It is particularly suitable for dry regions as it can be rainfed, without the need for irrigation [33]. Moringa is mainly grown in South and Southeast Asia, and India is the largest producer [33]. However, besides South and Southeast Asia, the species is nowadays grown in different world regions, including Central America, the Caribbean, South America, Africa, and Oceania [33].

Moringa can be cultivated for its immature seed pods (called “drumsticks”), leaves, and/or seeds/kernels used for oil extraction and water purification [28,33,34]. Apart from the green fruits/seed pods, the leaves (leaflets, stalks, and stems), seeds, flowers, and roots are edible as well [28,34,35]. Indeed, moringa has numerous applications in cooking across world regions [34]. The leaves are considered the most nutritious part of moringa, being a significant source of vitamins (e.g., B, C, K, beta-carotene/provitamin A) as well as minerals (e.g., manganese) and proteins [35,36,37,38]. For this, ground seeds of moringa have been used for the fortification of various foods (e.g., wheat flour) to increase their content of proteins and minerals (e.g., iron, calcium) [34,39,40,41,42]. Mature seeds as well as young fruits/pods can be used to produce an edible oil called ben oil for its high concentration of behenic acid [43]. The cake of seeds, resulting from oil extraction, can be used as a fertilizer/compost or for water purification [44]. Indeed, the seed cake is used, thanks to the flocculation process, to produce potable water [45,46]. Moringa seeds include dimeric cationic proteins [47] that absorb water impurities and colloidal particles, which are then removed as sludge by settling or filtration. This application of moringa, which is accessible and affordable, is particularly relevant in many remote, developing regions where moringa is grown that have no access to potable water networks [46]. The oil extracted from moringa seed can also be used as a biofuel [48]. Furthermore, different parts of the plant (cf. bark, sap, roots, leaves, seeds, and flowers) have been used in traditional, folk medicine [28,49,50,51,52,53,54,55,56,57,58,59,60,61,62]. In this regard, Xiao et al. [63] argued that “*Moringa can not only eliminate pathogens, including bacteria, fungi, viruses, and parasites, but also inhibit chronic inflammation, such as asthma, ulcerative colitis, and metabolic diseases*”. Moringa has been used traditionally in medicine to treat various diseases such as anemia, diabetes, cardiovascular diseases, and various infectious [53] as well as paralysis, helminthiasis, sores, and skin infections [54]. Different analgesic, antihypertensive, antioxidant, anti-inflammatory, diuretic, hepatoprotective, antimicrobial, antiviral, neuroprotective, and anti-cancer pharmacological properties have been attributed to *M. oleifera* [59,60,64]. The uses of moringa also include as a micronutrient, a natural anthelmintic, an adjuvant, or even a fodder/feed for livestock [62,65,66,67,68].

Its versatility and diverse applications and uses make moringa particularly interesting for developing countries, such as African ones [50]. Indeed, it has the potential to boost food security, improve nutrition, support sustainable land management and land reclamation, and foster rural development [28,50,69,70,71]. Moringa has been used successfully to combat malnutrition, especially among children and lactating women [28,35]. In this regard, Keatinge et al. [72] stressed that moringa can play a central role in the attainment of the United Nations’ Sustainable Development Goal 2 “Zero hunger”. Since the plant thrives in harsh conditions, including arid and semiarid environments, it can provide a year-round versatile and nutritious source of food [73]. Therefore, more than 140 organizations have initiated worldwide programs for the cultivation of moringa for malnutrition eradication, water purification, and/or the production of cooking oils [28]. 

Despite its huge potential, the previous reviews that dealt with moringa in Africa (Appendix B) are generally old and/or with partial coverage from both geographical and topical/thematic points of view. Furthermore, no one combined bibliometric and content/topical analyses for the whole African continent. Therefore, there is no recent systematic review, which denotes a concrete research gap. In this context, the present systematic review analyzes the landscape of research on moringa in Africa. It provides an overview of both the bibliographical metrics and the topics addressed in the scholarly literature.

## 2. Materials and Methods

This systematic review follows the guidelines of PRISMA (Preferred Reporting Items for Systematic Reviews and Meta-Analyses) [74,75]. It is based on a search carried out on the Web of Science Core Collection on April 7, 2023. The search was performed using the following search string: *(moringa OR* “*M. oleifera*” *OR* “*drumstick tree*” *OR* “*horseradish tree*” *OR* “*ben oil tree*” *OR* “*benzolive tree*”*) AND (Africa OR Algeria OR Angola OR Benin OR Botswana OR* “*Burkina Faso*” *OR Burundi OR* “*Cabo Verde*” *OR* “*Cape Verde*” *OR Cameroon OR* “*Central African Republic*” *OR Chad OR Comoros OR Congo OR* “*Côte d’Ivoire*” *OR* “*Ivory Coast*” *OR* “*Democratic Republic of the Congo*” *OR Djibouti OR Egypt OR* “*Equatorial Guinea*” *OR Eritrea OR Ethiopia OR Eswatini OR Gabon OR Gambia OR Ghana OR* “*Guinea-Bissau*” *OR Guinea OR Kenya OR Lesotho OR Liberia OR Libya OR Madagascar OR Malawi OR Mali OR Mauritania OR Mauritius OR Morocco OR Mozambique OR Namibia OR Niger OR Nigeria OR Rwanda OR* “*São Tomé and Príncipe*” *OR Senegal OR Seychelles OR* “*Sierra Leone*” *OR Somalia OR* “*South Africa*” *OR* “*South Sudan*” *OR Sudan OR Swaziland OR Tanzania OR Togo OR Tunisia OR Uganda OR Zambia OR Zimbabwe)*. The search on WoS returned 553 potentially eligible documents. The selection of eligible documents was informed by the methodology adopted by El Bilali [76] and El Bilali et al. [77]. 

Table 1 describes the various steps of the selection process. Specifically, three inclusion/eligibility criteria were taken into account: geographical coverage (viz. the eligible document deals with at least one African country); thematic focus (viz. the eligible document deals with *Moringa oleifera*); and document type (viz. only research articles, chapters, or conference papers were considered eligible; editorials, letters to editors, and commentaries/notes, as well as reviews, were excluded). Following the screening of titles, 17 documents were excluded because they dealt with non-African countries mainly from South and Southeast Asia. Further 134 documents were excluded after the analysis of abstracts as they did not meet at least one of the eligibility/inclusion criteria: 53 did not deal with Africa; 69 dealt with other species of moringa, e.g., *Moringa stenopetala*, *Moringa peregrina*, or *Moringa ovalifolia* (articles dealing with *Moringa pterygosperma*, which is considered erroneously a synonym of *M. oleifera*, were considered eligible); and 12 were editorial materials. Finally, the analysis of full texts led to the exclusion of 103 documents, including 47 reviews [35,37,38,41,42,50,51,52,53,54,55,56,57,58,59,60,61,63,64,67,70,71,72,78,79,80,81,82,83,84,85,86,87,88,89,90,91,92,93,94,95,96,97,98,99,100,101].

Therefore, 299 articles (Appendix C) were included in this systematic review, including 270 journal articles and 29 proceeding papers, and underwent analyses. First, the bibliographical metrics and the geography of the research field were analyzed. Then, the selected records were interrogated in relation to different topics (Table 2).

The systematic review conducted had some *limitations* that need to be taken into account. Firstly, the selection of the Web of Science database for the search process implies that only high-quality scholarly literature was considered. However, in turn, this means that grey literature (e.g., reports) and research published in journals not indexed in WoS were not taken into account. Secondly, the search terms used could have affected the results obtained, although various synonyms were used to broaden the initial screening basis. Despite these limitations, this research is noteworthy as it is the first of its kind and provides a starting point for future studies and research projects on *M. oleifera* in Africa and other regions.

## 3. Results and Discussion

### 3.1. Bibliometrics of Research on Moringa in Africa 

The preliminary results of the analysis suggest that Africa has a low share of the research on moringa. Indeed, a search carried out on the same date (7 April 2023) on the Web of Science Core Collection but without making any reference to Africa [viz. (*moringa OR* “*M. oleifera*” *OR* “*drumstick tree*” *OR* “*horseradish tree*” *OR* “*ben oil tree*” *OR* “*benzolive tree*”*)*], thus considering all articles that potentially dealt with moringa worldwide, yielded 5422 documents. Considering that the specific search dealing with the scholarly literature addressing moringa in Africa returned just 553 records, this implies that research in Africa represents only about 10% of the scholarly literature on moringa worldwide. 

Research on *M. oleifera* is rather recent in Africa. Indeed, the first publication dates back to 1995 [104]. However, analyzed data suggest an increase in interest in the subject matter, manifested by an increase in the annual output of articles. Indeed, during the considered period (1995–2023), the annual *output* of papers ranged from 1 in 1995 and 1996 and 0 in some years during the 1990s (cf. 1997) and at the beginning of the century (2001, 2004, 2005) to 31 publications in 2017, 41 in 2020, and a peak of 56 in 2021. Thirteen articles were published in 2015; this is, at least to a certain extent, thanks to the 1st International Symposium on Moringa that was held on November 15, 2015, in Manila (the Philippines). Similarly, the peak reached in 2021 might have been due to the organization of the 2nd International Symposium on Moringa in Pretoria (South Africa), whose proceedings were published in *Acta Horticulturae* in 2021. 

As for *sources and journals* (Table 3), the most important ones were *Acta Horticulturae* (27 articles, 9.03%), *South African Journal of Botany* (21 articles, 7.02%), *1st International Symposium on Moringa* (13 articles, 4.35%), *Journal of Ethnopharmacology* (9 articles, 3.01%), and the *Egyptian Journal of Chemistry* and *2nd International Symposium on Moringa* (7 articles/2.34% each). In a bibliometric analysis carried out in 2021 using the Scopus database, George et al. [85] found that the *South African Journal of Botany* leads worldwide in terms of publication number. The selected 299 publications were published in 182 sources and journals, which suggests that, so far, there is no earmarked publication outlet.

Concerning the *subject areas*, most of the eligible publications fell under the areas of Plant sciences (56 articles, 18.73%), Food science technology (36 articles, 12.04%), Horticulture (30 articles, 10.03%), Agronomy (24 articles, 8.03%), Integrative complementary medicine (22 articles, 7.34%), Pharmacology—pharmacy (21 articles, 7.02%), and Environmental sciences (20 articles, 6.69%). However, the selected articles could be categorized into 67 subject areas (e.g., chemistry, nutrition dietetics, forestry, biology, biochemistry, microbiology, ecology, chemical engineering, environmental engineering, environmental studies), which may explain why the research field is rather multidisciplinary. The multidisciplinary and multisectoral character of the research field is confirmed by the numerous *Sustainable Development Goals* (SDGs) that it addresses; indeed, the selected articles dealing with moringa addressed 11 SDGs, but the most prominent ones were SDG 3 “Good health and well-being” (132 articles, 44.147%), SDG 2 “Zero hunger” (60 articles, 20.07%), SDG 6 “Clean water and sanitation” (56 articles, 18.73%), and SDG 13 “Climate action” (53 articles, 17.73%). Notwithstanding, it can be argued that the focus of the literature was on biological and environmental sciences (including agronomic sciences), while social sciences and economics were underserved. This is corroborated by the study of George et al. [85], who concluded, following their bibliometric analysis on moringa, that “*The research areas on the plant are mainly on the pharmacological, food and nutritional uses as well as its applications in water treatment, environmental management and biofuel production*” (p. 12).

The bibliometric analysis showed that the most prominent, productive authors (six articles each) were based in South Africa, namely, Elsa S. du Toit (Department of Plant and Soil Sciences, Faculty of Natural and Agricultural Sciences, University of Pretoria, South Africa) and Ashwell R. Ndhlala (Vegetable and Ornamental Plants Institute, Agricultural Research Council, South Africa). Nevertheless, the analysis also suggested that there is no consistency in the research field as many authors have only one article. Indeed, the 299 selected articles were authored by 1199 scholars. This means that even authors that deal with moringa do this in a sporadic, rather than a systematic, way. This, in turn, might be due to the absence of structured, articulated national research projects/programs on moringa.

The analysis of *affiliation institutions and countries* suggested that aside from some international organizations such as CGIAR (Consultative Group for International Agricultural Research), the most active centers in the research field are based in Egypt (Egyptian Knowledge Bank, National Research Centre, Agricultural Research Center Egypt, Cairo University, Zagazig University) and South Africa (University of Kwazulu Natal, University of Pretoria, Agricultural Research Council of South Africa, University of Limpopo, University of Fort Hare, North West University South Africa, University of Zululand). Prominent African research centers dealing with moringa are also located in Ghana (Kwame Nkrumah University Science Technology, University of Ghana, University for Development Studies), Malawi (University of Malawi), Nigeria (Obafemi Awolowo University, University of Nigeria, University of Ibadan, Bayero University), Benin (University of Abomey Calavi), and Botswana (Botswana University of Agriculture and Natural Resources). However, some research centers and universities are based outside Africa, especially in Germany (University of Bonn). The 299 selected articles were authored by scholars affiliated with 426 research centers and universities. The results concerning the affiliation institutions are corroborated by those regarding affiliation *countries*. Indeed, the list of affiliation countries was dominated by South Africa (66 articles, 22.07%), Nigeria (50 articles, 16.72%), Egypt (34 articles, 11.37%), and Ghana (22 articles, 7.36%). This result is only partly in line with the findings of George et al. [85], who pointed out Egypt, Nigeria, and India as the countries with the best international linkages in the research field, but not South Africa. However, the top ten countries included two other African countries (viz. Tanzania, Kenya) and, especially, many non-African countries (viz. USA, England, France, Germany). The list of the 71 affiliation countries included many others from Africa (e.g., Ethiopia, Burkina Faso, Malawi, Cameroon, Botswana, Tunisia, Uganda, Algeria, Benin), Europe (e.g., Portugal, Scotland, Belgium, Italy), and even Oceania (e.g., Australia) and North America (e.g., Canada).

As for *funding agencies*, apart from some South African ones (e.g., National Research Foundation South Africa, University of Pretoria, University of Kwazulu Natal, College of Health Sciences Durban, South African Medical Research Council) that ensured domestic funding, most funding came from international organizations (e.g., CGIAR) and foundations (e.g., Bill Melinda Gates Foundation) or foreign institutions, particularly those in Europe (e.g., European Union; *Fundação para a Ciência e a Tecnologia*, Portugal; Federal Ministry of Education Research, Germany; *Deutscher Akademischer Austausch Dienst* DAAD, Germany; the Spanish Government; the UK Research Innovation; Alexander von Humboldt Foundation, Germany) and North America (e.g., United States Agency for International Development (USAID), USA; International Development Research Centre (IDRC), Canada). 

### 3.2. Geography of the Research on Moringa in Africa 

The analysis showed that there are considerable differences among African countries (Table 4). Most studies were carried out in a few countries that dominated the list of affiliation countries, namely, South Africa, Nigeria, Egypt, and Ghana. Indeed, the lion’s share of studies on moringa were carried out in South Africa (55 articles, 18.39% of those selected), Nigeria (51 articles, 17.05%), Egypt (35 articles, 11.70%), Ghana (22 articles, 7.35%); these four countries alone made up more than half of all the studies on moringa in Africa. While the results regarding Nigeria and Egypt were somewhat expected, as these are among the most populous countries in Africa, those regarding South Africa and Ghana might denote a certain dynamism of their research systems. In contrast, many African countries were only marginally addressed in the research field; for instance, in many countries (viz. Central African Republic, Chad, Eswatini/Swaziland, Liberia, Libya), there was only one study on moringa. No article focused specifically on moringa in 22 African countries (viz. Angola, Burundi, Cabo Verde, Comoros, Congo, Djibouti, Equatorial Guinea, Eritrea, Gabon, Gambia, Guinea, Lesotho, Mali, Mauritania, Morocco, Namibia, Rwanda, São Tomé and Príncipe, Seychelles, Somalia, South Sudan, and Zambia). Therefore, there appears to be a significant deficiency in research related to this field in the countries in question.

While there were no studies on moringa that covered the entire African continent, there were some multi-country, regional studies available. For instance, Kumssa et al. [105] investigated the challenges and opportunities for growers of moringa (*M. oleifera* and *M. stenopetala*) in Eastern Africa (cf. Ethiopia and Kenya). de Saint Sauveur et al. [106] provided an overview of the use of moringa leaves to fight against malnutrition and micronutrient deficiencies in several West African countries (cf. Burkina Faso, Ghana, and Senegal). Further studies were rather global, thus dealing with countries from different continents, including some non-African countries; for instance, Sibeko and Johns [107] performed a global survey on medicinal plants used by women during lactation and postpartum recovery and analyzed their health implications. 

**Table 4:** Geography of research on moringa in Africa.

Country or Region (Number of Articles)	Documents
Algeria (5)	Boukandoul et al. [108]; Boulaadjoul et al. [109]; Braham et al. [110]; Kebaili et al. [111]; Ouahrani et al. [112]
Benin (11)	Adéyèmi et al. [113]; Affonfere et al. [114]; Agoyi et al. [115]; Bachabi et al. [116]; Dougnon and Ito [117]; Gandji et al. [118]; Gandji et al. [119]; Noulèkoun et al. [120]; Noulèkoun et al. [121]; Noulèkoun et al. [122]; Noulèkoun et al. [123]
Botswana (5)	Bombo et al. [124]; Nduwayezu et al. [125]; Ntshambiwa et al. [126]; Seifu and Teketay [127]; Semanka et al. [128]
Burkina Faso (6)	Amante et al. [129]; Amante-García et al. [130]; Etongo et al. [131]; Krieg et al. [132]; Lopez-Grimau et al. [133]; Moussavou et al. [134]
Cameroon (10)	Arsene et al. [135]; Mawouma et al. [136]; Ngounouno et al. [137]; Rébufa et al. [138]; Saha et al. [139]; Tchangoue et al. [140]; Yannick et al. [141]; Yongabi et al. [142]; Yongabi et al. [143]; Yongabi et al. [144]
Central African Republic (1)	Poumaye et al. [145]
Chad (1)	Leone et al. [146]
Côte d’Ivoire (2)	Abrogoua et al. [147]; Kpan et al. [148]
Democratic Republic of the Congo (4)	Kambashi et al. [149]; Kampemba et al. [150]; Mushagalusa Kasali et al. [151]; Tshingani et al. [152]
Egypt (35)	Abdalla et al. [153]; Abdallah et al. [154]; Abdel Moniem et al. [155]; Abdelsayed et al. [156]; Abdelwanis et al. [157]; Abdelwanise et al. [158]; Darwish et al. [159]; Desoky et al. [160]; El-Boraie et al. [161]; El-Hadidy et al. [162]; El-Helaly [163]; Ezzo et al. [164]; Hamada et al. [165]; Hegazi et al. [166]; Hemdan et al. [167]; Ibrahim and Abdalla [168]; Ibrahim and Namich [169]; Ibrahim et al. [170]; [171] c Merwad [172]; Merwad [173]; Merwad [174]; Merwad and Abdel-Fattah [175]; Mohamed et al. [176]; Mohamed et al. [177]; Mohamed et al. [178]; Monir et al. [179]; Mosa et al. [180]; Nofal et al. [181]; Nofal et al. [182]; Osman and El-Naggar [183]; Salama et al. [184]; Sarhan et al. [185]; Shaaban et al. [186]; Thabet et al. [187]
Eswatini/Swaziland (1)	Zubuko et al. [188]
Ethiopia (12)	Adefa and Tefera [189]; Bartíková et al. [190]; Debela and Tolera [191]; Ejeta et al. [192]; Gebrezgi [193]; Gurmu et al. [194]; Habtemariam et al. [195]; Mekuria et al. [196]; Melesse et al. [197]; Němec et al. [198]; Tafesse et al. [199]; Tafesse et al. [200]
Ghana (22)	Acheampong et al. [201]; Adu-Dapaah et al. [202]; Amaglo et al. [203]; Amagloh and Benang [204]; Amagloh et al. [205]; Boateng et al. [206]; Boateng et al. [207]; Clark et al. [208]; Cudjoe et al. [209]; Doglikuu et al. [210]; Glover-Amengor et al. [211]; Kudzinawo et al. [212]; Kumah et al. [213]; Mao et al. [214]; Ntibrey et al. [215]; Ochire-Boadu et al. [216]; Peprah et al. [217]; Sarkwa et al. [218]; Senanu et al. [219]; Sengupta et al. [220]; Tabong et al. [221]; Tetteh et al. [222]
Guinea-Bissau (4)	Bancessi et al. [223]; Bancessi et al. [224]; Fernandes et al. [225]; Fernandes et al. [226]
Kenya (6)	Chang et al. [227]; Kamau et al. [228]; Kilingo et al. [229]; Muluvi et al. [230]; Walter et al. [231]; Waterman et al. [232]
Liberia (1)	Awoyale et al. [233]
Libya (1)	Mahklouf [234]
Madagascar (3)	Rakotosamimanana et al. [235]; Rubio [236]; Syeda and Riazunnisa [237]
Malawi (10)	McConnachie et al. [238]; Mwamatope et al. [239]; Mwamatope et al. [240]; Pritchard et al. [241]; Pritchard et al. [242]; Pritchard et al. [243]; Pritchard et al. [244]; Sagona et al. [245]; Vunain et al. [246]; Warhurst et al. [247]
Mauritius (2)	Mahomoodally and Ramalingum [248]; Neergheen-Bhujun et al. [249]
Mozambique (2)	Cumbe et al. [250]; Marrufo et al. [251]
Niger (6)	Abasse et al. [252]; Bellostas et al. [253]; Freiberger et al. [254]; Halilou et al. [255]; Ratnadass et al. [256]; Sena et al. [257]
Nigeria (51)	Aba et al. [258]; Abubakar et al. [259]; Abubakar et al. [260]; Adeyemi et al. [261]; Adeyemo [262]; Aduro and Ebenso [263]; Agbagwa et al. [264]; Agbede et al. [265]; Agbede et al. [266]; Ajuogu et al. [267]; Akanbi et al. [268]; Amaeze et al. [269]; Amaeze et al. [270]; Amaeze et al. [271]; Anele et al. [272]; Anjorin et al. [273]; Aprioku et al. [274]; Arigbede et al. [275]; Atawodi et al. [276]; Attah et al. [277]; Aviara et al. [278]; Ayotunde et al. [279]; Barminas et al. [280]; Ebiloma et al. [281]; Etuk and Mohammed [282]; Fagbenro et al. [283]; Gambo et al. [284]; Gambo et al. [285]; Gambo et al. [286]; Iwuji et al. [287]; Jimoh et al. [288]; Lawal et al. [289]; Lockett et al. [290]; Mahdy et al. [291]; Manuwa et al. [292]; Namla et al. [293]; Nwosu and Okafor [104]; Obayelu et al. [294]; Ogunbinu et al. [295]; Ogundiran et al. [296]; Ojo et al. [297]; Okoya et al. [298]; Oladeji et al. [299]; Oladeji et al. [300]; Olaoye et al. [301]; Oyekale [302]; Popoola and Obembe [303]; Stevens et al. [304]; Taiwo et al. [305]; Titiloye et al. [306]; Ugese and Iordye [307]
Senegal (6)	Dièye et al. [308]; Diouf et al. [309]; Faye et al. [310]; Gold et al. [311]; Mathieu and Meissa [312]; Zeitoun et al. [313]
Sierra Leone (2)	James et al. [314]; Ranasinghe et al. [315]
South Africa (55)	Adetola et al. [316]; Adhikari et al. [317]; Adhikari et al. [318]; Bassey et al. [319]; Bopape-Mabapa et al. [320]; Chitiyo et al. [321]; du Toit et al. [322]; du Toit et al. [323]; du Toit et al. [324]; Govender and Naicker [325]; Hedhili et al. [326]; Ibrahim et al. [327]; Kivevele and Huan [328]; Lubaale et al. [329]; Mabapa et al. [330]; Maiyo et al. [331]; Managa et al. [332]; Manduwa et al. [333]; Masete et al. [334]; Mhlomi et al. [335]; Modisaojang-Mojanaga et al. [336]; Moichela et al. [337]; Mokgehle et al. [338]; Motis et al. [339]; Moyo et al. [340]; Mudau et al. [341]; Muhl et al. [342]; Naidoo [343]; Ndhlala et al. [344]; Netshiheni et al. [345]; Ngcobo and Bertling [346]; Ntila et al. [347]; Ntila et al. [348]; Olusanya et al. [349]; Omodanisi et al. [350]; Pakade et al. [351]; Pakade et al. [352]; Ralepele et al. [353]; Rapatsa and Moyo [354]; Ratshilivha et al. [355]; Ratshilivha et al. [356]; Rikhotso et al. [357]; Rikhotso et al. [358]; Sebola et al. [359]; Semenya and Maroyi [360]; Semenya and Maroyi [361]; Semenya et al. [362]; Sharaf-Eldin et al. [363]; Swanepoel [364]; Tiloke et al. [365]; Tiloke et al. [366]; Tshabalala et al. [367]; Tshabalala et al. [368]; Zeru et al. [369]; Zungu et al. [370]
Sudan (2)	Ali et al. [371]; Ezaldeen et al. [372]
Tanzania (12)	Alphonce et al. [373]; Alphonce et al. [374]; Bisanz et al. [375]; Hekmat et al. [376]; Kibazohi and Sangwan [377]; Lunyera et al. [378]; Marobhe and Sabai [379]; Mmanda et al. [380]; Munyanziza and Sarwatt [381]; Ndemanisho et al. [382]; Sarwatt et al. [383]; Shija et al. [384]
Togo (2)	Abotsi et al. [385]; Fare et al. [386]
Tunisia (6)	Bennour et al. [387]; Bennour et al. [388]; Boumenjel et al. [389]; Gharsallah et al. [390]; Marzougui et al. [391]; Marzougui et al. [392]
Uganda (3)	Jilcott et al. [393]; Kaggwa et al. [394]; Kasolo et al. [395]
Zimbabwe (4)	Choga et al. [396]; Jambwa et al. [397]; Maroyi [398]; Smith et al. [399]
East Africa * (2)	Kumssa et al. [400] (Ethiopia and Kenya); Kumssa et al. [105] (Ethiopia and Kenya)
West Africa ** (1)	de Saint Sauveur et al. [106] (Burkina Faso, Ghana, and Senegal)
Sub-Saharan Africa *** (4)	Chadha and Olouch [401] (Kenya, Malawi, Mozambique, Rwanda, Southern Sudan, Tanzania, Uganda, and Zambia); Chadha et al. [402]; Ondiba et al. [403]; Yamato et al. [404] (Madagascar and Uganda)
Global ****(4)	Ebert and Palada [405]; Meale et al. [406]; Palada et al. [407]; Sibeko and Johns [107]

* Includes articles addressing at least two East African countries. ** Includes articles addressing at least two West African countries. *** Includes articles dealing with at least two countries from two different regions in Sub-Saharan Africa. **** Includes articles covering at least one country outside Africa.

### 3.3. Agriculture Subsectors and Food Chain Stages

As for the *agriculture subsectors*, while, as expected, most of the selected articles dealt with crop production, with *M. oleifera* being a crop/plant, some addressed animal production and fisheries/aquaculture. The latter mainly referred to the use of moringa as fodder for livestock or feed in aquaculture/fish farming. Indeed, moringa has been used as feed or fodder for many livestock species across Africa [149,165,218,250,336,340,359,369,382,383,397], e.g., for sheep in Ghana [218]; for pigs in the Democratic Republic of the Congo [149]; for cattle in Mozambique [250]; for goats in South Africa [340] and Tanzania [382,383]; and for chicken/poultry in South Africa [336,359], Egypt [165], and Zimbabwe [397]. Similarly, different parts of moringa, especially the leaves, have been used as feed in aquaculture and fish farming [113,179,279,354,380,403], e.g., for Tilapia in Tanzania [380], Benin [113], Egypt [179], Nigeria [279], and South Africa [354]. Moringa has also been used as an ethnoveterinary remedy against avian diseases in Zimbabwe [397] and tested against parasitic flatworm (*Fasciola gigantica*) in Egypt [166].

Production and consumption were the most addressed *stages of the food chain*, while intermediate stages, especially marketing and distribution, were generally overlooked in the scholarly literature (Table 5). A limited number of studies specifically addressed the processing of moringa. For instance, Manuwa et al. [292] designed, produced, and evaluated the performance of a pulverizer of the dried leaves of moringa in Nigeria. However, many studies referred to the use of processed products of *M. oleifera*. Processed products of moringa include leaf powder [114,286,329,399], seed oil [108,157,278,319,390], seed cake [162,167,306], seed powder [116,128,215,246], and essential oils from leaves [251]. Further studies dealt with the use of moringa as an ingredient in different processed foods, e.g., smoked African mud catfish [288], chilled meat [165], and maize chips [325]. Moringa has also been used to improve the conservation of some perishable products such as sweet oranges [307]. Indeed, moringa is believed to reduce microbial contamination and conserve the quality and extend the shelf-life of agri-food products. Only a few papers dealt with the marketing of moringa and its products. For instance, Ojo et al. [297] analyzed the commercialization potential and factors affecting it among moringa farmers in southwestern Nigeria. Similarly, Seifu and Teketay [127] explored the potential for the commercialization of moringa in Botswana. Consumption referred to the use of moringa as food but also as a medicine/drug. Indeed, the lion’s share of articles included in this category addressed the use of moringa in traditional, folk medicine against a wide range of diseases and ailments. Some authors adopted a more holistic approach and dealt with different food chain stages. Indeed, they referred to supply chains or value chains [202,386]. For instance, Adu-Dapaah et al. [202] provided an overview of recent advances and current practices used by stakeholders along the value chain of moringa in Ghana from seed production to planting, processing, and marketing.

Moringa has been grown in a variety of *agricultural systems* ranging from horticulture (monocropping or intercropping) [256,342] to agroforestry [105,115,118,119,120,141,191,197,202,216,272,383,385,389,407]. It has been indicated as suitable for intercropping with vegetables, fruit trees, and medicinal and aromatic plants in South Africa [364] and legumes (cowpea, jack bean, lablab, and pigeon pea) in South Africa [339]. As for agroforestry, moringa has been investigated in Benin [118,119,120], Cameroon [141], Ethiopia [105,191,197], Ghana [202,216], Kenya [105], Nigeria [272], Tanzania [383], Togo [385], and Tunisia [389]. In this respect, Boumenjel et al. [389] concluded that “*Moringa represent a promising species as an ecological solution for use in agroforestry systems, able to minimize the negative effects of drought and to rehabilitate and enhance the soil of arid zones*” (p. 823). Moringa has been also used in alley cropping/farming in Ghana [202], Nigeria [258], South Africa [339], and West Africa [407].

As for *production*, the agronomic practices considered included fertilization and soil fertility management, pest management, and irrigation (Table 6). Concerning, for instance, irrigation, El-Boraie et al. [161] studied the effects of different modern irrigation systems (e.g., drip irrigation, mini-sprinklers) on water unit productivity of moringa vegetative yield under the conditions of Shalatien (Egypt). As for pest management, the analyzed studies dealt with, among others, the moringa tree defoliator, *Noorda blitealis* Walker (Lepidoptera), in Niger [255], powdery mildew in Ethiopia [190], and the leaf worm in the Sudano-Sahelian agroecosystems of West Africa [256]. While moringa is known to be pest-tolerant, its tolerance to pests seems to change from one species to another. For instance, Němec et al. [198] found that *M. oleifera* is more suitable than *M. stenopetala* for plantations in southern Ethiopia given its higher pest resistance and leaf biomass production. Further documents dealt with the germination of moringa seeds under different conditions [322,363,392] as well as the vegetative propagation of moringa [356].

While some articles dealt with the fertilization of moringa (Table 6), others addressed the use of moringa as a fertilizer or soil amendment. Indeed, moringa extracts (especially leaf extracts) as well as moringa seed cake have been also used as fertilizers and soil amendments on various crops across the African continent. It has been also used as a green manure (Table 7). The results are promising; for instance, Mosa et al. [180] found that spraying moringa leaf extract (MLE) “*improved the vegetative growth, fruit set %, fruit yield and fruit physical and chemical characteristics as well as leaf nutritional status*” of apple in Egypt.

As in the case of fertilization, while some studies focused on the management of moringa pests and diseases, others dealt with the use of moringa-based products against various plant pests. Indeed, extracts from different parts of moringa have been used to control various pests and diseases across Africa. The main pests and diseases studied include the late blight (*Phytophthora infestans*) on tomato in Zimbabwe [396], the charcoal rot and root-knot nematode on faba bean in Egypt [178], bacteria (*Pantoea* spp. and *Sphingomonas* spp.) on rice in Benin [116], the crown rot disease on banana in Ghana [213], and the citrus nematode (*Tylenchulus semipenetrans*) on citrus, grape, olive, loquat, and persimmon in Egypt [170]. Interestingly, moringa extracts have also been used as additives to prolong the activity of some bio-pesticides, such as baculovirus in Egypt [163]. While the green fruits/seed pods, leaves, seeds, flowers, and roots of moringa are edible [28,34,35], most studies focused on leaves (Appendix D). The focus on leaves might be explained by the fact that they are considered the most nutritious part of the plant [35,36,37,38]. However, the part of concern depended on the focus of the studies; leaves were generally considered in studies dealing with food and nutrition (including food supplementation and fortification) and those dealing with traditional medicine, while seeds were considered in studies dealing with the purification and treatment of water. Articles dealing with agronomic aspects generally considered the whole moringa plant, without any distinction between its different parts. Some studies dealt simultaneously with different parts of moringa. For instance, Abdelwanis et al. [157] analyzed the effects of the foliar application of zinc and boron on the yield and chemical composition of leaves and the characteristics of the seed oil. Stevens et al. [304] evaluated the nutrient and vitamin contents of the leaves and seeds of 10 accessions of *M. oleifera* from across Nigeria. Fernandes et al. [225] investigated the nutritional and phytochemical profiles as well as the biological activities of the edible parts of moringa (viz. flowers, fruits, and seeds) in Guinea-Bissau. In their analysis of the potential of *M. oleifera* in Botswana, Seifu and Teketay [127] underscored that “*Different parts of the tree are used to treat, mainly, diabetes, high blood pressure and rheumatism. The leaves are used to treat most of these ailments although the seeds, stems, bark, roots and fruits have also been reported to have medicinal values*” (p. 471). Referring to the medicinal uses of moringa in southern Benin, Agoyi et al. [115] found that “*among the plant parts used for this purpose, the leaf was the most used, followed by the roots, bark, seeds and pods*” (p. 303).

Different *varieties* and cultivars of moringa were considered in the analyzed studies such as PKM-1 and Malawi [332], Drumstick tree PKM-1 (from India), RCA Moringa (Tanzania), Marum K (Thailand), Virgin Islands Drumstick (USA), Limpopo (South Africa) [368], KP1 and KP2 [385], TOT5169 (Thailand), and SH (South Africa) [344].

### 3.4. Climate and Ecosystem Resilience

The evidence collected from the analyzed articles suggested that moringa can play a role in addressing many environmental problems and challenges such as climate change, land degradation and desertification, and water and soil pollution (Table 8).

Different articles show that moringa can be a valuable asset in mitigating and/or adapting to *climate change and variability* in Africa. Marzougui et al. [392] argued that “*Considering Tunisian climate conditions, results showed that Moringa oleifera is able to acclimatize to temperatures of this country*” (p. 349). After comparing the gas exchange of three species (viz. moringa, mopane, and manila) in northern South Africa, Mabapa et al. [330] underscored that “*Moringa maintained good leaf yield even under drought condition, indicated its potential to act as a good sink for CO_2_ assimilation. The results strongly showed the superiority of moringa in capturing more carbon among the three species. Moringa can therefore be recommended for climate change mitigation in semi-arid areas of Limpopo province and possibly other areas*” (p. 2669). Amante et al. [129] argued that moringa-based coagulant allowed for water purification in Burkina Faso with lower energy consumption and reduced carbon dioxide emissions. Moringa can be a valuable asset in marginal agro-ecosystems (e.g., dry or saline lands). This is due to, among other things, its tolerance to drought and its ability to grow in harsh conditions. In this respect, Seifu and Teketay [127] stressed that “*The Moringa tree does not need intensive management, establish easily, adapt well to the arid climate of Botswana and grow with minimal care*” (p. 471). Many scholars considered moringa as a drought-tolerant/resistant plant [127,330,381,404]. Furthermore, different articles dealt with the drought tolerance of moringa and/or improving it through some agronomic practices [153,164,321,389,404]. Recently, *M. oleifera* has attracted the attention of many researchers and scholars as a promising methane mitigation strategy [197,327,369,406]. For instance, the extracts of moringa have been successfully used as additives in *Eragrostis curvula* hay to reduce methane production in ruminant nutrition [327]. The production of biofuel/biodiesel from moringa shows that the species can contribute to efforts to mitigate climate change. Moringa has been used or tested as a biofuel feedstock in Botswana [124], Burkina Faso [134], South Africa [328], and Tanzania [377].

Moringa has been also exploited to halt and/or reverse *land degradation* across Africa. Indeed, it has been employed in different afforestation/reforestation endeavors. Studies on this subject matter were mainly carried out in Benin [119,120,121,122,123]. Noulèkoun et al. [121] found that moringa is suitable for the afforestation of degraded croplands in the Sudano-Sahelian zone of Benin. Moringa has been also used for the remediation of contaminated soils across Africa [155]. Working on Paulownia seedlings in Cairo (Egypt), Abdel Moniem et al. [155] concluded that the “*Remediation of soil with defatted seeds of Moringa oleifera had promotion effects on reducing the uptake of metal and decreasing its availability*” (p. 6847). Moringa showed tolerance to abiotic stresses such as salinity in different studies [182,183]. Interestingly, moringa was used to increase the tolerance to salinity of different crops such as cotton [169], wheat [173], and Sudan grass [160,174].

Further studies dealt with the role of moringa in addressing *water and soil pollution*. Indeed, moringa was used in the treatment of sewage sludges, wastewater, effluents, and different types of industrial wastes [111,137,215,219,229,246,311,391]. Studies in this respect, exploiting the potential of *M. oleifera* seeds as coagulants/flocculants, were carried out in Algeria [109,111], Kenya [229], Tunisia [391], Cameroon [137,140], Ghana [201,215,220], and Malawi [246]. Kilingo et al. [229] pointed out that the use of moringa is “*a low-cost sewage treatment technology suitable for poor areas*” (p. 36589). Marzougui et al. [391] found that moringa-treated wastewater “*could be used in agricultural irrigation, leading to less use of chemical nitrogen fertilizers and thus improving sustainability for crops, soils, animals, and humans*”. Vunain et al. [246] added that “*Employing Moringa oleifera seed (as powder or extracts) to treat municipal domestic wastewater effluent presents an alternative practice to improving water quality effluent of existing wastewater treatment plants in developing countries*”.

Moringa has also shown potential in reducing particulate matter air pollution [318]. For instance, Adhikari et al. [318] evaluated the particle trapping abilities of different herbaceous plants and trees, including moringa, to mitigate particulate matter (PM) pollution in Sekhukhuneland, a semi-arid mining region of South Africa. *M. oleifera* was one of the six preferred species and the authors concluded that “*The six preferred plants may serve as forerunner species to abate PM pollution in Sekhukhuneland and other arid regions facing similar climate change and pollution challenges*”.

### 3.5. Food and Nutrition Security

The analyzed scholarly literature suggests that moringa can contribute to food and nutrition security in terms of food availability, food access, food utilization, and food stability.

As for *food availability*, moringa represents an important source of food for an increasing number of households in Africa. Indeed, the cultivation and, consequently, production of moringa are increasing across Africa [105,127].

*Food access* is a marginal topic in the scholarly literature dealing with moringa in Africa. However, evidence shows that households with access to moringa have a better food and nutrition security status. Following a study on the impacts of moringa on food and nutrition security in southern Ethiopia, Tafesse et al. [200] highlighted that “*The finding indicated positive and significant differences among Moringa producer and non-producer smallholder farmers in selected food and nutrition security proxy variables, weekly calorie intake and food consumption score (FCS)*”.

Most of the selected articles that addressed food security in relation to moringa dealt with *food utilization* and nutrition. Indeed, moringa represents a valuable source of nutrients such as minerals and vitamins [126,146,191,203,211,225,254,257,273,280,304,349,370,390,400]. It is also a valuable source of bioactive compounds [126]. However, the nutritional composition of moringa leaves is influenced by many factors such as soil properties, agronomic practices, and tree age [320]. Despite its confirmed nutritional benefits, the consumption of moringa is not adequate; therefore, there is a need to increase its use through changes in consumption and dietary patterns [106].

Given the interesting nutritional profile of moringa, it comes as no surprise that it has been used in *fortification* and supplementation programs in many African countries. Indeed, moringa-based products have been tested and/or used for the fortification or enrichment of nutrients in a wide range of products across Africa. It has also been used as a food supplement (Appendix E). The concerned products include porridges from various cereals (e.g., sorghum, maize, pearl millet) as well as foods based on roots such as cassava and even processed foods such as yogurt. Interventions involving the use of moringa in food fortification and supplementations resulted in an important reduction in anemia among children [114,384].

*Food safety* is an important component of food utilization. In this respect, some authors pointed out that the leaves of moringa can accumulate some pollutants such as heavy metals [189,233,317], which might pose a risk in terms of human health. Moreover, interestingly, given its antimicrobial properties, moringa has also been tested as a hand-washing product [208].

The selected articles also mentioned different *health claims* regarding moringa. These were related to numerous diseases, ailments, and illnesses such as cardiovascular diseases, cancers, diabetes, digestive diseases, infectious diseases, malaria, non-communicable diseases, tropical diseases, venereal diseases, and viral diseases (Table 9). Popoola and Obembe [303] argued that “*All parts of Moringa oleifera are medicinally valuable with overlapping uses in treating myriads of ailments and diseases including body pains and weakness, fever, asthma, cough, blood pressure, arthritis, diabetes, epilepsy, wound, and skin infection. Moringa also has robust ability to challenge terminal diseases such as HIV/AIDs infections, chronic anemia, cancer, malaria and hemorrhage*”. Referring to an ethnobotanical survey in southern Benin, Agoyi et al. [115] stated that “*The study revealed up to 46 complete recipes traditionally made to heal 34 common diseases. These include venereal diseases, cardiovascular diseases, infectious diseases, tropical diseases, inflammatory complaints, oto-rhino-laryngologocal complaints, skin infections, digestive diseases, infertility disorders, etc.*” (p. 303). Different studies suggest that food supplementation with moringa leaves improves the quality of life of people living with HIV [152,274,285]. For instance, Gambo et al. [285] concluded that supplementation with moringa leaves for people living with HIV (PLHIV) improved their quality of life in terms of physical and psychological factors, level of independence, and social relationships. However, some caution is needed, as pointed out by Liu et al. [54]: “*Although M. oleifera has been widely used in traditional medicine, the pharmacological studies that have been conducted so far are not sufficient for its use in the setting of evidence-based medicine. Little relevant data from clinical trials of M. oleifera have been reported. The majority of studies of its constituents, such as carbamates and glucosinolates, have been conducted only in vitro. Owing to a lack of available data, the pharmacology, toxicity, agricultural economy and dietary benefit of its constituents and extracts require further evaluation*”. In particular, only some articles dealt with the toxicity of moringa [303]. While some studies underscored the low toxicity of different parts of moringa, such as the leaves [171], Liu et al. [54] pointed out that moringa is toxic at certain doses and overuse can cause genotoxicity, with a few studies suggesting its hepatotoxicity and nephrotoxicity as well as potential blood toxicity. Jain et al. [83] stressed that “*Largely, the pharmacological applications of various bioactive phytochemicals from M. oleifera have been based on anecdotes and the mechanistic details governing their activities have been elusive*”. That implies that further studies are needed to provide the necessary evidence supporting the use of moringa in medicine.

In the context of food safety, access to clean water is also crucial because water is used in, among other things, food preparation and cooking. In this regard, moringa has shown good potential for the *purification of water* (from wells, reservoirs, etc.). Evidence from the literature suggests that moringa has been successfully used for water purification in different African countries such as Burkina Faso [129,133], Cameroon [143], Ethiopia [195], Ghana [204], Guinea-Bissau [224], Ivory Coast [148], Malawi [242,243,244,247], Nigeria [263], and Tanzania [379]. Apart from water purification for use as drinkable, potable water, moringa seeds have been used for the treatment of surface water [144].

While only a few papers dealt with the *stability* pillar of food security, it is important to underscore the role of moringa in providing food to the population during the lean season and drought periods [290], when a limited array of foods are available to households, especially in arid and semi-arid regions. For that, moringa has been considered as one of the “famine foods” in, among others, Niger [254] and Kenya and Ethiopia [105]. Furthermore, moringa used in agroforestry systems [120] can contribute to the stability of food production in the context of climate change.

### 3.6. Socio-Economic Impacts

Some studies, although limited, suggested that moringa contributes to rural livelihoods and income generation, especially for small-scale farmers.

Many scholars underscored moringa as an important livelihood strategy and source of income in countries such as Ghana [212], Ethiopia and Kenya [105], Burkina Faso [131], Zimbabwe [398], Kenya [232], Senegal [309], and Niger [252]. For instance, Kudzinawo et al. [212] highlighted the role of moringa in income generation and job creation in the Bono East Region (Ghana). In this context, growing moringa was also considered as a poverty reduction strategy [212]. Ebert and Palada [405] assumed that moringa contributed not only to the improved nutrition and health of smallholder farmers in developing countries but also to their improved livelihoods and income. Likewise, Chadha et al. [402] argued that the promotion of indigenous vegetables (including moringa) is useful not only for health and food security but also for income generation in Africa.

Moringa seems particularly relevant for poor people. Apart from being a source of income, it helps offset the low quality of services such as healthcare [326] and drinkable water [311]. The relevance of moringa for health and income generation makes it suitable in gardening kits that have been used in development and aid interventions in different countries in Sub-Saharan Africa (e.g., Kenya, Malawi, Mozambique, Rwanda, Southern Sudan, Tanzania, Uganda, Zambia) [401].

Some studies suggested that moringa can contribute to gender and youth empowerment [212,401]. For instance, Kudzinawo et al. [212] argued that the encouragement of the cultivation of moringa in the Ghanaian region of Bono East can “*lead to nutritional security, youth empowerment and liberation of villages and rural communities from intense poverty*” (p. 216).

Therefore, the development of value chains of moringa can be a valuable asset to address various challenges, thus contributing to sustainable rural development in Africa. For instance, Amaglo et al. [71] pointed out that “*Identifying innovative policy approaches and interventions to the commercialization of Moringa oleifera products may enhance the endorsement of moringa as sustainable source of income for rural households and will contribute to better natural resources management. This should be done with special focus on smallholder growers to enhance sustainable agricultural systems*” (p. 455). Similarly, Kudzinawo et al. [212] argued that “*Considering the viability of this plantation and the potential it portrays for sustainable development, income generation, and job creation, we recommend that governments, non-governmental organizations, and donor agencies support, invest, and encourage the cultivation, consumption, and activities of value addition to Moringa oleifera in their programs*” (p. 216).

### 3.7. Constraints to the Development of Moringa in Africa and Proposed Recommendations

Authors highlighted different *constraints* that hinder the development of moringa. One of the main problems faced by the actors of the value chain of moringa in Africa is the lack of and/or difficult access to high-quality, reliable data and information. Indeed, Ojo et al. [297] underscored that “*Despite the importance of Moringa oleifera tree for food security at the smallholder farmers’ level, there is no consolidated socioeconomic information about the crop such as its contribution to household food security and income, and its production potential, commercialization and constraints*”. So, the development of research as well as extension and advisory services on moringa are paramount to ensure its promotion in Africa. Another challenge is the lack of good-quality seeds. Indeed, referring to the promotion of Indigenous vegetables (including moringa) in Africa, Chadha et al. [402] argued that “*the main constraint to production is availability of quality seed*” (p. 253). Therefore, the development of seed systems, both formal and informal, is crucial to allow the producers to have access to affordable, good-quality seeds of moringa. However, producers and value chain actors face challenges that go beyond access to information and quality seeds. Kumssa et al. [105] found that moringa growers in Ethiopia and Kenya face several challenges, “*including a lack of reliable information on nutritional and medicinal values, inadequate access to markets for their products, and pest and disease stresses to their plants*”. Therefore, the promotion of value addition and the marketing of moringa and its products are crucial. Furthermore, technical advice on the management of pests and diseases seems paramount. Another challenge relates to the negative perceptions about moringa. Indeed, leafy vegetables, such as moringa, are sometimes indicated as “famine foods” [254,400] or foods of the poor. Therefore, there is a need to change perceptions about moringa through awareness-raising and sensitization campaigns. Particular attention should be devoted to the younger generations, so education in schools is important.

The scholars formulated different *recommendations* to develop moringa and exploit fully its potential in Africa. Some recommendations were rather specific and stemmed directly from the results of the studies, while others were more general. Different scholars called for using moringa to address the challenges the African continent is facing. For instance, Ojo et al. [297] recommended that “*for the enormous potential of the moringa tree species and its various products to be harnessed, stakeholders, NGOs, and policymakers should, through adequate extension services, widely promote and scale up the domestication of the miracle tree*” (p. 1129). Kudzinawo et al. [212] stressed the opportunity for supporting, investing, and encouraging the cultivation, use/consumption, and activities of value addition to moringa. Alphonce et al. [374] called for promoting the use of moringa to address micronutrient (iron and vitamin A) deficiencies. Amagloh and Benang [204] suggested using moringa for water treatment in rural areas. Seifu and Teketay [127] recommended expanding the cultivation and utilization of moringa to contribute to food and nutrition security. Mohamed et al. [176] suggested cultivating moringa on a wide scale to benefit from its pharmaceutical and nutraceutical compounds. Mabapa et al. [330] underscored the opportunity of growing moringa for climate change mitigation in semi-arid areas. El-Hadidy et al. [162] recommended using moringa seed cake as a natural organic fertilizer instead of some chemical fertilizers. Ogundiran et al. [296] suggested utilizing moringa for the phytoremediation of soils contaminated with heavy metals. However, public policies are needed for the promotion of moringa. In this respect, Kudzinawo et al. [212] called for paying more attention to moringa in public policies, strategies, and programs for food security and job creation. Moreover, designing public policies alone is not enough, and scholars called for concrete actions relating to research, extension, and industrial development. For instance, Dièye et al. [308] advised developing research on the use of moringa in medicine. Halilou et al. [255] underscored the need to train farmers on the management of moringa pests. Ojo et al. [297] suggested providing adequate extension services to promote and scale up the domestication and cultivation of moringa. Kasolo et al. [395] suggested standardizing moringa leaves used in nutrition and herbal medicine.

Nevertheless, beyond single, fragmented actions, the promotion of moringa in Africa requires an articulated, integrated strategy that spans different domains and sectors. In this context, the following recommendations have been identified for the development and promotion of NUS [14]: changing negative perceptions of NUS as being associated with poverty; building capacity in research, policymaking, marketing, and farming; conducting more research on NUS especially concerning impacts on nutrition and livelihoods; establishing on-farm conservation programs for NUS; engaging all stakeholders, including farmer and women’s organizations, in participatory partnerships to promote and conserve NUS; improving NUS market chains and developing value-added products; implementing legal frameworks, policies, and financial incentives to promote NUS; and encouraging collaboration on NUS and coordinating multi-stakeholder platforms across different sectors. These recommendations are also relevant for moringa in the African context.

## 4. Conclusions

To the best of the authors’ knowledge, this is the first comprehensive, systematic analysis of research on *Moringa oleifera* in Africa. It provides an overview of both the bibliographical metrics and the topics addressed in the scholarly literature.

Research on *M. oleifera* is relatively recent in Africa, with a growing interest shown by the increase in annual research output. The focus is mainly on biological and environmental sciences, with a lack of attention to social sciences and economics. Funding comes mainly from international organizations and foreign institutions. Studies on moringa are concentrated in South Africa, Nigeria, Egypt, and Ghana, while many African countries have no research on the topic. The literature mainly addresses the production and consumption stages of the food chain, while the marketing and distribution stages are often overlooked. Topics include agronomic practices, agricultural systems, intercropping, and the use of moringa-based products for fertilization and pest management. Consumption involves using moringa as food and in traditional medicine. The focus is primarily on the use of the leaves. The evidence suggests that moringa can help address environmental challenges and contribute to food security in Africa. It is a valuable source of nutrients and can be used in fortification and supplementation programs. Moringa also has the potential for water purification and providing food during lean seasons. Additionally, it can contribute to rural livelihoods and income generation, especially for small-scale farmers, and may empower gender and youth in Africa.

Despite its well-documented advantages and benefits, different constraints hinder the development of moringa in Africa. These include the lack of good-quality, reliable data and information; lack of quality seeds; inadequate access to markets; difficulty in managing pests and diseases; and negative perceptions about moringa. Therefore, there is a need to develop research as well as extension and advisory services on moringa, develop formal and informal seed systems, foster access to markets, promote processing and value addition, provide technical advice to producers on the management of pests and diseases, and change perceptions about moringa through education, awareness-raising, and sensitization. Nevertheless, beyond single, fragmented actions, the promotion of moringa requires an integrated strategy. Such a strategy can include building the capacity of all actors in the agricultural knowledge and innovation system (AKIS); conducting more research on moringa, especially regarding impacts on nutrition and livelihoods; establishing community seed banks and on-farm conservation programs; improving markets and value chains; and creating an enabling policy environment. There is also a need to develop and implement a multidisciplinary research program on moringa, with provisions for cross-country and regional collaboration across Africa. Such a regional research program should cover all scientific disciplines, including natural sciences (e.g., agronomic research on the adaptation of moringa to changing climates, poor soils, and dry environments) as well as social sciences and economics. It should also address the relationship between moringa, food security and nutrition, and the livelihoods of rural communities. Attention should also be given to political and governance issues.

## Figures and Tables

**Table 1:** Selection of the eligible documents included in this systematic review.

Selection Step	Number of Potentially Eligible Records	Selection Step Description
Identification of records on the Web of Science (WoS)	553	
Merging of search results	553	No duplicates
Screening of records based on titles	553	17 records excluded because they dealt with non-African countries, e.g., China, India, Israel, Italy, Oman, Pakistan, the Philippines, Taiwan, Thailand, and the United States
Screening of records based on abstracts	536	134 records excluded: 69 records because they did not address *M. oleifera* 53 records because they did not deal with Africa/African countries 12 records because they were editorial materials without abstracts
Scrutiny of full texts	402	103 records excluded: 6 records because they did not address *M. oleifera* 50 records because they did not deal with Africa/African countries 47 records because they were reviews
Inclusion in the systematic review	299	--

**Table 2:** Analyses undergone by the eligible documents on moringa in Africa.

Item	Description	Used Method Reference(s)
Bibliographical metrics	Journals/sources, research/subject areas, SDGs, authors, affiliation institutions/organizations and countries, and funding agencies	El Bilali [102], El Bilali [76], and El Bilali et al. [77]
Research geography	African countries where studies on moringa were performed	El Bilali [76] and El Bilali et al. [77]
Agriculture subsectors	Crop production, animal production/livestock, and fisheries/aquaculture	El Bilali [76] and El Bilali et al. [77]
Food chain stages	Production (agronomy), processing, distribution/retail/marketing, consumption, and waste management	El Bilali [102], El Bilali [76], and El Bilali et al. [77]
Food security and nutrition	Food security dimensions/pillars: availability, access, utilization/use, and stability	El Bilali [103], El Bilali [102], and El Bilali [76]
Climate and ecosystem resilience	Climate change mitigation/adaptation Resilience of farming systems	El Bilali [102]
Socio-economic impacts	Contribution to livelihoods and income generation Gender issues	El Bilali [102]
Constraints and recommendations	Factors hindering the development of moringa in Africa and recommendations	El Bilali [102] and El Bilali [76]

**Table 3:** Bibliographical metrics of the scholarly literature on moringa in Africa.

Journals/Sources (a) *	Subject Areas (b) *	SDG (c) *	Authors (d) *	Affiliation Institutions (e) *	Affiliation Countries/Regions (f) *
Acta Horticulturae (27)	Plant sciences (56)	SDG 3—Good health and well-being (132)	Du Toit E. S. (6)	Egyptian Knowledge Bank (33)	South Africa (66)
South African Journal of Botany (21)	Food science technology (36)	SDG 2—Zero hunger (60)	Ndhlala A. R. (6)	University of Kwazulu Natal (18)	Nigeria (50)
I International Symposium on Moringa (13)	Horticulture (30)	SDG 6—Clean water and sanitation (56)	Abdalla A. M. (4)	University of Pretoria (16)	Egypt (34)
Journal of Ethnopharmacology (9)	Agronomy (24)	SDG 13—Climate action (53)	Bancessi A. (4)	National Research Centre (12)	Ghana (22)
Egyptian Journal of Chemistry (7)	Integrative complementary medicine (22)	SDG 15—Life on land (38)	Catarino L. (4)	University of Limpopo (12)	USA (19)
II International Symposium on Moringa (7)	Pharmacology—pharmacy (21)	SDG 1—No poverty (18)	Ezzo M. I. (4)	CGIAR (10)	Tanzania (15)
Agroforestry Systems (6)	Environmental sciences (20)	SDG 11—Sustainable cities and communities (7)	Khamzina A. (4)	Agricultural Research Council of South Africa (8)	England (14)
Food Science Nutrition (6)	Multidisciplinary sciences (18)	SDG 14—Life below water (4)	Kolanisi U. (4)	Kwame Nkrumah University Science Technology (8)	France (13)
PLOS One (5)	Chemistry medicinal (17)	SDG 12—Responsible consumption and production (3)	Lamers J. P. A. (4)	University of Fort Hare (8)	Germany (11)
Applied Ecology and Environmental Research, BMC Complementary and Alternative Medicine, Molecules (4)	Chemistry multidisciplinary (16)	SDG 5—Gender equality, SDG 7—Affordable and clean energy (1)	Merwad A. R. M. A., Mkandawire T., Ncube B., Noulèkoun F., O’Neill J. G., Pritchard M., Siwela M. (4)	Agricultural Research Center Egypt, Cairo University, North West University South Africa (7)	Kenya (11)

* Figures in brackets refer to the number of publications by journal/source (a), subject area (b), SDG (c), author (d), affiliation (e), or country (f).

**Table 5:** Food chain stages.

Food Chain Stage *	Articles
Production	Abdalla et al. [153]; Abdelwanis et al. [157]; Abdelwanise et al. [158]; Abotsi et al. [385]; Adeyemo [262]; Adhikari et al. [318]; Adu-Dapaah et al. [202]; Akanbi et al. [268]; Anele et al. [272]; Arigbede et al. [275]; Bartíková et al. [190]; Bopape-Mabapa et al. [320]; Boumenjel et al. [389]; Chang et al. [227]; Chitiyo et al. [321]; Darwish et al. [159]; Diouf et al. [309]; du Toit et al. [322]; du Toit et al. [323]; du Toit et al. [324]; El-Boraie et al. [161]; Etongo et al. [131]; Ezzo et al. [164]; Fagbenro et al. [283]; Fare et al. [386]; Gandji et al. [118]; Gandji et al. [119]; Halilou et al. [255]; Ibrahim et al. [170]; Ibrahim et al. [327]; Krieg et al. [132]; Kudzinawo et al. [212]; Kumssa et al. [105]; Mabapa et al. [330]; Managa et al. [332]; Manduwa et al. [333]; Marzougui et al. [392]; Melesse et al. [197]; Mokgehle et al. [338]; Motis et al. [339]; Munyanziza and Sarwatt [381]; Nduwayezu et al. [125]; Němec et al. [198]; Nofal et al. [182]; Noulèkoun et al. [120]; Noulèkoun et al. [121]; Noulèkoun et al. [122]; Noulèkoun et al. [123]; Osman and El-Naggar [183]; Palada et al. [407]; Popoola and Obembe [303]; Ratnadass et al. [256]; Ratshilivha et al. [355]; Ratshilivha et al. [356]; Rébufa et al. [138]; Sagona et al. [245]; Sharaf-Eldin et al. [363]; Swanepoel [364]; Tetteh et al. [222]; Tshabalala et al. [367]; Tshabalala et al. [368]; Waterman et al. [232]; Yamato et al. [404]; Yannick et al. [141]; Zeru et al. [369]; Zubuko et al. [188]
Processing	Abdelsayed et al. [156]; Abdelwanis et al. [157]; Adeyemo [262]; Adu-Dapaah et al. [202]; Amagloh et al. [205]; Aviara et al. [278]; Bassey et al. [319]; Bennour et al. [387]; Bennour et al. [388]; Bombo et al. [124]; Boukandoul et al. [108]; Braham et al. [110]; du Toit et al. [323]; Ebiloma et al. [281]; Fare et al. [386]; Gharsallah et al. [390]; Hamada et al. [165]; Ibrahim et al. [327]; Iwuji et al. [287]; Jimoh et al. [288]; Kaggwa et al. [394]; Kibazohi and Sangwan [377]; Kivevele and Huan [328]; Kudzinawo et al. [212]; Manuwa et al. [292]; Marrufo et al. [251]; Mohamed et al. [176]; Oladeji et al. [299]; Ouahrani et al. [112]; Ralepele et al. [353]
Marketing/commercialization and distribution/retail	Bancessi et al. [223]; Obayelu et al. [294]; Ojo et al. [297]; Seifu and Teketay [127]; Tafesse et al. [199]
Consumption	Abasse et al. [252]; Abrogoua et al. [147]; Abubakar et al. [259]; Adefa and Tefera [189]; Adetola et al. [316]; Adhikari et al. [317]; Adu-Dapaah et al. [202]; Aduro and Ebenso [263]; Affonfere et al. [114]; Agbagwa et al. [264]; Agoyi et al. [115]; Alphonce et al. [373]; Amaeze et al. [269]; Amaeze et al. [270]; Amaeze et al. [271]; Amaglo et al. [203]; Anjorin et al. [273]; Aprioku et al. [274]; Arsene et al. [135]; Atawodi et al. [276]; Attah et al. [277]; Awoyale et al. [233]; Bancessi et al. [223]; Bancessi et al. [224]; Barminas et al. [280]; Bassey et al. [319]; Bellostas et al. [253]; Bisanz et al. [375]; Boateng et al. [206]; Boateng et al. [207]; Bopape-Mabapa et al. [320]; Chadha and Olouch [401]; Chadha et al. [402]; de Saint Sauveur et al. [106]; Debela and Tolera [191]; Dièye et al. [308]; Doglikuu et al. [210]; Dougnon and Ito [117]; Ebert and Palada [405]; Ezaldeen et al. [372]; Fernandes et al. [225]; Fernandes et al. [226]; Freiberger et al. [254]; Gambo et al. [284]; Gambo et al. [286]; Gandji et al. [118]; Gebrezgi [193]; Gharsallah et al. [390]; Glover-Amengor et al. [211]; Govender and Naicker [325]; Gurmu et al. [194]; Hedhili et al. [326]; Hekmat et al. [376]; James et al. [314]; Jilcott et al. [393]; Kaggwa et al. [394]; Kamau et al. [228]; Kasolo et al. [395]; Kumssa et al. [400]; Kumssa et al. [105]; Lawal et al. [289]; Leone et al. [146]; Lockett et al. [290]; Lubaale et al. [329]; Lunyera et al. [378]; Mahomoodally and Ramalingum [248]; Marobhe and Sabai [379]; Maroyi [398]; Mathieu and Meissa [312]; Mawouma et al. [136]; Mekuria et al. [196]; Mudau et al. [341]; Munyanziza and Sarwatt [381]; Mushagalusa Kasali et al. [151]; Mwamatope et al. [239]; Mwamatope et al. [240]; Namla et al. [293]; Ndhlala et al. [344]; Neergheen-Bhujun et al. [249]; Netshiheni et al. [345]; Ntila et al. [347]; Ntila et al. [348]; Ntshambiwa et al. [126]; Obayelu et al. [294]; Oladeji et al. [300]; Olaoye et al. [301]; Olusanya et al. [349]; Oyekale [302]; Pakade et al. [351]; Pakade et al. [352]; Peprah et al. [217]; Popoola and Obembe [303]; Rakotosamimanana et al. [235]; Ranasinghe et al. [315]; Rubio [236]; Saha et al. [139]; Semenya and Maroyi [360]; Semenya and Maroyi [361]; Semenya et al. [362]; Sena et al. [257]; Shija et al. [384]; Sibeko and Johns [107]; Smith et al. [399]; Stevens et al. [304]; Tabong et al. [221]; Tafesse et al. [200]; Tiloke et al. [365]; Tiloke et al. [366]; Tshingani et al. [152]; Vunain et al. [246]; Yongabi et al. [142]; Zubuko et al. [188]; Zungu et al. [370]

* Several documents addressed different stages of the food chain.

**Table 6:** Agronomic aspects addressed in articles dealing with the production of moringa.

Agronomic Practice *	Articles
Fertilization and soil fertility management	Abdelwanis et al. [157]; Abdelwanise et al. [158]; Adu-Dapaah et al. [202]; Akanbi et al. [268]; Darwish et al. [159]; Fagbenro et al. [283]; Fagbenro et al. [283]; Kumssa et al. [105]; Noulèkoun et al. [123]; Ratshilivha et al. [355]; Zubuko et al. [188]
Pest management	Bartíková et al. [190]; Halilou et al. [255]; Kumssa et al. [105]; Němec et al. [198]; Ratnadass et al. [256]; Zeru et al. [369]
Irrigation	El-Boraie et al. [161]; Ezzo et al. [164]; Noulèkoun et al. [121]; Noulèkoun et al. [123]; Osman and El-Naggar [183]

* Some documents addressed different agronomic practices.

**Table 7:** Use of moringa in fertilization in Africa.

Document	Country	Crop/Plant	Use of Moringa
Mosa et al. [180]	Egypt	Apple	Foliar application (biostimulant)
Sarhan et al. [185]	Egypt	Gladiolus (*Gladiolus grandifloras*)	Soil fertilizer
El-Hadidy et al. [162]	Egypt	Orange	Soil fertilizer (moringa seed cake)
Ibrahim and Namich [169]	Egypt	Cotton	Foliar application (biostimulant)
Aba et al. [258]	Nigeria	Plantain	Cover crop/alley cropping
Nofal et al. [181]	Egypt	African marigold (*Tagetes erecta*)	Foliar application
Agbede et al. [266]	Nigeria	Tomato	Green manure
Ibrahim et al. [170]	Egypt	Citrus, grape, olive, loquat, and persimmon	Soil amendment
Hemdan et al. [167]	Egypt	Orange	Soil amendment
Thabet et al. [187]	Egypt	Date palm	Foliar application
Ibrahim and Namich [169]	Egypt	Cotton	Foliar application
Ngcobo and Bertling [346]	South Africa	Cherry tomato	Foliar application
Masete et al. [334]	South Africa	Cowpea	Mulch
Merwad [172]	Egypt	Pea	Foliar application

**Table 8:** Articles linking moringa with climate and ecosystem resilience in Africa.

Theme *	Documents
Climate change and variability	Abdalla et al. [153]; Anele et al. [272]; Arigbede et al. [275]; Bombo et al. [124]; Boumenjel et al. [389]; Chitiyo et al. [321]; Debela and Tolera [191]; Ezzo et al. [164]; Ibrahim et al. [327]; Kibazohi and Sangwan [377]; Kivevele and Huan [328]; Mabapa et al. [330]; Marzougui et al. [392]; Meale et al. [406]; Melesse et al. [197]; Moussavou et al. [134]; Munyanziza and Sarwatt [381]; Noulèkoun et al. [120]; Seifu and Teketay [127]; Yamato et al. [404]; Zeru et al. [369]
Land degradation and desertification	Abdel Moniem et al. [155]; Desoky et al. [160]; Gandji et al. [119]; Ibrahim and Namich [169]; Merwad [173]; Merwad [174]; Nofal et al. [182]; Noulèkoun et al. [120]; Noulèkoun et al. [121]; Noulèkoun et al. [122]; Noulèkoun et al. [123]; Osman and El-Naggar [183]
Water and soil pollution	Abdel Moniem et al. [155]; Acheampong et al. [201]; Adhikari et al. [318]; Boulaadjoul et al. [109]; Gold et al. [311]; Kebaili et al. [111]; Kilingo et al. [229]; Marzougui et al. [391]; Ngounouno et al. [137]; Ntibrey et al. [215]; Pritchard et al. [243]; Senanu et al. [219]; Sengupta et al. [220]; Tchangoue et al. [140]; Vunain et al. [246]

* Some documents addressed different themes.

**Table 9:** Health claims related to moringa.

Document	Country	Concerned Disease(s)/Pharmacological Properties	Source of Data
Abubakar et al. [259]	Nigeria	Viral diseases (including hepatitis, poliomyelitis, monkeypox, smallpox, yellow fever, Lassa fever, meningitis, and COVID-19)	Survey (questionnaires and interviews)
Adeyemi et al. [261]	Nigeria	Anti-hemorrhagic activity	Laboratory test
Agoyi et al. [115]	Benin	Different diseases including venereal, cardiovascular, infectious, tropical, and digestive ones	Ethnobotanical survey
Ajuogu et al. [267]	Nigeria	Reproductive hormones and semen quality	Laboratory test
Ali et al. [371]	Sudan	Malaria and other tropical diseases	In vitro test
Amaeze et al. [269]	Nigeria	Different diseases	In vitro test
Amaeze et al. [270]	Nigeria	Different diseases	In vitro test
Amaeze et al. [271]	Nigeria	Type 2 diabetes mellitus	Cross-sectional survey
Atawodi et al. [276]	Nigeria	Anti-cancer activity	In vitro test
Dièye et al. [308]	Senegal	Diabetes	Cross-sectional survey
Doglikuu et al. [210]	Ghana	Type 2 diabetes mellitus (T2DM)	Cross-sectional survey
Ejeta et al. [192]	Ethiopia	Mosquito control (cf. malaria)	Survey Laboratory test
James et al. [314]	Sierra Leone	Hypertension Cardiovascular diseases	Cross-sectional survey
Kamau et al. [228]	Kenya	Diabetes	Cross-sectional survey
Lunyera et al. [378]	Tanzania	Diabetes mellitus	Survey (focus groups and semi-structured interviews)
Maiyo et al. [331]	South Africa	Cancers	In vitro test
Mekuria et al. [196]	Ethiopia	Type 2 diabetes	Cross-sectional survey
Mudau et al. [341]	South Africa	Diabetes	Ethnobotanical survey
Mushagalusa Kasali et al. [151]	DRC	Hypertension	Ethnopharmacological survey
Mwamatope et al. [240]	Malawi	Cancer	Ethnobotanical survey
Oladeji et al. [300]	Nigeria	Malaria	Survey (structured questionnaire)
Omodanisi et al. [350]	South Africa	Anti-hyperglycaemic, anti-inflammatory, and antioxidant activities	Laboratory test
Popoola and Obembe [303]	Nigeria	Different diseases	Ethnobotanical survey
Ranasinghe et al. [315]	Sierra Leone	Malaria	Ethnopharmacological survey
Salama et al. [184]	Egypt	Liver injury/hepatoprotective effects	Laboratory test
Semenya and Maroyi [360]	South Africa	Sinusitis	Survey (semi-structured interviews)
Tabong et al. [221]	Ghana	Diabetes mellitus	Focus group
Tiloke et al. [365]	South Africa	Lung cancer	In vitro test
Tiloke et al. [366]	South Africa	Oesophageal cancer	In vitro test

## Data Availability

Data are contained within the article.

## Appendix A

**Table A1:** Agriculture and Food Security in Africa.

Country	Agriculture, Forestry, and Fishing, Value Added (% of GDP) (Value (Year))	Employment in Agriculture (% of Total Employment) (2021)	Prevalence of Undernourishment (% of Population) (2020–2022)	Prevalence of Moderate or Severe Food Insecurity (% of Population) (2020–2022)
Algeria	11.6 (2022)	10	19.3	58.9
Angola	13.6 (2022)	59	21.6	78.5
Benin	26.9 (2022)	28	9.9	73.6
Botswana	1.8 (2022)	23	22.9	56.3
Burkina Faso	18.5 (2022)	73	16.2	56.9
Burundi	27.6 (2022)	86	n.a.	n.r.
Cabo Verde/Cape Verde	4.8 (2022)	11	18.2	37.0
Cameroon	17.0 (2022)	43	6.4	58.5
Central African Republic	36.2 (2022)	69	48.7	81.3
Chad	22.6 (2022)	69	31.4	n.r.
Comoros	36.4 (2022)	35	13.5	79.7
Congo	8.0 (2022)	36	33.3	88.2
Côte d’Ivoire/Ivory Coast	16.7 (2022)	45	7.7	44.2
Democratic Republic of the Congo (DRC)	17.4 (2022)	55	35.3	76.6
Djibouti	1.8 (2022)	1	16.8	49.2
Egypt	10.9 (2022)	20	7.2	28.5
Equatorial Guinea	2.7 (2022)	56	n.a.	n.a.
Eritrea	14.1 (2009)	62	n.a.	n.a.
Eswatini/Swaziland	8.6 (2022)	12	11.6	67.0
Ethiopia	37.6 (2022)	64	21.9	58.1
Gabon	5.6 (2022)	29	23.0	n.r.
Ghana	19.6 (2022)	39	4.9	39.4
Guinea	27.3 (2022)	59	12.9	73.1
Guinea-Bissau	30.9 (2020)	50	37.9	77.8
Kenya	21.2 (2022)	33	27.8	72.3
Lesotho	6.2 (2022)	30	46.0	56.7
Liberia	36.2 (2022)	41	38.4	81.2
Libya	1.6 (2022)	16	8.4	39.8
Madagascar	21.9 (2022)	74	51.0	64.9
Malawi	21.8 (2022)	62	17.8	82.4
Mali	36.4 (2022)	68	12.8	n.r.
Mauritania	22.2 (2022)	29	8.7	53.7
Mauritius	3.6 (2022)	5	6.8	32.0
Morocco	10.3 (2022)	35	6.3	n.r.
Mozambique	26.7 (2022)	70	30.5	75.4
Namibia	8.4 (2022)	22	17.1	57.7
Niger	42.0 (2022)	71	16.1	71.4
Nigeria	23.7 (2022)	35	15.9	69.7
Rwanda	24.9 (2022)	55	31.6	n.r.
São Tomé and Príncipe	13.9 (2022)	18	13.1	54.6
Senegal	15.5 (2022)	22	5.7	49.8
Seychelles	2.9 (2022)	n.a.	4.3	14.7
Sierra Leone	60.4 (2022)	43	27.8	89.2
Somalia	62.7 (1990)	26	48.7	79.5
South Africa	2.8 (2022)	21	7.9	20.3
South Sudan	10.4 (2015)	62	21.4	87.3
Sudan	5.0 (2022)	41	11.9	51.8
Tanzania	24.3 (2022)	64	23.5	58.7
The Gambia	22.6 (2022)	49	19.6	60.7
Togo	18.3 (2022)	31	17.4	62.9
Tunisia	9.8 (2022)	14	3.0	28.5
Uganda	24.0 (2022)	63	31.6	74.2
Zambia	3.1 (2022)	59	29.8	73.1
Zimbabwe	7.2 (2022)	62	38.4	73.6
World	4.3 (2022)	26	9.2	29.5
Source	World Bank [1]	World Bank [2]	FAO et al. [3]	FAO et al. [3]

GDP: gross domestic product; n.r. not reported; n.a. not available.

## Appendix B

**Table A2:** Previous Reviews Dealing with Moringa in Africa.

Review	Publication Date	Review Type	Geographical Coverage	Thematic Focus
Adetola et al. [78]	February 2023	Narrative review	Africa	Bioavailability of iron and zinc in diets
Frazzoli et al. [79]	January 2023	Narrative review	Africa	Immune system and epidemics
Mbokane and Moyo [67]	December 2022	Narrative review	Southern Africa	Use of medicinal plants as feed additives in fish diets
Horn et al. [50]	October 2022	Narrative review	Africa	Nutrition Disease prevention Water treatment
Usai et al. [51]	August 2022	Narrative review	Zimbabwe	Diabetes mellitus
Golla et al. [80]	August 2022	Meta-analysis	Global	Properties of moringa pods
Ichrak [81]	August 2022	Narrative review	North Africa	Anaphylaxis caused by moringa
Saadat et al. [82]	May 2022	Narrative review	Global	Lung disorders
Odukoya et al. [52]	May 2022	Narrative review	Sub-Saharan Africa	Cardiovascular diseases
Alia et al. [53]	January 2022	Narrative review	Global	Cardiac damage and vascular dysfunction
Liu et al. [54]	October 2021	Systematic review	Global	Botany Traditional uses Phytochemistry, pharmacology, and toxicity
Jain et al. [83]	July 2021	Narrative review	Global	Biotechnology Pharmacological properties
Losso et al. [84]	June 2021	Narrative review	Sub-Saharan Africa	Plant-based diet and COVID-19
George et al. [85]	May 2021	Bibliometric analysis	Global	Bibliometrics
Peter et al. [55]	April 2021	Narrative review	Tanzania	Diabetes mellitus
Conti et al. [70]	April 2021	Narrative review	Madagascar	Food insecurity and malnutrition
Adeleye et al. [56]	March 2021	Narrative review	Africa	Ethnomedicinal herbs
Mashamaite et al. [86]	March 2021	Narrative review	South Africa	Agronomy Consumption
Moyo and Rapatsa [87]	January 2021	Narrative review	Southern Africa	Tilapia aquaculture
du Toit [88]	2021	Narrative review	South Africa	Horticultural research
Gariba et al. [89]	January 2021	Narrative review	Ghana	Pest management on maize
Teye et al. [41]	December 2020	Narrative review	Africa	Food fortification
Xiao et al. [63]	December 2020	Narrative review	Global	Immune disorders
Aumeeruddy and Mahomoodally [57]	December 2020	Narrative review	Global	Hypertension
Mollel et al. [90]	March 2020	Narrative review	Global	Health and nutrition Industrial applications
Omara et al. [58]	March 2020	Narrative review	Uganda	Cancer
Mashamaite et al. [91]	March 2020	Narrative review	South Africa	Ecology Social costs and benefits Legal status
Padayachee and Baijnath [59]	March 2020	Narrative review	Global	Medicinal, pharmacological, and phytochemical properties
Singh et al. [60]	March 2020	Narrative review	Global	Phytochemical and pharmacological attributes
Muzumbukilwa et al. [92]	August 2019	Systematic review	Global	Hepatoprotective properties
Abd Rani et al. [64]	February 2018	Narrative review	Global	Phytochemistry and pharmacology
Tamilselvi and Arumugam [35]	2018	Narrative review	Global	Health benefits Therapeutic and pharmacological properties
Vergara-Jimenez et al. [93]	December 2017	Narrative review	Global	Protection against chronic diseases
Domenech Asensi et al. [42]	June 2017	Narrative review	Global	Food applications
Amaglo et al. [71]	2017	Narrative review	Global	Climate change Livelihoods Food security
Keatinge et al. [72]	2017	Narrative review	Global	Nutrition Food security
Luoh et al. [37]	2017	Narrative review	Global	Phytonutrient values of leaves
Lyons et al. [38]	2017	Narrative review	Global	Nutritional profile of leaves
Olson [94]	2017	Narrative review	Global	General overview of moringa
Palada et al. [95]	2017	Narrative review	Global	Research on moringa
Pasternak et al. [96]	2017	Narrative review	Niger	Moringa research and cultivation
Fokou et al. [97]	2017	Narrative review	West Africa (Ivory Coast, Ghana, and Benin)	Medicinal plants against Buruli ulcer
Thelingwani and Masimirembwa [98]	2014	Narrative review	Global	Herbal medicines
Shahzad et al. [99]	December 2013	Narrative review	Global	Genetic diversity
Alao et al. [100]	December 2011	Narrative review	Nigeria	Pest management
Naidoo and Coopoosamy [61]	August 2011	Narrative review	South African region	Herbal remedies
McBurney et al. [101]	June 2004	Narrative review	Africa	Nutritional composition

## Appendix C

**Table A3:** Selected Articles Dealing with Research on *M. oleifera* in Africa.

Year	Number of Articles	References
2023 *	6	Lawal et al. [289]; Lubaale et al. [329]; Mohamed et al. [178]; Naidoo [343]; Ntshambiwa et al. [126]; Sarkwa et al. [218]
2022	36	Abdalla et al. [153]; Abdelwanis et al. [157]; Abubakar et al. [259]; Adhikari et al. [318]; Agbagwa et al. [264]; Aprioku et al. [274]; Bancessi et al. [224]; Bassey et al. [319]; Gambo et al. [284]; Halilou et al. [255]; Hedhili et al. [326]; Ibrahim et al. [170]; Ibrahim et al. [327]; Jambwa et al. [397]; Kaggwa et al. [394]; Kebaili et al. [111]; Kilingo et al. [229]; Kudzinawo et al. [212]; Masete et al. [334]; Mhlomi et al. [335]; Mokgehle et al. [338]; Mosa et al. [180]; Mudau et al. [341]; Mwamatope et al. [240]; Namla et al. [293]; Ojo et al. [297]; Ondiba et al. [403]; Osman and El-Naggar [183]; Ouahrani et al. [112]; Peprah et al. [217]; Saha et al. [139]; Semanka et al. [128]; Smith et al. [399]; Thabet et al. [187]; Yannick et al. [141]; Zeru et al. [369]
2021	56	Aba et al. [258]; Abdel Moniem et al. [155]; Abdelsayed et al. [156]; Adeyemi et al. [261]; Adhikari et al. [317]; Affonfere et al. [114]; Alphonce et al. [374]; Amaeze et al. [269]; Amaeze et al. [270]; Arsene et al. [135]; Bennour et al. [388]; Bombo et al. [124]; Bopape-Mabapa et al. [320]; Boumenjel et al. [389]; Chitiyo et al. [321]; Choga et al. [396]; Cumbe et al. [250]; Darwish et al. [159]; Doglikuu et al. [210]; Ejeta et al. [192]; El-Hadidy et al. [162]; Fernandes et al. [225]; Fernandes et al. [226]; Gambo et al. [285]; Gambo et al. [286]; Gharsallah et al. [390]; Habtemariam et al. [195]; Hamada et al. [165]; Hemdan et al. [167]; Ibrahim and Namich [169]; Iwuji et al. [287]; Managa et al. [332]; Marobhe and Sabai [379]; Marzougui et al. [391]; Marzougui et al. [392]; Mohamed et al. [176]; Moichela et al. [337]; Mushagalusa Kasali et al. [151]; Ngcobo and Bertling [346]; Ngounouno et al. [137]; Nofal et al. [181]; Nofal et al. [182]; Olaoye et al. [301]; Ralepele et al. [353]; Ratshilivha et al. [356]; Rébufa et al. [138]; Rikhotso et al. [358]; Sarhan et al. [185]; Senanu et al. [219]; Sibeko and Johns [107]; Stevens et al. [304]; Swanepoel [364]; Tetteh et al. [222]; Tshabalala et al. [368]; Ugese and Iordye [307]; Waterman et al. [232]
2020	41	Adefa and Tefera [189]; Adéyèmi et al. [113]; Attah et al. [277]; Bancessi et al. [223]; Bartíková et al. [190]; Bennour et al. [387]; Braham et al. [110]; Cudjoe et al. [209]; Dougnon and Ito [117]; du Toit et al. [323]; du Toit et al. [324]; El-Boraie et al. [161]; El-Helaly [163]; Gandji et al. [119]; Govender and Naicker [325]; Jimoh et al. [288]; Manuwa et al. [292]; Mao et al. [214]; Merwad [173]; Mmanda et al. [380]; Mohamed et al. [177]; Monir et al. [179]; Mwamatope et al. [239]; Neergheen-Bhujun et al. [249]; Němec et al. [198]; Ntibrey et al. [215]; Ntila et al. [347]; Ochire-Boadu et al. [216]; Okoya et al. [298]; Oladeji et al. [299]; Oladeji et al. [300]; Olusanya et al. [349]; Sagona et al. [245]; Seifu and Teketay [127]; Syeda and Riazunnisa [237]; Tafesse et al. [199]; Tafesse et al. [200]; Taiwo et al. [305]; Tshabalala et al. [367]; Zeitoun et al. [313]; Zungu et al. [370]
2019	25	Abdallah et al. [154]; Adetola et al. [316]; Aduro and Ebenso [263]; Agbede et al. [265]; Agbede et al. [266]; Ajuogu et al. [267]; Akanbi et al. [268]; Alphonce et al. [373]; Boateng et al. [207]; Chang et al. [227]; Desoky et al. [160]; Gebrezgi [193]; Mahklouf [234]; Mansour et al. [171]; Melesse et al. [197]; Modisaojang-Mojanaga et al. [336]; Netshiheni et al. [345]; Ntila et al. [348]; Rikhotso et al. [357]; Rubio [236]; Shaaban et al. [186]; Shija et al. [384]; Tchangoue et al. [140]; Vunain et al. [246]; Zubuko et al. [188]
2018	21	Amaeze et al. [271]; Awoyale et al. [233]; Bachabi et al. [116]; Boateng et al. [206]; Boulaadjoul et al. [109]; Clark et al. [208]; Ezzo et al. [164]; Gandji et al. [118]; Hegazi et al. [166]; James et al. [314]; Mabapa et al. [330]; Manduwa et al. [333]; Mekuria et al. [196]; Merwad [172]; Noulèkoun et al. [120]; Noulèkoun et al. [122]; Ogundiran et al. [296]; Salama et al. [184]; Semenya and Maroyi [360]; Semenya and Maroyi [361]; Tabong et al. [221]
2017	31	Abdelwanise et al. [158]; Abotsi et al. [385]; Adeyemo [262]; Adu-Dapaah et al. [202]; Agoyi et al. [115]; Amagloh et al. [205]; Amante-García et al. [130]; Boukandoul et al. [108]; de Saint Sauveur et al. [106]; du Toit et al. [322]; Ebert and Palada [405]; Ebiloma et al. [281]; Fare et al. [386]; Glover-Amengor et al. [211]; Gurmu et al. [194]; Ibrahim and Abdalla [168]; Kampemba et al. [150]; Kpan et al. [148]; Krieg et al. [132]; Kumssa et al. [400]; Kumssa et al. [105]; Mawouma et al. [136]; Merwad [174]; Merwad and Abdel-Fattah [175]; Motis et al. [339]; Noulèkoun et al. [121]; Noulèkoun et al. [123]; Omodanisi et al. [350]; Ratshilivha et al. [355]; Sharaf-Eldin et al. [363]; Tshingani et al. [152]
2016	8	Amante et al. [129]; Gold et al. [311]; Kamau et al. [228]; Lunyera et al. [378]; Maiyo et al. [331]; Moussavou et al. [134]; Muhl et al. [342]; Tiloke et al. [366]
2015	13	Abubakar et al. [260]; Aviara et al. [278]; Bisanz et al. [375]; Etongo et al. [131]; Fagbenro et al. [283]; Hekmat et al. [376]; Kivevele and Huan [328]; Leone et al. [146]; Mahomoodally and Ramalingum [248]; Obayelu et al. [294]; Rakotosamimanana et al. [235]; Ranasinghe et al. [315]; Sebola et al. [359]
2014	3	Kambashi et al. [149]; Ndhlala et al. [344]; Rapatsa and Moyo [354]
2013	11	Debela and Tolera [191]; Ezaldeen et al. [372]; Kumah et al. [213]; Lopez-Grimau et al. [133]; Maroyi [398]; Marrufo et al. [251]; Pakade et al. [351]; Pakade et al. [352]; Popoola and Obembe [303]; Tiloke et al. [365]; Titiloye et al. [306]
2012	8	Abrogoua et al. [147]; Arigbede et al. [275]; Mahdy et al. [291]; Moyo et al. [340]; Oyekale [302]; Poumaye et al. [145]; Semenya et al. [362]; Sengupta et al. [220]
2011	9	Acheampong et al. [201]; Ayotunde et al. [279]; Kibazohi and Sangwan [377]; Meale et al. [406]; Ratnadass et al. [256]; Walter et al. [231]; Yongabi et al. [143]; Yongabi et al. [144]
2010	8	Amaglo et al. [203]; Anjorin et al. [273]; Atawodi et al. [276]; Bellostas et al. [253]; Jilcott et al. [393]; Kasolo et al. [395]; Pritchard et al. [241]; Pritchard et al. [242]
2009	7	Amagloh and Benang [204]; Etuk and Mohammed [282]; Ogunbinu et al. [295]; Pritchard et al. [243]; Pritchard et al. [244]; Yamato et al. [404]; Yongabi et al. [142]
2008	2	Anele et al. [272]; Dièye et al. [308]
2007	6	Abasse et al. [252]; Chadha and Olouch [401]; Chadha et al. [402]; Diouf et al. [309]; Mathieu and Meissa [312]; Nduwayezu et al. [125]
2006	1	Ndemanisho et al. [382]
2003	1	Munyanziza and Sarwatt [381]
2002	3	Ali et al. [371]; Palada et al. [407]; Sarwatt et al. [383]
2000	1	Lockett et al. [290]
1999	2	McConnachie et al. [238]; Muluvi et al. [230]
1998	3	Barminas et al. [280]; Freiberger et al. [254]; Sena et al. [257]
1996	1	Warhurst et al. [247]
1995	1	Nwosu and Okafor [104]

* As of 7 April 2023.

## Appendix D

**Table A4:** Parts of Moringa Addressed in the Selected Articles.

Moringa Part *	Documents
Flowers	du Toit et al. [324]; Ebert and Palada [405]; Fernandes et al. [225]; Krieg et al. [132]; Manduwa et al. [333]; Pakade et al. [351]; Pakade et al. [352]
Fruits/pods/drumsticks	Abdelwanise et al. [158]; Abotsi et al. [385]; Anjorin et al. [273]; Debela and Tolera [191]; du Toit et al. [324]; Ebert and Palada [405]; Gandji et al. [119]; Krieg et al. [132]; Kumssa et al. [400]; Mahomoodally and Ramalingum [248]; Manduwa et al. [333]; Melesse et al. [197]; Neergheen-Bhujun et al. [249]; Sagona et al. [245]; Seifu and Teketay [127]
Leaves	Abdalla et al. [153]; Abdel Moniem et al. [155]; Abdelsayed et al. [156]; Abdelwanis et al. [157]; Abdelwanise et al. [158]; Abotsi et al. [385]; Abrogoua et al. [147]; Abubakar et al. [259]; Abubakar et al. [260]; Adefa and Tefera [189]; Adetola et al. [316]; Adéyèmi et al. [113]; Adeyemi et al. [261]; Adhikari et al. [317]; Adu-Dapaah et al. [202]; Affonfere et al. [114]; Agbede et al. [266]; Agoyi et al. [115]; Ajuogu et al. [267]; Alphonce et al. [373]; Alphonce et al. [374]; Amaeze et al. [270]; Amaglo et al. [203]; Anele et al. [272]; Anjorin et al. [273]; Aprioku et al. [274]; Arigbede et al. [275]; Arsene et al. [135]; Atawodi et al. [276]; Attah et al. [277]; Bachabi et al. [116]; Bancessi et al. [223]; Barminas et al. [280]; Bellostas et al. [253]; Bennour et al. [387]; Bennour et al. [388]; Boateng et al. [206]; Boateng et al. [207]; Bopape-Mabapa et al. [320]; Boumenjel et al. [389]; Braham et al. [110]; Clark et al. [208]; de Saint Sauveur et al. [106]; Debela and Tolera [191]; Desoky et al. [160]; du Toit et al. [323]; du Toit et al. [324]; Ebert and Palada [405]; Ebiloma et al. [281]; Ejeta et al. [192]; El-Boraie et al. [161]; Fare et al. [386]; Fernandes et al. [225]; Fernandes et al. [226]; Freiberger et al. [254]; Gambo et al. [284]; Gambo et al. [285]; Gambo et al. [286]; Gandji et al. [118]; Gandji et al. [119]; Gebrezgi [193]; Glover-Amengor et al. [211]; Hamada et al. [165]; Hedhili et al. [326]; Hegazi et al. [166]; Ibrahim and Namich [169]; Ibrahim et al. [170]; Ibrahim et al. [170]; Jilcott et al. [393]; Kasolo et al. [395]; Kumah et al. [213]; Kumssa et al. [400]; Kumssa et al. [105]; Leone et al. [146]; Lubaale et al. [329]; Mahdy et al. [291]; Mahomoodally and Ramalingum [248]; Maiyo et al. [331]; Managa et al. [332]; Mansour et al. [171]; Manuwa et al. [292]; Marrufo et al. [251]; Masete et al. [334]; Mawouma et al. [136]; Melesse et al. [197]; Merwad [172]; Merwad [173]; Merwad [174]; Merwad and Abdel-Fattah [175]; Mhlomi et al. [335]; Mmanda et al. [380]; Modisaojang-Mojanaga et al. [336]; Mohamed et al. [176]; Mohamed et al. [177]; Mohamed et al. [178]; Moichela et al. [337]; Mokgehle et al. [338]; Monir et al. [179]; Mosa et al. [180]; Moyo et al. [340]; Mwamatope et al. [239]; Mwamatope et al. [240]; Naidoo [343]; Namla et al. [293]; Neergheen-Bhujun et al. [249]; Němec et al. [198]; Netshiheni et al. [345]; Ngcobo and Bertling [346]; Nofal et al. [181]; Nofal et al. [182]; Ntila et al. [347]; Ntila et al. [348]; Ntshambiwa et al. [126]; Ojo et al. [297]; Oladeji et al. [299]; Olaoye et al. [301]; Olusanya et al. [349]; Ouahrani et al. [112]; Pakade et al. [351]; Pakade et al. [352]; Popoola and Obembe [303]; Ralepele et al. [353]; Ratshilivha et al. [355]; Ratshilivha et al. [356]; Rébufa et al. [138]; Rikhotso et al. [357]; Rikhotso et al. [358]; Saha et al. [139]; Sarhan et al. [185]; Sarwatt et al. [383]; Sebola et al. [359]; Seifu and Teketay [127]; Sena et al. [257]; Senanu et al. [219]; Shija et al. [384]; Smith et al. [399]; Stevens et al. [304]; Syeda and Riazunnisa [237]; Tafesse et al. [200]; Tetteh et al. [222]; Thabet et al. [187]; Tiloke et al. [365]; Tiloke et al. [366]; Tshabalala et al. [368]; Tshingani et al. [152]; Zeru et al. [369]; Zubuko et al. [188]; Zungu et al. [370]
Roots	Agoyi et al. [115]; Amaglo et al. [203]; Atawodi et al. [276]; Seifu and Teketay [127]
Seeds (including oil pressed from seeds and seed cake)	Abdelsayed et al. [156]; Abdelwanis et al. [157]; Abotsi et al. [385]; Acheampong et al. [201]; Aduro and Ebenso [263]; Agbede et al. [265]; Agoyi et al. [115]; Amaglo et al. [203]; Amagloh and Benang [204]; Amante et al. [129]; Amante-García et al. [130]; Anjorin et al. [273]; Aviara et al. [278]; Ayotunde et al. [279]; Bachabi et al. [116]; Bancessi et al. [223]; Bancessi et al. [224]; Bassey et al. [319]; Bombo et al. [124]; Boukandoul et al. [108]; Boulaadjoul et al. [109]; du Toit et al. [322]; Ejeta et al. [192]; El-Hadidy et al. [162]; Fernandes et al. [225]; Gandji et al. [119]; Gharsallah et al. [390]; Gold et al. [311]; Habtemariam et al. [195]; Hemdan et al. [167]; Ibrahim and Abdalla [168]; Jimoh et al. [288]; Kibazohi and Sangwan [377]; Kilingo et al. [229]; Kivevele and Huan [328]; Kumssa et al. [400]; Lopez-Grimau et al. [133]; Maiyo et al. [331]; Marobhe and Sabai [379]; Marzougui et al. [391]; McConnachie et al. [238]; Mohamed et al. [176]; Moussavou et al. [134]; Muhl et al. [342]; Nduwayezu et al. [125]; Ngounouno et al. [137]; Ntibrey et al. [215]; Okoya et al. [298]; Popoola and Obembe [303]; Poumaye et al. [145]; Pritchard et al. [242]; Pritchard et al. [244]; Rapatsa and Moyo [354]; Sagona et al. [245]; Semanka et al. [128]; Sengupta et al. [220]; Shaaban et al. [186]; Sharaf-Eldin et al. [363]; Stevens et al. [304]; Taiwo et al. [305]; Tchangoue et al. [140]; Titiloye et al. [306]; Ugese and Iordye [307]; Vunain et al. [246]; Walter et al. [231]; Warhurst et al. [247]; Yongabi et al. [143]; Yongabi et al. [144]; Zeitoun et al. [313]
Whole plant	Abdalla et al. [153]; Abdallah et al. [154]; Abdelwanise et al. [158]; Abotsi et al. [385]; Adhikari et al. [318]; Adu-Dapaah et al. [202]; Agoyi et al. [115]; Akanbi et al. [268]; Anele et al. [272]; Bancessi et al. [223]; Bartíková et al. [190]; Boumenjel et al. [389]; Chang et al. [227]; Chitiyo et al. [321]; Darwish et al. [159]; Debela and Tolera [191]; du Toit et al. [322]; du Toit et al. [323]; du Toit et al. [324]; El-Boraie et al. [161]; Ezzo et al. [164]; Fagbenro et al. [283]; Fare et al. [386]; Gandji et al. [118]; Gandji et al. [119]; Halilou et al. [255]; Kudzinawo et al. [212]; Kumssa et al. [105]; Mabapa et al. [330]; Mahklouf [234]; Manduwa et al. [333]; Mao et al. [214]; Marzougui et al. [392]; Mokgehle et al. [338]; Motis et al. [339]; Muluvi et al. [230]; Munyanziza and Sarwatt [381]; Mwamatope et al. [240]; Ndhlala et al. [344]; Neergheen-Bhujun et al. [249]; Němec et al. [198]; Noulèkoun et al. [120]; Noulèkoun et al. [121]; Noulèkoun et al. [123]; Ogundiran et al. [296]; Osman and El-Naggar [183]; Ratshilivha et al. [355]; Ratshilivha et al. [356]; Sagona et al. [245]; Seifu and Teketay [127]; Swanepoel [364]; Tafesse et al. [200]; Tetteh et al. [222]; Tshabalala et al. [367]; Tshabalala et al. [368]; Waterman et al. [232]; Yamato et al. [404]; Yannick et al. [141]; Zubuko et al. [188]

* Some documents dealt with different parts of moringa.

## Appendix E

**Table A5:** Articles Dealing with the Use of Moringa in Food Fortification and Supplementation.

Document	Country/Region	Product(s) Fortified	Moringa Product/Part Used	Nutrient(s) Concerned
Adetola et al. [316]	South Africa	Pearl millet porridge	Moringa leaf powder	Iron, calcium, and magnesium
Affonfere et al. [114]	Benin	Maize and sorghum ogi porridges	Moringa leaf powder	Iron (anemia)
Alphonce et al. [373]	Tanzania	Fermented cassava meal “mchuchume”	Moringa leaf powder	Different micronutrients and proteins
Alphonce et al. [374]	Tanzania	Fermented cassava meal “mchuchume”	Moringa leaf powder	Iron, sodium, and beta-carotene
Boateng et al. [206]	Ghana	Different foods	Moringa leaf powder	Provitamin A
Gebrezgi [193]	Ethiopia	Maize and soybean flours	Moringa leaf powder	Proteins, fats, and fibers
Glover-Amengor et al. [211]	Ghana	Different dishes	Moringa leaf powder	Different micronutrients (beta-carotene, Zn, Mn, and Fe)
Glover-Amengor et al. [211]	Ghana	School lunch menu	Moringa leaf powder	Different micronutrients (copper, manganese, iron, zinc, and beta-carotene)
Hekmat et al. [376]	Tanzania	Probiotic yogurt	Moringa leaf powder	Different micronutrients
Lubaale et al. [329]	South Africa	Wholegrain sorghum-based porridges	Moringa leaf powder	Iron
Mawouma et al. [136]	Cameroon	Tamarind pulp-acidulated sauce	Moringa leaf powder	Iron and zinc
Netshiheni et al. [345]	South Africa	Maize porridge	Moringa leaf powder	Different micronutrients (zinc, iron, calcium, and magnesium) and proteins
Ntila et al. [347]	South Africa	White maize soft porridge	Moringa leaf powder	Different micronutrients (Zn, Fe, provitamin A, phenolics, and flavonoids)

## References

[1] World Bank The World Bank Data—Agriculture, Forestry, and Fishing, Value Added (% of GDP). https://data.worldbank.org/indicator/NV.AGR.TOTL.ZS.

[2] World Bank The World Bank Data—Employment in Agriculture (% of Total Employment). https://data.worldbank.org/indicator/SL.AGR.EMPL.ZS.

[3] FAO, IFAD, UNICEF, WFP, WHO (2023). The State of Food Security and Nutrition in the World 2023.

[4] Lokonon B.O.K., Egbendewe A.Y.G., Coulibaly N., Atewamba C. (2019). The Potential Impact of Climate Change on Agriculture in West Africa: A Bio-Economic Modeling Approach. Clim. Chang. Econ..

[5] Bakshi B., Nawrotzki R.J., Donato J.R., Lelis L.S. (2019). Exploring the Link between Climate Variability and Mortality in Sub-Saharan Africa. Int. J. Environ. Sustain. Dev..

[6] Hassan R.M. (2010). The Double Challenge of Adapting to Climate Change While Accelerating Development in Sub-Saharan Africa. Environ. Dev. Econ..

[7] Baarsch F., Granadillos J.R., Hare W., Knaus M., Krapp M., Schaeffer M., Lotze-Campen H. (2020). The Impact of Climate Change on Incomes and Convergence in Africa. World Dev..

[8] Sultan B., Gaetani M. (2016). Agriculture in West Africa in the Twenty-First Century: Climate Change and Impacts Scenarios, and Potential for Adaptation. Front. Plant Sci..

[9] El Bilali H. (2021). Climate Change and Agriculture in Burkina Faso. J. Arid. Agric..

[10] El Bilali H., Cardone G., Rokka S., De Falcis E., Naino Jika A.K., Diawara A.B., Nouhou B., Ghione A. (2023). Sustainability Transitions in West African Agriculture and Food Systems. J. Surv. Fish. Sci..

[11] El Bilali H., Cardone G., Rokka S., De Falcis E., Naino Jika A., Diawara A.B., Nouhou B., Ghione A. Orphan Crops and Sustainability Transitions in Agri-Food Systems: Towards a Multidimensional and Multilevel Transition Framework. Proceedings of the International Conference on Life Sciences (ICLISC-23).

[12] Li X., Siddique K.H.M. (2018). Future Smart Food—Rediscovering Hidden Treasures of Neglected and Underutilized Species for Zero Hunger in Asia.

[13] Padulosi S. (2017). Bring NUS back on the table!. GREAT Insights Magazine.

[14] Padulosi S., Thompson J., Rudebjer P. (2013). Fighting Poverty, Hunger and Malnutrition with Neglected and Underutilized Species (NUS): Needs, Challenges and the Way Forward.

[15] Garn S.M., Leonard W.R. (2009). What Did Our Ancestors Eat?. Nutr. Rev..

[16] Kunkel G. (1984). Plants for Human Consumption.

[17] Wiersema J., Leon B. (1999). World Economic Plants: A Standard Reference.

[18] FAO (1998). The State of the World’s Plant Genetic Resources for Food and Agriculture.

[19] FAO (1995). Dimensions of Need: An Atlas of Food and Agriculture.

[20] Prescott-Allen R., Prescott-Allen C. (1990). How Many Plants Feed the World?. Conserv. Biol..

[21] Chivenge P., Mabhaudhi T., Modi A., Mafongoya P. (2015). The Potential Role of Neglected and Underutilised Crop Species as Future Crops under Water Scarce Conditions in Sub-Saharan Africa. Int. J. Environ. Res. Public Health.

[22] Baldermann S., Blagojević L., Frede K., Klopsch R., Neugart S., Neumann A., Ngwene B., Norkeweit J., Schröter D., Schröter A. (2016). Are Neglected Plants the Food for the Future?. CRC Crit. Rev. Plant Sci..

[23] Mabhaudhi T., Chimonyo V.G.P., Hlahla S., Massawe F., Mayes S., Nhamo L., Modi A.T. (2019). Prospects of Orphan Crops in Climate Change. Planta.

[24] Tadele Z. (2018). African Orphan Crops under Abiotic Stresses: Challenges and Opportunities. Scientifica.

[25] Ulian T., Diazgranados M., Pironon S., Padulosi S., Liu U., Davies L., Howes M.R., Borrell J.S., Ondo I., Pérez-Escobar O.A. (2020). Unlocking Plant Resources to Support Food Security and Promote Sustainable Agriculture. Plants People Planet.

[26] Mabhaudhi T., O’Reilly P., Walker S., Mwale S. (2016). Opportunities for Underutilised Crops in Southern Africa’s Post–2015 Development Agenda. Sustainability.

[27] Mabhaudhi T., Chimonyo V.G.P., Chibarabada T.P., Modi A.T. (2017). Developing a Roadmap for Improving Neglected and Underutilized Crops: A Case Study of South Africa. Front. Plant Sci..

[28] Vélez-Gavilán J. Moringa Oleifera (Horse Radish Tree). https://www.cabidigitallibrary.org/doi/10.1079/cabicompendium.34868.

[29] Encyclopædia Britannica Horseradish Tree. https://www.britannica.com/plant/horseradish-tree.

[30] USDA Taxon: Moringa Oleifera Lam. https://npgsweb.ars-grin.gov/gringlobal/taxon/taxonomydetail?id=24597.

[31] Olson M.E. Moringaceae—Moringa. http://www.efloras.org/florataxon.aspx?flora_id=1&taxon_id=200009759.

[32] Lamarck J.-B. (1785). Encyclopédie méthodique. Botanique.

[33] Radovich T., Elevitch C.R. (2011). Farm and Forestry Production and Marketing Profile for Moringa (*Moringa oleifera*). Specialty Crops for Pacific Island Agroforestry.

[34] Lim T.K. (2012). Moringa Oleifera. Edible Medicinal and Non Medicinal Plants.

[35] Tamilselvi N.A., Arumugam T. (2019). Health Benefits, Therapeutic and Pharmacological Properties of Moringa—A Review. J. Pharm. Res. Int..

[36] Peter K.V. (2008). Underutilized and Underexploited Horticultural Crops.

[37] Luoh J.W., Sheu A., Wu W.-J., Yang R.-Y. (2017). Phytonutrient Values of *Moringa oleifera* Leaves. Acta Hortic..

[38] Lyons G., Gondwe C., Banuelos G., Mendoza C., Haug A., Christophersen O., Ebert A.W. (2017). Drumstick Tree (*Moringa oleifera*) Leaves as a Source of Dietary Selenium, Sulphur and pro-Vitamin A. Acta Hortic..

[39] Oyeyinka A.T., Oyeyinka S.A. (2018). *Moringa Oleifera* as a Food Fortificant: Recent Trends and Prospects. J. Saudi Soc. Agric. Sci..

[40] Chinma C.E., Abu J.O., Akoma S.N. (2014). Effect of Germinated Tigernut and Moringa Flour Blends on the Quality of Wheat-Based Bread. J. Food Process Preserv..

[41] Teye E., Deha C.I., Dadzie R., MacArthur R.L. (2020). Delivering the Nutritional Needs by Food to Food Fortification of Staples Using Underutilized Plant Species in Africa. Int. J. Food Sci..

[42] Domenech Asensi G., Durango Villadiego A.M., Ros Berruezo G. (2017). *Moringa Oleifera*: A Review of Food Applications. Arch. Latinoam. Nutr..

[43] United States Department of the Army (2009). The Complete Guide to Edible Wild Plants.

[44] Lea M. (2010). Bioremediation of Turbid Surface Water Using Seed Extract from *Moringa oleifera* Lam. (Drumstick) Tree. Curr. Protoc. Microbiol..

[45] Ndabigengesere A., Narasiah K.S., Talbot B.G. (1995). Active Agents and Mechanism of Coagulation of Turbid Waters Using *Moringa oleifera*. Water Res..

[46] Hellsing M.S., Kwaambwa H.M., Nermark F.M., Nkoane B.B.M., Jackson A.J., Wasbrough M.J., Berts I., Porcar L., Rennie A.R. (2014). Structure of Flocs of Latex Particles Formed by Addition of Protein from *Moringa* Seeds. Colloids Surf. A Physicochem. Eng. Asp..

[47] Ghebremichael K.A., Gunaratna K.R., Henriksson H., Brumer H., Dalhammar G. (2005). A Simple Purification and Activity Assay of the Coagulant Protein from *Moringa oleifera* Seed. Water Res..

[48] Rashid U., Anwar F., Moser B.R., Knothe G. (2008). *Moringa oleifera* Oil: A Possible Source of Biodiesel. Bioresour. Technol..

[49] NPCS Board (2012). Handbook on Agro Based Industries.

[50] Horn L., Shakela N., Mutorwa M.K., Naomab E., Kwaambwa H.M. (2022). *Moringa oleifera* as a Sustainable Climate-Smart Solution to Nutrition, Disease Prevention, and Water Treatment Challenges: A Review. J. Agric. Food Res..

[51] Usai R., Majoni S., Rwere F. (2022). Natural Products for the Treatment and Management of Diabetes Mellitus in Zimbabwe—A Review. Front. Pharmacol..

[52] Odukoya J.O., Odukoya J.O., Mmutlane E.M., Ndinteh D.T. (2022). Ethnopharmacological Study of Medicinal Plants Used for the Treatment of Cardiovascular Diseases and Their Associated Risk Factors in Sub-Saharan Africa. Plants.

[53] Alia F., Putri M., Anggraeni N., Syamsunarno M.R.A.A. (2022). The Potency of *Moringa oleifera* Lam. as Protective Agent in Cardiac Damage and Vascular Dysfunction. Front. Pharmacol..

[54] Liu R., Liu J., Huang Q., Liu S., Jiang Y. (2022). *Moringa oleifera*: A Systematic Review of Its Botany, Traditional Uses, Phytochemistry, Pharmacology and Toxicity. J. Pharm. Pharmacol..

[55] Peter E.L., Nagendrappa P.B., Hilonga S., Tuyiringire N., Ashuro E., Kaligirwa A., Sesaazi C.D. (2021). Pharmacological Reflection of Plants Traditionally Used to Manage Diabetes Mellitus in Tanzania. J. Ethnopharmacol..

[56] Adeleye O.A., Femi-Oyewo M.N., Bamiro O.A., Bakre L.G., Alabi A., Ashidi J.S., Balogun-Agbaje O.A., Hassan O.M., Fakoya G. (2021). Ethnomedicinal Herbs in African Traditional Medicine with Potential Activity for the Prevention, Treatment, and Management of Coronavirus Disease 2019. Futur. J. Pharm. Sci..

[57] Aumeeruddy M.Z., Mahomoodally M.F. (2020). Traditional Herbal Therapies for Hypertension: A Systematic Review of Global Ethnobotanical Field Studies. S. Afr. J. Bot..

[58] Omara T., Kiprop A.K., Ramkat R.C., Cherutoi J., Kagoya S., Moraa Nyangena D., Azeze Tebo T., Nteziyaremye P., Nyambura Karanja L., Jepchirchir A. (2020). Medicinal Plants Used in Traditional Management of Cancer in Uganda: A Review of Ethnobotanical Surveys, Phytochemistry, and Anticancer Studies. Evid. Based Complement. Altern. Med..

[59] Padayachee B., Baijnath H. (2020). An Updated Comprehensive Review of the Medicinal, Phytochemical and Pharmacological Properties of *Moringa oleifera*. S. Afr. J. Bot..

[60] Singh A.K., Rana H.K., Tshabalala T., Kumar R., Gupta A., Ndhlala A.R., Pandey A.K. (2020). Phytochemical, Nutraceutical and Pharmacological Attributes of a Functional Crop *Moringa oleifera* Lam: An Overview. S. Afr. J. Bot..

[61] Naidoo K.K., Coopoosamy R.M. (2011). Review on Herbal Remedies Used by the 1860 South African Indian Settlers. Afr. J. Biotechnol..

[62] Outani B.A., Adamou H., Mahamadou A., Delmas P. (2023). Moringa (*Moringa oleifera* Lam): A Review on Its Importance Worldwide. East Afr. Sch. J. Agric. Life Sci..

[63] Xiao X., Wang J., Meng C., Liang W., Wang T., Zhou B., Wang Y., Luo X., Gao L., Zhang L. (2020). *Moringa oleifera* Lam and Its Therapeutic Effects in Immune Disorders. Front. Pharmacol..

[64] Abd Rani N.Z., Husain K., Kumolosasi E. (2018). Moringa Genus: A Review of Phytochemistry and Pharmacology. Front. Pharmacol..

[65] Makkar H.P.S., Francis G., Becker K. (2007). Bioactivity of Phytochemicals in Some Lesser-Known Plants and Their Effects and Potential Applications in Livestock and Aquaculture Production Systems. Animal.

[66] Mahajan S.G., Mali R.G., Mehta A.A. (2007). Protective Effect of Ethanolic Extract of Seeds of *Moringa oleifera* Lam. Against Inflammation Associated with Development of Arthritis in Rats. J. Immunotoxicol..

[67] Mbokane E.M., Moyo N.A.G. (2022). Use of Medicinal Plants as Feed Additives in the Diets of Mozambique Tilapia (*Oreochromis mossambicus*) and the African Sharptooth Catfish (*Clarias gariepinus*) in Southern Africa. Front. Vet. Sci..

[68] Melo V., Vargas N., Quirino T., Calvo C. (2013). *Moringa Oleifera* L. An Underutilized Tree with Macronutrients for Human Health. Emir. J. Food Agric..

[69] National Research Council (2006). Moringa. Lost Crops of Africa.

[70] Conti M.V., Kalmpourtzidou A., Lambiase S., De Giuseppe R., Cena H. (2021). Novel Foods and Sustainability as Means to Counteract Malnutrition in Madagascar. Molecules.

[71] Amaglo N.K., Deng J., Foidl N. (2017). The Potential of Moringa in Climate Change, Sustainable Livelihood and Food Security—A Review. Acta Hortic..

[72] Keatinge J.D.H., Ebert A.W., Hughes J., Yang R.-Y., Curaba J. (2017). Seeking to Attain the UN’s Sustainable Development Goal 2 Worldwide: The Important Role of *Moringa oleifera*. Acta Hortic..

[73] FAO Traditional Crops—Moringa. https://www.fao.org/traditional-crops/moringa/en.

[74] Moher D., Liberati A., Tetzlaff J., Altman D.G. (2009). Preferred Reporting Items for Systematic Reviews and Meta-Analyses: The PRISMA Statement. PLoS Med..

[75] Page M.J., McKenzie J.E., Bossuyt P.M., Boutron I., Hoffmann T.C., Mulrow C.D., Shamseer L., Tetzlaff J.M., Akl E.A., Brennan S.E. (2021). The PRISMA 2020 Statement: An Updated Guideline for Reporting Systematic Reviews. BMJ.

[76] El Bilali H. (2021). Organic Food and Farming in West Africa: A Systematic Review. Landbauforsch. J. Sustain. Org. Agric. Syst..

[77] El Bilali H., Dambo L., Nanema J., Bassole I.H.N., Calabrese G. (2022). Biodiversity-Pastoralism Nexus in West Africa. AIMS Agric. Food.

[78] Adetola O.Y., Taylor J.R.N., Duodu K.G. (2023). Can Consumption of Local Micronutrient- and Absorption Enhancer-Rich Plant Foods Together with Starchy Staples Improve Bioavailable Iron and Zinc in Diets of at-risk African Populations?. Int. J. Food Sci. Nutr..

[79] Frazzoli C., Grasso G., Husaini D.C., Ajibo D.N., Orish F.C., Orisakwe O.E. (2023). Immune System and Epidemics: The Role of African Indigenous Bioactive Substances. Nutrients.

[80] Golla S.P., Ramesh Kumar R., Veerapandian C., Rangarajan J., Mariya Anthony T.A. (2022). Meta Analysis on Color, Total Flavonoids, Antioxidants, Frictional, Mechanical, and Cutting Force of Moringa Pods. J. Food Process Eng..

[81] Ichrak B. (2022). Anaphylaxis to *Moringa Oleifera* in North Africa: A Case Report and Review of the Literature. Clin. Case Rep..

[82] Saadat S., Beigoli S., Khazdair M.R., Amin F., Boskabady M.H. (2022). Experimental and Clinical Studies on the Effects of Natural Products on Noxious Agents-Induced Lung Disorders, a Review. Front. Nutr..

[83] Jain A., Poli Y., Goud M.D., Ravi R.S.D., Bhaskaran S., Wang X., Das S.S., Gupta S., Jain R., Kachhwaha S. (2021). A Comprehensive Review on the Biotechnological Intervention for Deciphering the Pharmacological and Other Multifarious Properties of Miracle Tree *Moringa oleifera*. Ind. Crops Prod..

[84] Losso J.N., Losso M.N., Toc M., Inungu J.N., Finley J.W. (2021). The Young Age and Plant-Based Diet Hypothesis for Low SARS-CoV-2 Infection and COVID-19 Pandemic in Sub-Saharan Africa. Plant Foods Human. Nutr..

[85] George T.T., Obilana A.O., Oyenihi A.B., Rautenbach F.G. (2021). *Moringa oleifera* through the Years: A Bibliometric Analysis of Scientific Research (2000–2020). S. Afr. J. Bot..

[86] Mashamaite C.V., Pieterse P.J., Mothapo P.N., Phiri E.E. (2021). *Moringa oleifera* in South Africa: A Review on Its Production, Growing Conditions and Consumption as a Food Source. S. Afr. J. Sci..

[87] Moyo N.A.G., Rapatsa M.M. (2021). A Review of the Factors Affecting Tilapia Aquaculture Production in Southern Africa. Aquaculture.

[88] du Toit E.S. (2021). Horticultural Research Review on *Moringa oleifera* Trees Growing in South Africa under Sub-Optimal Conditions. Acta Hortic..

[89] Gariba S.Y., Dzidzienyo D.K., Eziah V.Y. (2021). Assessment of Four Plant Extracts as Maize Seed Protectants against *Sitophilus zeamais* and *Prostephanus truncatus* in Ghana. Cogent Food Agric..

[90] Mollel M.H., Mujuru M., Mjimba V., Darangwa N., Nyanzi R. (2021). *Moringa oleifera* Noise: Science or Quackery and the Way Forward. Afr. J. Sci. Technol. Innov. Dev..

[91] Mashamaite C.V., Mothapo P.N., Albien A.J., Pieterse P.J., Phiri E.E. (2020). A SUSPECT under the National Environmental Management Biodiversity Act (NEM:BA) *Moringa oleifera*’s Ecological and Social Costs and Benefits. S. Afr. J. Bot..

[92] Muzumbukilwa W.T., Kadima M.G., Nlooto M., Owira P.M.O. (2019). Mapping the Evidence of Hepatoprotective Properties of *Moringa oleifera* from Sub-Saharan African Countries: A Systematic Review Protocol. Syst. Rev..

[93] Vergara-Jimenez M., Almatrafi M., Fernandez M. (2017). Bioactive Components in *Moringa oleifera* Leaves Protect against Chronic Disease. Antioxidants.

[94] Olson M.E. (2017). Moringa Frequently Asked Questions. Acta Hortic..

[95] Palada M.C., Ebert A.W., Yang R.-Y., Chang L.C., Chang J., Wu D.L. (2017). Progress in Research and Development of Moringa at the World Vegetable Center. Acta Hortic..

[96] Pasternak D., Keatinge J.D.H., Mamoudou Z. (2017). Moringa Research and Cultivation in Niger. Acta Hortic..

[97] Tsouh Fokou P.V., Nyarko A.K., Appiah-Opong R., Tchokouaha Yamthe L.R., Addo P., Asante I.K., Boyom F.F. (2015). Ethnopharmacological Reports on Anti-Buruli Ulcer Medicinal Plants in Three West African Countries. J. Ethnopharmacol..

[98] Thelingwani R., Masimirembwa C. (2014). Evaluation of Herbal Medicines: Value Addition to Traditional Medicines through Metabolism, Pharmacokinetic and Safety Studies. Curr. Drug Metab..

[99] Shahzad U., Khan M.A., Jaskani M.J., Khan I.A., Korban S.S. (2013). Genetic Diversity and Population Structure of *Moringa oleifera*. Conserv. Genet..

[100] Alao J.S. (2011). Problems and Management of Insect Pests in Social Forestry in Nigeria. Afr. J. Agric. Res..

[101] McBurney R.P.H., Griffin C., Paul A.A., Greenberg D.C. (2004). The Nutritional Composition of African Wild Food Plants: From Compilation to Utilization. J. Food Compos. Anal..

[102] El Bilali H. (2020). Orphan Crops in Burkina Faso and Niger: A Systematic Review. CAB Rev. Perspect. Agric. Veter-Sci. Nutr. Nat. Resour..

[103] El Bilali H. (2019). Research on Agro-Food Sustainability Transitions: Where Are Food Security and Nutrition?. Food Secur..

[104] Nwosu M.O., Okafor J.I. (1995). Preliminary Studies of the Antifungal Activities of Some Medicinal Plants against *Basidiobolus* and Some Other Pathogenic Fungi. Mycoses.

[105] Kumssa D.B., Joy E.J.M., Young S.D., Odee D.W., Ander E.L., Magare C., Gitu J., Broadley M.R. (2017). Challenges and Opportunities for Moringa Growers in Southern Ethiopia and Kenya. PLoS ONE.

[106] de Saint Sauveur A., Amenglor M.G., Kinda J., Colomban F. (2017). Enhancing Nutrient Intake from *Moringa* Leaves through Adequate Consumption Patterns. Acta Hortic..

[107] Sibeko L., Johns T. (2021). Global Survey of Medicinal Plants during Lactation and Postpartum Recovery: Evolutionary Perspectives and Contemporary Health Implications. J. Ethnopharmacol..

[108] Boukandoul S., Casal S., Cruz R., Pinho C., Zaidi F. (2017). Algerian *Moringa oleifera* Whole Seeds and Kernels Oils: Characterization, Oxidative Stability, and Antioxidant Capacity. Eur. J. Lipid Sci. Technol..

[109] Boulaadjoul S., Zemmouri H., Bendjama Z., Drouiche N. (2018). A Novel Use of *Moringa oleifera* Seed Powder in Enhancing the Primary Treatment of Paper Mill Effluent. Chemosphere.

[110] Braham F., Carvalho D.O., Almeida C.M.R., Zaidi F., Magalhães J.M.C.S., Guido L.F., Gonçalves M.P. (2020). Online HPLC-DPPH Screening Method for Evaluation of Radical Scavenging Phenols Extracted from *Moringa oleifera* Leaves. S. Afr. J. Bot..

[111] Kebaili M., Djellali S., Drouiche N., Lounici H. (2022). New Biopolymer from Biomass for Conditioning and Dehydration of Sewage Sludge: Application on the Sludge of Bouira WWTP. Environ. Sci. Pollut. Res..

[112] Ouahrani S., Tzompa-Sosa D.A., Dewettinck K., Zaidi F. (2022). Oxidative Stability, Structural, and Textural Properties of Margarine Enriched with *Moringa oleifera* Leaves Extract. J. Am. Oil Chem. Soc..

[113] Adéyèmi A.D., Kayodé A.P.P., Chabi I.B., Odouaro O.B.O., Nout M.J.R., Linnemann A.R. (2020). Screening Local Feed Ingredients of Benin, West Africa, for Fish Feed Formulation. Aquac. Rep..

[114] Affonfere M., Chadare F.J., Fassinou F.T.K., Talsma E.F., Linnemann A.R., Azokpota P. (2021). A Complementary Food Supplement from Local Food Ingredients to Enhance Iron Intake among Children Aged 6–59 Months in Benin. Food Sci. Nutr..

[115] Agoyi E.E., Okou F.A.Y., Assogbadjo E.A., Sinsin B. (2017). Medicinal Uses of *Moringa oleifera* in Southern Benin (West Africa). Acta Hortic..

[116] Bachabi F., Togbe C.E., Odjo T., Were E.O., Sere Y., Gumedzoe Y.M.D. (2018). Laboratory Assessment of the Antibacterial Effect of *Moringa oleifera* Lam., *Azadirachta indica* L. and *Jatropha curcas* L. on *Pantoea* spp. and *Sphingomonas* spp. in Rice Seed in Benin. Trop. Agric..

[117] Dougnon G., Ito M. (2020). Medicinal Uses, Thin-Layer Chromatography and High-Performance Liquid Chromatography Profiles of Plant Species from Abomey-Calavi and Dantokpa Market in the Republic of Benin. J. Nat. Med..

[118] Gandji K., Salako V.K., Fandohan A.B., Assogbadjo A.E., Glèlè Kakaï R.L. (2018). Factors Determining the Use and Cultivation of *Moringa oleifera* Lam. in the Republic of Benin. Econ. Bot..

[119] Gandji K., Tovissodé F.C., Azihou A.F., Akpona J.D.T., Assogbadjo A.E., Kakaï R.L.G. (2020). Morphological Diversity of the Agroforestry Species *Moringa oleifera* Lam. as Related to Ecological Conditions and Farmers’ Management Practices in Benin (West Africa). S. Afr. J. Bot..

[120] Noulèkoun F., Khamzina A., Naab J., Khasanah N., van Noordwijk M., Lamers J. (2018). Climate Change Sensitivity of Multi-Species Afforestation in Semi-Arid Benin. Sustainability.

[121] Noulèkoun F., Lamers J.P.A., Naab J., Khamzina A. (2017). Shoot and Root Responses of Woody Species to Silvicultural Management for Afforestation of Degraded Croplands in the Sudano-Sahelian Zone of Benin. For. Ecol. Manag..

[122] Noulèkoun F., Naab J.B., Lamers J.P.A., Baumert S., Khamzina A. (2018). Sapling Biomass Allometry and Carbon Content in Five Afforestation Species on Marginal Farmland in Semi-Arid Benin. New For..

[123] Noulèkoun F., Khamzina A., Naab J.B., Lamers J.P.A. (2017). Biomass Allocation in Five Semi-Arid Afforestation Species Is Driven Mainly by Ontogeny Rather than Resource Availability. Ann. For. Sci..

[124] Bombo K., Lekgoba T., Azeez O., Muzenda E. (2021). Production of Biodiesel from *Moringa oleifera* and *Jatropha curcas* Seed Oils over a Modified ZnO/Fly Ash Catalyst. Environ. Clim. Technol..

[125] Nduwayezu J.B., Chamshama S.A.O., Mugasha A.G., Ngaga Y.N., Khonga E.B., Chabo R.G. (2007). Comparisons in Seed Kernel Sizes and Early Growth Performance of Different *Moringa oleifera* Provenances in Southeast of Botswana. Discov. Innov..

[126] Ntshambiwa K.T., Seifu E., Mokhawa G. (2023). Nutritional Composition, Bioactive Components and Antioxidant Activity of *Moringa stenopetala* and *Moringa oleifera* Leaves Grown in Gaborone, Botswana. Food Prod. Process. Nutr..

[127] Seifu E., Teketay D. (2020). Introduction and Expansion of *Moringa oleifera* Lam. in Botswana: Current Status and Potential for Commercialization. S. Afr. J. Bot..

[128] Semanka T., Seifu E., Sekwati-Monang B. (2022). Effects of *Moringa oleifera* Seeds on the Physicochemical Properties and Microbiological Quality of Borehole Water from Botswana. J. Water Sanit. Hyg. Dev..

[129] Amante B., López-Grimau V., Smith T. (2016). Valuation of Oil Extraction Residue from *Moringa oleifera* Seeds for Water Purification in Burkina Faso. Desalination Water Treat..

[130] Amante-García B., López Grimau V., Canals Casals L. (2017). LCA of Different Energy Sources for a Water Purification Plant in Burkina Fasso. Desalination Water Treat..

[131] Etongo D., Djenontin I., Kanninen M., Fobissie K. (2015). Smallholders’ Tree Planting Activity in the Ziro Province, Southern Burkina Faso: Impacts on Livelihood and Policy Implications. Forests.

[132] Krieg J., Goetze D., Porembski S., Arnold P., Linsenmair K.E., Stein K. (2017). Floral and Reproductive Biology of *Moringa oleifera* (Moringaceae) in Burkina Faso, West Africa. Acta Hortic..

[133] Lopez-Grimau V., Smith T., Amante B., Heras F. (2013). Treatment Plant Design Using Natural Products for the Purification of Surface Waters in Burkina Faso. Afinidad.

[134] Moussavou M.R.W., Brunschwig C., Baréa B., Villeneuve P., Blin J. (2016). Assessing the Enzyme Activity of Different Plant Extracts of Biomasses from Sub-Saharan Africa for Ethyl Biodiesel Production. Energy Fuels.

[135] Arsene M., Viktorovna P., Davares A. (2021). *Galleria mellonella* (Greater Wax Moth) as an Eco-Friendly in vivo Approach for the Assessment of the Acute Toxicity of Medicinal Plants: Application to Some Plants from Cameroon. Open Vet. J..

[136] Mawouma S., Ponka R., Mbofung C.M. (2017). Acceptability and Solubility of Iron and Zinc Contents of Modified *Moringa oleifera* Sauces Consumed in the Far-North Region of Cameroon. Food Sci. Nutr..

[137] Ngounouno M.A., Ngueyep L.L.M., Kingni S.T., Nforsoh S.N., Ngounouno I. (2021). Evaluation of the Impact of Gold Mining Activities on the Waters and Sediments of Lom River, Wakaso, Cameroon and the Restorative Effect of *Moringa oleifera* Seeds. Appl. Water Sci..

[138] Rébufa C., Dupuy N., Bombarda I. (2021). AComDim, a Multivariate Tool to Highlighting Impact of Agroclimatic Factors on *Moringa oleifera* Lam. Leaf’s Composition from Their FTIR-ATR Profiles. Vib. Spectrosc..

[139] Saha B.U.F., Choumessi A.T., Ayamo A.M., Kuagny R.B.M., Teta I., Nantia E.A., Ejoh R.A. (2022). Nutritional Quality of Three Iron-Rich Porridges Blended with *Moringa oleifera*, *Hibiscus sabdariffa*, and *Solanum aethiopicum* to Combat Iron Deficiency Anemia among Children. J. Food Qual..

[140] Ngandjui Tchangoue Y.A., Djumyom Wafo G.V., Wanda C., Soh Kengne E., Kengne I.M., Kouam Fogue S. (2019). Use of *Moringa oleifera* Seed Extracts to Polish Effluents from Natural Systems Treating Faecal Sludge. Environ. Technol..

[141] Djawe Yannick W., Sala T., Noumi Valery N. (2022). Ecological and structural study of the *Moringa oleifera* population in the Sudano-Sahelian zone: Case of the far north of Cameroon. Int. J. Ecosyst. Ecol. Sci. (IJEES).

[142] Yongabi K.A., Mbacham W.F., Nubia K.K., Singh R.M. (2009). Yeast Strains Isolated from HIV-Seropositive Patients in Cameroon and Their Sensitivity to Extracts of Eight Medicinal Plants. Afr. J. Microbiol. Res..

[143] Yongabi K.A., Lewis D.M., Harris P.L. (2011). Application of Phytodisinfectants in Water Purification in Rural Cameroon. Afr. J. Microbiol. Res..

[144] Yongabi K.A., Lewis D.M., Harris P.L. (2011). Indigenous Plant Based Coagulants/Disinfectants and Sand Filter Media for Surface Water Treatment in Bamenda, Cameroon. Afr. J. Biotechnol..

[145] Poumaye N., Mabingui J., Lutgen P., Bigan M. (2012). Contribution to the Clarification of Surface Water from the *Moringa oleifera*: Case M’Poko River to Bangui, Central African Republic. Chem. Eng. Res. Des..

[146] Leone A., Fiorillo G., Criscuoli F., Ravasenghi S., Santagostini L., Fico G., Spadafranca A., Battezzati A., Schiraldi A., Pozzi F. (2015). Nutritional Characterization and Phenolic Profiling of *Moringa oleifera* Leaves Grown in Chad, Sahrawi Refugee Camps, and Haiti. Int. J. Mol. Sci..

[147] Abrogoua D.P., Dano D.S., Manda P., Adepo A.J.-B., Kablan B.J., Goze N.B., Ehoulé K. (2012). Effect on Blood Pressure of a Dietary Supplement Containing Traditional Medicinal Plants of Côte d’Ivoire. J. Ethnopharmacol..

[148] Kpan W.B., Koné M.W., Bonfoh B., Kamanzi K. (2017). Evaluation of Eighteen West African Plants for Water Purification, Potential Use for Rural Water Treatment. J. Water Chem. Technol..

[149] Kambashi B., Picron P., Boudry C., Théwis A., Kiatoko H., Bindelle J. (2014). Nutritive Value of Tropical Forage Plants Fed to Pigs in the Western Provinces of the Democratic Republic of the Congo. Anim. Feed. Sci. Technol..

[150] Kampemba F.M., Tshibangu I.M., Nyongombe N.U., Hornick J.-L. (2017). Palatability of Nine Fodders Species Used by Guinea Pigs (*Cavia porcellus*). Trop. Anim. Health Prod..

[151] Mushagalusa Kasali F., Ahadi Irenge C., Murhula Hamuli P., Birindwa Mulashe P., Murhula Katabana D., Mangambu Mokoso J.D.D., Mpiana P.T., Ntokamunda Kadima J. (2021). Ethnopharmacological Survey on Treatment of Hypertension by Traditional Healers in Bukavu City, DR Congo. Evid. Based Complement. Altern. Med..

[152] Tshingani K., Donnen P., Mukumbi H., Duez P., Dramaix-Wilmet M. (2017). Impact of *Moringa oleifera* Lam. Leaf Powder Supplementation versus Nutritional Counseling on the Body Mass Index and Immune Response of HIV Patients on Antiretroviral Therapy: A Single-Blind Randomized Control Trial. BMC Complement. Altern. Med..

[153] Abdalla A., Sadak M., Abd Elhamid E., Ezo M. (2022). Amelioration of Drought Stress Reduced Effects by Exogenous Application of L-Phenylalanine on *Moringa oleifera*. Egypt. J. Chem..

[154] Abdallah A.M., Ismail M.S.M., AboGhalia A.H., Soliman M.F.M. (2019). Factors Affecting Population Dynamics of *Tetranychus urticae* and Its Predators on Three Economic Plants in Ismailia, Egypt. Int. J. Trop. Insect Sci..

[155] Abdel Moniem S., Youssef N., Mazhar A., Ibrahim E. (2021). Valorizing the Reuse of Treated Municipal Wastewater for Paulownia Seedlings Cultivation by Application of Moringa Waste. Egypt. J. Chem..

[156] Abdelsayed E.M., Abdel Motaal A., Hanafi R.S. (2021). Novel UPLC-MS/MS Method for Standardization of Niazimicin Content in Edible Seeds and Leaves of *Moringa oleifera* Lam. J. Food Drug Anal..

[157] Abdelwanis F., Hosni A., Abdelhamid A., Sulman A., Ezoo M., Saleh S. (2022). Effect of Zinc and Boron Foliar Application on Leaf Chemical Composition of *Moringa oleifera* and on Yield and Characters of Its Seed Oil. Egypt. J. Chem..

[158] Abdelwanise F.M., Saleh S.A., Ezzo M.I., Helmy S.S., Abodahab M.A. (2017). Response of Moringa Plants to Foliar Application of Nitrogen and Potassium Fertilizers. Acta Hortic..

[159] Darwish S.N.A., Ezzo M.I., Morsy F.I., Soliman A.S., Glala A.A., Abdalla A.M. (2021). Nitrogen Fertilization Strategy for *Moringa oleifera* as an Introduced Leafy Vegetable Crop in Egypt. Acta Hortic..

[160] Desoky E., Merwad A.-R., Ibrahim S. (2019). Humus Materials and Moringa (*Moringa oleifera* Lam.) Leaf Extract Modulate the Harmful Effect of Soil Salinity Stress in Sudan Grass (*Sorghum vulgare* L.). Egypt. J. Agron..

[161] El-Boraie F.M., El Shafay M.M., Elhagarey M.E. (2020). Integrated System for Maximizing Water Unit Productivity of *Moringa* Vegetative Yield under Shalatien Conditions. Biosci. Res..

[162] El-Hadidy G., Mahmoud T., Shaaban F., Hemdan N. (2021). Effect of Organic Fertilization with *Moringa oleifera* Seeds Cake and Compost on Storability of Valencia Orange Fruits. Egypt. J. Chem..

[163] El-Helaly A. (2020). Moringa Water Extract Promising Additive to Prolong the Activity of Baculovirus under Field-Sunlight Conditions in Egypt. Braz. J. Biol..

[164] Ezzo M., Ebtihal M.A.E., Sadak M.S., Abdalla A.M. (2018). Improving Drought Tolerance of Moringa Plants by Using Trehalose Foliar Treatments. Biosci. Res..

[165] Hamada M., Eldaim M.A., Fathalla S.I., El Behiry A., Alkafafy M. (2021). The Potential Impact of *Moringa oleifera* for Diminishing the Microbial Contamination and Prolonging the Quality and Shelf-Life of Chilled Meat. J. Pure Appl. Microbiol..

[166] Hegazi A.G., Abdel Megeed K.N., Hassan S.E., Abdelaziz M.M., Toaleb N.I., El Shanawany E.E., Aboelsoued D. (2018). Comparative Ovicidal Activity of *Moringa oleifera* Leaf Extracts on *Fasciola gigantica* Eggs. Vet. World.

[167] Hemdan N.A., Mahmoud T.S.M., Abdalla A.M., Man-Sour H.A. (2021). Using *Moringa oleifera* Seed Cake and Compost as Organic Soil Amendments for Sustainable Agriculture in Valencia Orange Orchard. Future Food J. Food Agric. Soc..

[168] Ibrahim A.M., Abdalla A.M. (2017). Impact of *Moringa oleifera* Seed Aqueous Extract on Some Biological, Biochemical, and Histological Aspects of *Biomphalaria alexandrina* Snails. Environ. Sci. Pollut. Res..

[169] Ibrahim A.A., Namich A.A. (2021). Amelioration of the Adverse Effects of Salinity Stress by Using Bio-Stimulant Moringa on Cotton Plant. Egypt. J. Agron..

[170] Ibrahim I.K.A., Abu-Habib A.H.A., Kantor M., Handoo Z.A. (2022). Pathogenicity and Control of The Citrus Nematode *Tylenchulus semipenetrans* on Citrus, Grape, Olive, Loquat, and Persimmon Species and Cultivars. Nematropica.

[171] Mansour M., Mohamed M.F., Elhalwagi A., El-Itriby H.A., Shawki H.H., Abdelhamid I.A. (2019). Moringa Peregrina Leaves Extracts Induce Apoptosis and Cell Cycle Arrest of Hepatocellular Carcinoma. Biomed. Res. Int..

[172] Merwad A.-R.M.A. (2018). Using *Moringa oleifera* Extract as Biostimulant Enhancing the Growth, Yield and Nutrients Accumulation of Pea Plants. J. Plant Nutr..

[173] Merwad A.-R.M.A. (2020). Mitigation of Salinity Stress Effects on Growth, Yield and Nutrient Uptake of Wheat by Application of Organic Extracts. Commun. Soil. Sci. Plant Anal..

[174] Merwad A.-R.M. (2017). Effect of Humic and Fulvic Substances and Moringa Leaf Extract on Sudan Grass Plants Grown under Saline Conditions. Can. J. Soil. Sci..

[175] Merwad A.-R., Abdel-Fattah M.K. (2017). Improving Productivity and Nutrients Uptake of Wheat Plants Using *Moringa oleifera* Leaf Extract in Sandy Soil. J. Plant Nutr..

[176] Mohamed M., Ibrahim M., Abdel-Azim N., El-Missiry M. (2021). Chemical and Biological Studies on *Moringa oleifera* L. Cultivated in Egypt. Egypt. Pharm. J..

[177] Mohamed S., Youssef A., Issa M., Abdel Salam H., El-Ansary A. (2020). Validated HPLC Method for Quantitative Analysis of Gallic Acid and Rutin in Leaves of *Moringa oleifera* Grown in Egypt. Egypt. J. Chem..

[178] Mohamed Y.M.A., Osman S.A., Elshahavv I.E., Soliman G.M., Ahmed A.M.A. (2023). Charcoal Rot and Root-Knot Nematode Control on Faba Bean by Photosynthesized Colloidal Silver Nanoparticles Using Bioactive Compounds from *Moringa oleifera* Leaf Extract. J. Plant Prot. Res..

[179] Monir W., Abdel-Rahman M.A., El-Din Hassan S., Mansour E.S., Awad S.M.M. (2020). Pomegranate Peel and Moringa-Based Diets Enhanced Biochemical and Immune Parameters of Nile Tilapia against Bacterial Infection by *Aeromonas hydrophila*. Microb. Pathog..

[180] Mosa W.F.A., Sas-Paszt L., Głuszek S., Górnik K., Anjum M.A., Saleh A.A., Abada H.S., Awad R.M. (2022). Effect of Some Biostimulants on the Vegetative Growth, Yield, Fruit Quality Attributes and Nutritional Status of Apple. Horticulturae.

[181] Nofal E.M.S., El-Mahrouk M.E., El-Sayed B.A., Radwan A.M.M. (2021). Effect of NPK Fertilizer and Some Natural Extract Treatments on the Chemical Composition of African Marigold (*Tagetes erecta* L. Var. Dwarf Chrysanthemum). Appl. Ecol. Environ. Res..

[182] Nofal E.M.S., Khalafallah M.M., Shahin S.M., Montasser H.M.M.S. (2021). Usage of Magnetic Iron to Raise Tolerance of Some Ornamental Trees and Shrubs to Soil Salinity in Case of Horseradish Tree (*Moringa oleifera* Lam.). Appl. Ecol. Environ. Res..

[183] Osman A.R., El-Naggar H.M. (2022). Enhancing Salinity Stress Tolerance and Phenylalanine Ammonia Lyase Gene Activity Using Osmolytes in *Moringa* Seedling Production. Ann. Agric. Sci..

[184] Salama A.A.A., Fayed A.-H.M., Attia T.A., Seham E.A., Ismaiel I.E., Hassan A. (2018). Protective Effects of *Moringa oleifera* Extract on Isoniazid and Rifampicin Induced Hepatotoxicity in Rats: Involvement of Adiponectin and Tumor Necrosis Factor-α. Egypt. J. Vet. Sci..

[185] Sarhan A., Habib A., Fahmy A., Noor El-Deen T., Selim A. (2021). Effect of Nano, Bio, Chemical Fertilization and Leaves Extract of Moringa Plant on Flowering and Chemical Constituents of Gladiolus Plant. Egypt. J. Chem..

[186] Shaaban M.T., Morsi H.H., Aglan A.A. (2019). Physiochemical and Microalgal Investigation of Nile River Water and a Comparison between Seed Extract of *Moringa oleifera* and *Aluminum sulfate* in Removing Microalgae and Turbidity from Water. Biosci. Res..

[187] Thabet A.Y., Abdel-Hak R., Ashour N., Hassan H. (2022). Influence of Foliar Application with Moringa (*Moringa oleifera* L.) Aqueous Leaf Extract on Fruit Set, Yield and Fruit Quality of Selmy Date Palms. Egypt. J. Chem..

[188] Zubuko N.S., Olaleye A.O., Edje O.T. (2019). Effects and Rates of Broiler and Layer Manures on the Leaf Yield and Tissue Nutrient Contents of Moringa (*Moringa oleifera* Lam.) in Swaziland. J. Plant Nutr..

[189] Adefa T., Tefera M. (2020). Heavy Metal Accumulation and Health Risk Assessment in *Moringa oleifera* from Awi Zone, Ethiopia. Chem. Afr..

[190] Bartíková M., Holková L., Šafránková I., Němec P. (2020). Causal Agents of Powdery Mildew on *Moringa stenopetala* (Baker f.) Cuf. and *Moringa oleifera* Lam. in Ethiopia. S. Afr. J. Bot..

[191] Debela E., Tolera A. (2013). Nutritive Value of Botanical Fractions of *Moringa oleifera* and *Moringa stenopetala* Grown in the Mid-Rift Valley of Southern Ethiopia. Agrofor. Syst..

[192] Ejeta D., Asme A., Asefa A. (2021). Insecticidal Effect of Ethnobotanical Plant Extracts against *Anopheles arabiensis* under Laboratory Conditions. Malar. J..

[193] Gebrezgi D. (2019). Proximate Composition of Complementary Food Prepared from Maize (*Zea mays*), Soybean *(Glycine max*) and *Moringa* Leaves in Tigray, Ethiopia. Cogent Food Agric..

[194] Endale Gurmu A., Teni F.S., Tadesse W.T. (2017). Pattern of Traditional Medicine Utilization among HIV/AIDS Patients on Antiretroviral Therapy at a University Hospital in Northwestern Ethiopia: A Cross-Sectional Study. Evid.-Based Complement. Altern. Med..

[195] Habtemariam H., Kifle D., Leta S., Mucci M., Lürling M. (2021). Removal of Cyanobacteria from a Water Supply Reservoir by Sedimentation Using Flocculants and Suspended Solids as Ballast: Case of Legedadi Reservoir (Ethiopia). PLoS ONE.

[196] Mekuria A.B., Belachew S.A., Tegegn H.G., Ali D.S., Netere A.K., Lemlemu E., Erku D.A. (2018). Prevalence and Correlates of Herbal Medicine Use among Type 2 Diabetic Patients in Teaching Hospital in Ethiopia: A Cross-Sectional Study. BMC Complement. Altern. Med..

[197] Melesse A., Steingass H., Schollenberger M., Holstein J., Rodehutscord M. (2019). Nutrient Compositions and in vitro Methane Production Profiles of Leaves and Whole Pods of Twelve Tropical Multipurpose Tree Species Cultivated in Ethiopia. Agrofor. Syst..

[198] Němec P., Ungrová M., Alem S., Novák J., Habrová H. (2020). Biomass Production of a Young Plantation of *Moringa stenopetala* (Baker f.) Cufod. and *Moringa oleifera* Lam. in Southern Ethiopia. S. Afr. J. Bot..

[199] Tafesse A., Goshu D., Gelaw F., Ademe A. (2020). Commercialization of Moringa: Evidence from Southern Ethiopia. Cogent Econ. Financ..

[200] Tafesse A., Goshu D., Gelaw F., Ademe A. (2020). Food and Nutrition Security Impacts of Moringa: Evidence from Southern Ethiopia. Cogent Food Agric..

[201] Acheampong M.A., Pereira J.P.C., Meulepas R.J.W., Lens P.N.L. (2011). Biosorption of Cu(II) onto Agricultural Materials from Tropical Regions. J. Chem. Technol. Biotechnol..

[202] Adu-Dapaah H., Osei-Bonsu I., Oduro I., Asiedu J. (2017). Recent Advances in Production, Processing and Utilization of *Moringa oleifera* in Ghana. Acta Hortic..

[203] Amaglo N.K., Bennett R.N., Lo Curto R.B., Rosa E.A.S., Lo Turco V., Giuffrida A., Curto A.L., Crea F., Timpo G.M. (2010). Profiling Selected Phytochemicals and Nutrients in Different Tissues of the Multipurpose Tree *Moringa oleifera* L., Grown in Ghana. Food Chem..

[204] Amagloh F.K., Benang A. (2009). Effectiveness of *Moringa oleifera* Seed as Coagulant for Water Purification. Afr. J. Agric. Res..

[205] Amagloh F., Atuna R., McBride R., Carey E., Christides T. (2017). Nutrient and Total Polyphenol Contents of Dark Green Leafy Vegetables, and Estimation of Their Iron Bioaccessibility Using the In Vitro Digestion/Caco-2 Cell Model. Foods.

[206] Boateng L., Ashley I., Ohemeng A., Asante M., Steiner-Asiedu M. (2018). Improving Blood Retinol Concentrations with Complementary Foods Fortified with *Moringa oleifera* Leaf Powder—A Pilot Study. Yale J. Biol. Med..

[207] Boateng L., Quarpong W., Ohemeng A., Asante M., Steiner-Asiedu M. (2019). Effect of Complementary Foods Fortified with *Moringa oleifera* Leaf Powder on Hemoglobin Concentration and Growth of Infants in the Eastern Region of Ghana. Food Sci. Nutr..

[208] Clark Y., Jimenez M., Raso E., Antwi L., Ofosu-Appiah L., Opare D., Torondel B. (2018). Evaluation of Key Antimicrobial Properties of *Moringa oleifera* in Relation to Its Use as a Hand-Washing Product. Water.

[209] Cudjoe E., Donu D., Okonu R.E., Amponsah J.A., Amoah L.E. (2020). The In Vitro Antiplasmodial Activities of Aqueous Extracts of Selected Ghanaian Herbal Plants. J. Parasitol. Res..

[210] Doglikuu B.-I.D., Abubakari A., Yaseri M., Shakibazadeh E., Djazayery A., Mirzaei K. (2021). The Potential Role of Plantains, Moringa, Plantain-Moringa Combined Diets, and Other Plant-Based Dietary Patterns in Controlling Glycaemia among T2DM Persons, a Hospital Based Cross Sectional Survey in Ghana. J. Diabetes Metab. Disord..

[211] Glover-Amengor M., Aryeetey R., Afari E., Nyarko A. (2017). Micronutrient Composition and Acceptability of *Moringa oleifera* Leaf-fortified Dishes by Children in Ada-East District, Ghana. Food Sci. Nutr..

[212] Kudzinawo C., Awunyo-Vitor D., Wongnaa C.A. (2022). Empirical Examination of Financial and Economic Viability of *Moringa oleifera* Production in the Bono East Region, Ghana. For. Trees Livelihoods.

[213] Kumah P., Ampomah E.O., Olympio N.S., Moses E. (2013). Efficacy of Four Botanicals and Two Chemical Fungicides in the Control of Crown Rot Disease of Banana (*Musa* spp. AAA) “Medium Cavendish”. Acta Hortic..

[214] Mao J., Addanki T., Gebhard M., Ghio F., Li H. (2020). Eco-Agriculture and Renewable Energy System Concept for a Rural Community in Ghana. IOP Conf. Ser. Earth Environ. Sci..

[215] Kwabena Ntibrey R.A., Kuranchie F.A., Gyasi S.F. (2020). Antimicrobial and Coagulation Potential of *Moringa oleifera* Seed Powder Coupled with Sand Filtration for Treatment of Bath Wastewater from Public Senior High Schools in Ghana. Heliyon.

[216] Ochire-Boadu K., Abunyewa A.A., Kaba J.S., Twum-Ampofo K., Dawoe E.L.K., Agbenyega O., Barnes R.V. (2020). Improved Legume Fallows: Influence on Nitrogen and Microbial Dynamics, and Maize (*Zea mays* L.) Grain Yield in Sub-Humid Zone of West Africa. Cogent Food Agric..

[217] Peprah P., Appiah-Brempong E., Agyemang-Duah W., Okyere P., Gyimah A.A. (2022). ‘Where Were Pharmaceuticals in Eden?’ Use of Herbal Medicine in Old Age: Focus Group Discussions among Community-Dwelling Older Adults in Ghana. J. Herb. Med..

[218] Sarkwa F.O., Adogla-Bessa T., Madibela O.R., Mphinyane W.N., Perkins J.S., Timpong-Jones E.C., Gyimah A.N., Otu B.O., Tudeka C.K. (2023). The Effects of Feeding Dried Browse Leaves on Rumen Ammonia Levels, Methanogens and Protozoa Amplification of Sheep in the Coastal Savannah of Ghana. Trop. Anim. Health Prod..

[219] Senanu B.M., Boakye P., Oduro-Kwarteng S., Sewu D.D., Awuah E., Obeng P.A., Afful K. (2021). Inhibition of Ammonia and Hydrogen Sulphide as Faecal Sludge Odour Control in Dry Sanitation Toilet Facilities Using Plant Waste Materials. Sci. Rep..

[220] Sengupta M.E., Keraita B., Olsen A., Boateng O.K., Thamsborg S.M., Pálsdóttir G.R., Dalsgaard A. (2012). Use of *Moringa oleifera* Seed Extracts to Reduce Helminth Egg Numbers and Turbidity in Irrigation Water. Water Res..

[221] Tabong P.T.-N., Bawontuo V., Dumah D.N., Kyilleh J.M., Yempabe T. (2018). Premorbid Risk Perception, Lifestyle, Adherence and Coping Strategies of People with Diabetes Mellitus: A Phenomenological Study in the Brong Ahafo Region of Ghana. PLoS ONE.

[222] Tetteh O.N.A., Huyskens-Keil S., Amaglo N.K., Amagloh F.K., Oduro I.N., Adarkwah C., Obeng-Ofori D., Ulrichs C., Foerster N. (2021). Effect of Terroir on the Glucosinolate Content of *Moringa oleifera* Grown in Three Agro-Ecological Zones of Ghana. J. Appl. Bot. Food Qual..

[223] Bancessi A., Bancessi Q., Baldé A., Catarino L. (2020). Present and Potential Uses of *Moringa oleifera* as a Multipurpose Plant in Guinea-Bissau. S. Afr. J. Bot..

[224] Bancessi A., Teodósio R., Duarte E., Baldé A., Catarino L., Nazareth T. (2022). Moringa as a Household Water Purification Method—Community Perception and Pilot Study in Guinea-Bissau. BMC Public. Health.

[225] Fernandes Â., Bancessi A., Pinela J., Dias M.I., Liberal Â., Calhelha R.C., Ćirić A., Soković M., Catarino L., Ferreira I.C.F.R. (2021). Nutritional and Phytochemical Profiles and Biological Activities of *Moringa oleifera* Lam. Edible Parts from Guinea-Bissau (West Africa). Food Chem..

[226] Fernandes Â., Liberal Â., Pinela J., Finimundy T.C., Bancessi A., Ćirić A., Soković M., Catarino L., Ferreira I.C.F.R., Barros L. (2021). Compositional Features and Biological Activities of Wild and Commercial *Moringa oleifera* Leaves from Guinea-Bissau. Food Biosci..

[227] Chang Y., Liu H., Liu M., Liao X., Sahu S.K., Fu Y., Song B., Cheng S., Kariba R., Muthemba S. (2019). The Draft Genomes of Five Agriculturally Important African Orphan Crops. Gigascience.

[228] Kamau L.N., Mbaabu M.P., Mbaria J.M., Karuri G.P., Kiama S.G. (2016). Knowledge and Demand for Medicinal Plants Used in the Treatment and Management of Diabetes in Nyeri County, Kenya. J. Ethnopharmacol..

[229] Kilingo F.M., Bernard Z., Hongbin C. (2022). Study of Domestic Wastewater Treatment Using *Moringa oleifera* Coagulant Coupled with Vertical Flow Constructed Wetland in Kibera Slum, Kenya. Environ. Sci. Pollut. Res..

[230] Muluvi G., Sprent J., Soranzo N., Provan J., Odee D., Folkard G., McNicol J., Powell W. (1999). Amplified Fragment Length Polymorphism (AFLP) Analysis of Genetic Variation in *Moringa oleifera* Lam. Mol. Ecol..

[231] Walter A., Samuel W., Peter A., Joseph O. (2011). Antibacterial Activity of *Moringa oleifera* and *Moringa stenopetala* Methanol and N-Hexane Seed Extracts on Bacteria Implicated in Water Borne Diseases. Afr. J. Microbiol. Res..

[232] Waterman C., Peterson A., Schelle C., Vosti S.A., McMullin S. (2021). Assessing the Economic Viability of Commercial Moringa Production for Kenyan Small-Scale Farmers. J. Agribus. Dev. Emerg. Econ..

[233] Awoyale W., Asiedu R., Kawalawu W.K.C., Maziya-Dixon B., Abass A., Edet M., Adetunji M.O. (2018). Assessment of Heavy Metals and Microbial Contamination of Gari from Liberia. Food Sci. Nutr..

[234] Mahklouf M.H. (2019). A New Record *Moringa oleifera* Lam. (Moringaceae) in the Flora of Libya. J. Wildl. Biodivers..

[235] Ramaroson Rakotosamimanana V., Valentin D., Arvisenet G. (2015). How to Use Local Resources to Fight Malnutrition in Madagascar? A Study Combining a Survey and a Consumer Test. Appetite.

[236] Rubio L.M. (2019). Acceptance and Knowledge of Moringa and Foliar Extract of Alfalfa in Different Centers of Toliara and Fianarantsoa in Madagascar. Nutr. Clin. Diet. Hosp..

[237] Syeda A.M., Riazunnisa K. (2020). Data on GC-MS Analysis, in Vitro Anti-Oxidant and Anti-Microbial Activity of the *Catharanthus roseus* and *Moringa oleifera* Leaf Extracts. Data Brief..

[238] McConnachie G.L., Folkard G.K., Mtawali M.A., Sutherland J.P. (1999). Field Trials of Appropriate Hydraulic Flocculation Processes. Water Res..

[239] Mwamatope B., Tembo D., Chikowe I., Kampira E., Nyirenda C. (2020). Total Phenolic Contents and Antioxidant Activity of *Senna singueana*, *Melia azedarach*, *Moringa oleifera* and *Lannea discolor* Herbal Plants. Sci. Afr..

[240] Mwamatope B., Tembo D., Chikowe I., Maliwichi-Nyirenda C., Kampira E., Masumbu F.F. (2022). Ethnobotanical Study of Medicinal Plants Used for Management of Cancer in Karonga District, Northern Malawi. Anticancer. Agents Med. Chem..

[241] Pritchard M., Craven T., Mkandawire T., Edmondson A.S., O’Neill J.G. (2010). A Comparison between *Moringa oleifera* and Chemical Coagulants in the Purification of Drinking Water—An Alternative Sustainable Solution for Developing Countries. Phys. Chem. Earth Parts A/B/C.

[242] Pritchard M., Craven T., Mkandawire T., Edmondson A.S., O’Neill J.G. (2010). A Study of the Parameters Affecting the Effectiveness of *Moringa oleifera* in Drinking Water Purification. Phys. Chem. Earth Parts A/B/C.

[243] Pritchard M., Mkandawire T., O’Neill J.G. (2009). Groundwater Pollution in Shallow Wells in Southern Malawi and a Potential Indigenous Method of Water Purification. Appropriate Technologies for Environmental Protection in the Developing World.

[244] Pritchard M., Mkandawire T., Edmondson A., O’Neill J.G., Kululanga G. (2009). Potential of Using Plant Extracts for Purification of Shallow Well Water in Malawi. Phys. Chem. Earth Parts A/B/C.

[245] Sagona W.C.J., Chirwa P.W., Sajidu S.M. (2020). The Miracle Mix of Moringa: Status of Moringa Research and Development in Malawi. S. Afr. J. Bot..

[246] Vunain E., Masoamphambe E.F., Mpeketula P.M.G., Monjerezi M., Etale A. (2019). Evaluation of Coagulating Efficiency and Water Borne Pathogens Reduction Capacity of *Moringa oleifera* Seed Powder for Treatment of Domestic Wastewater from Zomba, Malawi. J. Environ. Chem. Eng..

[247] Warhurst A.M., McConnachie G.L., Pollard S.J.T. (1996). The Production of Activated Carbon for Water Treatment in Malawi from the Waste Seed Husks of *Moringa oleifera*. Water Sci. Technol..

[248] Mahomoodally M.F., Ramalingum N. (2015). An Investigation into the Consumption Patterns, Attitude, and Perception of Mauritians towards Common Medicinal Food Plants. J. Herb. Med..

[249] Neergheen-Bhujun V.S., Ruhomally Z.B., Dunneram Y., Boojhawon R., Chan Sun M. (2020). Consumption Patterns, Determinants and Barriers of the Underutilised *Moringa oleifera* Lam in Mauritius. S. Afr. J. Bot..

[250] Cumbe T.A., Sessim A.G., López-González F.A., Zago D., Alforma A.M.P., Barcellos J.O.J. (2021). Bioeconomic Evaluation of Feedings Strategies in the Yearling Beef Cattle System in Mozambique. Livest. Sci..

[251] Marrufo T., Nazzaro F., Mancini E., Fratianni F., Coppola R., De Martino L., Agostinho A., De Feo V. (2013). Chemical Composition and Biological Activity of the Essential Oil from Leaves of *Moringa oleifera* Lam. Cultivated in Mozambique. Molecules.

[252] Abasse T.A., Gouzayé A., Woltering L., Pasternak D. (2007). The Role of Indigenous Leafy Vegetables on Daily Diet and Rural and Urban Economy of Niger. Acta Hortic..

[253] Bellostas N., Sorensen J.C., Nikiema A., Sorensen H., Pasternak D., Kumar S. (2010). Glucosinolates in Leaves of *Moringa* Species Grown and Disseminated in Niger. Afr. J. Agric. Res..

[254] Freiberger C.E., Vanderjagt D.J., Pastuszyn A., Glew R.S., Mounkaila G., Millson M., Glew R.H. (1998). Nutrient Content of the Edible Leaves of Seven Wild Plants from Niger. Plant Foods Hum. Nutr..

[255] Halilou M.S., Ba M.N., Karimoune L., Doumma A. (2022). Farmers’ Knowledge, Perceptions and Management of the *Moringa* Tree Defoliator, *Noorda blitealis* Walker (Lepidoptera: Crambidae), in Niger. Int. J. Trop. Insect Sci..

[256] Ratnadass A., Ryckewaert P., Claude Z., Nikiema A., Pasternak D., Woltering L., Thunes K., Zakari-Moussa O. (2011). New Ecological Options for the Management of Horticultural Crop Pests in Sudano-Sahelian Agroecosystems of West Africa. Acta Hortic..

[257] Sena L.P., VanderJagt D.J., Rivera C., Tsin A.T.C., Muhamadu I., Mahamadou O., Millson M., Pastuszyn A., Glew R.H. (1998). Analysis of Nutritional Components of Eight Famine Foods of the Republic of Niger. Plant Foods Hum. Nutr..

[258] Aba S.C., Baiyeri K.P., Ortiz R. (2021). Soil Fertility Dynamics and Yield Responses of Four Plantain Genotypes to Different Nutrient Management Systems in Nsukka, Southeastern Nigeria. Fruits.

[259] Abubakar I.B., Kankara S.S., Malami I., Danjuma J.B., Muhammad Y.Z., Yahaya H., Singh D., Usman U.J., Ukwuani-Kwaja A.N., Muhammad A. (2022). Traditional Medicinal Plants Used for Treating Emerging and Re-Emerging Viral Diseases in Northern Nigeria. Eur. J. Integr. Med..

[260] Abubakar S., Usman A.R., Isa N.F., Khandaker M.U., Abubakar N. (2015). Investigation of Therapeutic Potentials of Some Selected Medicinal Plants Using Neutron Activation Analysis. AIP Conf. Proc..

[261] Adeyemi S., Larayetan R., Onoja A.D., Ajayi A., Yahaya A., Ogunmola O.O., Adeyi A.O., Chijioke O. (2021). Anti-Hemorrhagic Activity of Ethanol Extract of *Moringa oleifera* Leaf on Envenomed Albino Rats. Sci. Afr..

[262] Adeyemo R. (2017). Moringa Sustainability: The Capital and Credit Available in a Developing Country. Acta Hortic..

[263] Aduro A., Ebenso B. (2019). Qualitative Exploration of Local Knowledge, Attitudes and Use of *Moringa oleifera* Seeds for Home-Based Water Purification and Diarrhoea Prevention in Niger State, Nigeria. J. Water Sanit. Hyg. Dev..

[264] Agbagwa O.E., Ekeke C., Israel P.C. (2022). Antibacterial Efficacy and Healing Potential of Honey from Different Zones in Nigeria on Diabetic-Induced Wound Infection in Wistar Rats. Int. J. Microbiol..

[265] Agbede J.O., Omotoso F.P., Oloruntola O.D., Ayeni O.A., Aletor V.A. (2019). A Preliminary Study of Fonio–Moringa Seed Meal-based Complementary Food in Wistar Rats. J. Food Biochem..

[266] Agbede T.M., Adekiya A.O., Ale M.O., Eifediyi E.K., Olatunji C.A. (2019). Effects of Green Manures and NPK Fertilizer on Soil Properties, Tomato Yield and Quality in the Forest-Savanna Ecology of Nigeria. Exp. Agric..

[267] Ajuogu P.K., Mgbere O.O., Bila D.S., McFarlane J.R. (2019). Hormonal Changes, Semen Quality and Variance in Reproductive Activity Outcomes of Post Pubertal Rabbits Fed *Moringa oleifera* Lam. Leaf Powder. J. Ethnopharmacol..

[268] Akanbi W.B., Asafa R.F., Ojo M.A. (2019). Effect of Growth Media Composition on Early Growth and Development of *Moringa* (*Moringa oleifera* L.) Seedlings. Pertanika J. Trop. Agric. Sci..

[269] Amaeze O., Eng H., Horlbogen L., Varma M.V.S., Slitt A. (2021). Cytochrome P450 Enzyme Inhibition and Herb-Drug Interaction Potential of Medicinal Plant Extracts Used for Management of Diabetes in Nigeria. Eur. J. Drug Metab. Pharmacokinet..

[270] Amaeze O., Marques E.S., Wei W., Lazzaro S., Johnson N., Varma M.V.S., Slitt A. (2021). Evaluation of Nigerian Medicinal Plants Extract on Human P-Glycoprotein and Cytochrome P450 Enzyme Induction: Implications for Herb-Drug Interaction. Curr. Drug Metab..

[271] Amaeze O.U., Aderemi-Williams R.I., Ayo-Vaughan M.A., Ogundemuren D.A., Ogunmola D.S., Anyika E.N. (2018). Herbal Medicine Use among Type 2 Diabetes Mellitus Patients in Nigeria: Understanding the Magnitude and Predictors of Use. Int. J. Clin. Pharm..

[272] Anele U.Y., Arigbede O.M., Olanite J.A., Adekunle I.O., Jolaosho A.O., Onifade O.S., Oni A.O. (2008). Early Growth and Seasonal Chemical Composition of Three Indigenous Multipurpose Tree Species (MPTS) in Abeokuta, Nigeria. Agrofor. Syst..

[273] Anjorin T.S., Ikokoh P., Okolo S. (2010). Mineral Composition of *Moringa oleifera* Leaves, Pods and Seeds from Two Regions in Abuja, Nigeria. Int. J. Agric. Biol..

[274] Sydney Aprioku J., Robinson O., Wolfe Obianime A., Tamuno I. (2022). *Moringa* Supplementation Improves Immunological Indices and Hematological Abnormalities in Seropositive Patients Receiving HAARTs. Afr. Health Sci..

[275] Arigbede O.M., Tan Z.L., Anele U.Y., Sun Z.H., Tang S.X., Han X.F., Zhou C.S., Zeng B. (2012). Effects of Age and Species on Agronomic Performance, Chemical Composition and in vitro Gas Production of Some Tropical Multi-Purpose Tree Species. J. Agric. Sci..

[276] Atawodi S.E., Atawodi J.C., Idakwo G.A., Pfundstein B., Haubner R., Wurtele G., Bartsch H., Owen R.W. (2010). Evaluation of the Polyphenol Content and Antioxidant Properties of Methanol Extracts of the Leaves, Stem, and Root Barks of *Moringa oleifera* Lam. J. Med. Food.

[277] Attah A.F., Moody J.O., Sonibare M.A., Salahdeen H.H., Akindele O.O., Nnamani P.O., Diyaolu O.A., Raji Y. (2020). Aqueous Extract of *Moringa oleifera* Leaf Used in Nigerian Ethnomedicine Alters Conception and Some Pregnancy Outcomes in Wistar Rat. S. Afr. J. Bot..

[278] Aviara N.A., Musa W.B., Owolarafe O.K., Ogunsina B.S., Oluwole F.A. (2015). Effect of Processing Conditions on Oil Point Pressure of *Moringa oleifera* Seed. J. Food Sci. Technol..

[279] Ayotunde E.O., Fagbenro O.A., Adebayo O.T. (2011). Histological Changes in Oreochromis Niloticus (Linnaeus 1779) Exposed to Aqueous Extract of *Moringa oleifera* Seeds Powder. Turk. J. Fish Aquat. Sci..

[280] Barminas J.T., Charles M., Emmanuel D. (1998). Mineral Composition of Non-Conventional Leafy Vegetables. Plant Foods Human. Nutr..

[281] Ebiloma G.U., Igoli J.O., Katsoulis E., Donachie A.-M., Eze A., Gray A.I., de Koning H.P. (2017). Bioassay-Guided Isolation of Active Principles from Nigerian Medicinal Plants Identifies New Trypanocides with Low Toxicity and No Cross-Resistance to Diamidines and Arsenicals. J. Ethnopharmacol..

[282] Etuk E.U., Mohammed B.J. (2009). Informant Consensus Selection Method: A Reliability Assessment on Medicinal Plants Used in North Western Nigeria for the Treatment of Diabetes Mellitus. Afr. J. Pharm. Pharmacol..

[283] Fagbenro J.A., Oshunsanya S.O., Oyeleye B.A. (2015). Effects of Gliricidia Biochar and Inorganic Fertilizer on Moringa Plant Grown in an Oxisol. Commun. Soil. Sci. Plant Anal..

[284] Gambo A., Gqaleni N., Babalola T.K. (2022). Dietary Diversity and Impact of *Moringa oleifera* Lam. Leaves Supplemented-Diet on the Nutritional Status and CD4 Cell Counts of Patients Receiving Antiretroviral Therapy in Nigeria: A Double-Blind Randomized Trial. Heliyon.

[285] Gambo A., Moodley I., Babashani M., Babalola T.K. (2021). Impact of *Moringa oleifera* Leaves Supplementation on Quality of Life of People Living with HIV: A Double-Blind Randomized Controlled Trial. Qual. Life Res..

[286] Gambo A., Moodley I., Babashani M., Babalola T.K., Gqaleni N. (2021). A Double-Blind, Randomized Controlled Trial to Examine the Effect of *Moringa oleifera* Leaf Powder Supplementation on the Immune Status and Anthropometric Parameters of Adult HIV Patients on Antiretroviral Therapy in a Resource-Limited Setting. PLoS ONE.

[287] Iwuji S.C., Ogbonna C.V., Iwu C.I., Okafor W.C., Chibuike E.C. (2021). Comparative Effects of Solvents on the Herbal Extraction of Antidiabetic Phytochemicals. J. Pharm. Res. Int..

[288] Jimoh W.A., Ayeloja A.A., Badmus G.O., Olateju K.O. (2020). Antibacterial and Antifungal Effect of Moringa (*Moringa oleifera*) Seedmeal on Marinated Smoked African Mud Catfish (*Clarias gariepinus*). J. Food Saf..

[289] Lawal U., Mohammed R.T., Gidado S.M., Kankara S.S. (2023). Ethnobotanical Survey and Nutritional Composition of Medicinal Plants Used for Traditional Galactagogue Practice in Katsina State, Nigeria. Adv. Tradit. Med..

[290] Lockett C.T., Calvert C.C., Grivetti L.E. (2000). Energy and Micronutrient Composition of Dietary and Medicinal Wild Plants Consumed during Drought. Study of Rural Fulani, Northeastern Nigeria. Int. J. Food Sci. Nutr..

[291] Mahdy T., Giorgi M., Adewole T., Ernest F., Idoko I., Matey M., Ozele N., Oladipo S., Sunday M. (2012). Effect of *Moringa oleifera*, Activated Carbon and Wood Charcoal on Biochemical and Hematological Parameters of Wistar Rats Exposed to Lead Acetate. Med. Weter. Vet. Med. Sci. Pract..

[292] Manuwa S.I., Sedara A.M., Tola F.A. (2020). Design, Fabrication and Performance Evaluation of *Moringa (oleifera)* Dried Leaves Pulverizer. J. Agric. Food Res..

[293] Namla D., Mohammed B.S., Ibrahim I.M., Dane S. (2022). Antimicrobial Activities of *Moringa oleifera* and *Psidium guajava* against Bacterial and Fungal Strains. J. Res. Med. Dent. Sci..

[294] Obayelu O.A., Adeoti A.I., Akinlade A.A. (2015). Consumers’ Willingness to Pay for Labelled and Certified *Moringa* Products in Ogun State, Nigeria. Int. Food Res. J..

[295] Ogunbinu A.O., Flamini G., Cioni P.L., Adebayo M.A., Ogunwande I.A. (2009). Constituents of *Cajanus cajan* (L.) Millsp., *Moringa oleifera* Lam., *Heliotropium indicum* L. and *Bidens pilosa* L. from Nigeria. Nat. Prod. Commun..

[296] Ogundiran M.B., Mekwunyei N.S., Adejumo S.A. (2018). Compost and Biochar Assisted Phytoremediation Potentials of *Moringa oleifera* for Remediation of Lead Contaminated Soil. J. Environ. Chem. Eng..

[297] Ojo T.O., Adetoro A.A., Ogundeji A.A., Belle J.A., Ngidi M.S.C. (2022). Unlocking the Commercialization Potentials of *Moringa oleifera* Production in Southwestern Nigeria. Afr. J. Sci. Technol. Innov. Dev..

[298] Okoya A.A., Olaiya O.O., Akinyele A.B., Ochor N.O. (2020). Efficacy of *Moringa oleifera* Seed Husk as Adsorptive Agent for Trihalomethanes from a Water Treatment Plant in Southwestern, Nigeria. J. Chem..

[299] Oladeji O.S., Odelade K.A., Oloke J.K. (2020). Phytochemical Screening and Antimicrobial Investigation of *Moringa oleifera* Leaf Extracts. Afr. J. Sci. Technol. Innov. Dev..

[300] Oladeji O.S., Oluyori A.P., Bankole D.T., Afolabi T.Y. (2020). Natural Products as Sources of Antimalarial Drugs: Ethnobotanical and Ethnopharmacological Studies. Scientifica.

[301] Olaoye A.B., Ologunde C.A., Molehin O.R., Nwankwo I. (2021). Comparative Antioxidant Analysis of *Moringa oleifera* Leaf Extracts from South Western States in Nigeria. Futur. J. Pharm. Sci..

[302] Oyekale A.S. (2012). Nutritional Outlooks of *Moringa oleifera* and African Malnutrition Challenges: A Case Study of Nigeria. Life Sci. J. Acta Zhengzhou Univ. Overseas Ed..

[303] Popoola J.O., Obembe O.O. (2013). Local Knowledge, Use Pattern and Geographical Distribution of *Moringa oleifera* Lam. (Moringaceae) in Nigeria. J. Ethnopharmacol..

[304] Stevens C.G., Ugese F.D., Baiyeri P.K. (2021). Variations in Mineral and Vitamin Content of *Moringa oleifera* Provenances across Nigeria. For. Trees Livelihoods.

[305] Taiwo A.S., Adenike K., Aderonke O. (2020). Efficacy of a Natural Coagulant Protein from *Moringa oleifera* (Lam) Seeds in Treatment of Opa Reservoir Water, Ile-Ife, Nigeria. Heliyon.

[306] Titiloye J.O., Abu Bakar M.S., Odetoye T.E. (2013). Thermochemical Characterisation of Agricultural Wastes from West Africa. Ind. Crops Prod..

[307] Ugese F.D., Iordye N. (2021). Moringa and Sesame Seed Oil Coatings Enhanced Sensory Attributes of Stored Sweet Orange Fruits. Acta Hortic..

[308] Dièye A.M., Sarr A., Diop S.N., Ndiaye M., Sy G.Y., Diarra M., Rajraji/Gaffary I., Ndiaye/Sy A., Faye B. (2008). Medicinal Plants and the Treatment of Diabetes in Senegal: Survey with Patients. Fundam. Clin. Pharmacol..

[309] Diouf M., Gueye M., Faye B., Dieme O., Lo C. (2007). The Commodity Systems of Four Indigenous Leafy Vegetables in Senegal. Water SA.

[310] Faye B., Bucheton B., Banuls A.L., Senghor M.W., Niang A.A., Diedhiou S., Konate O., Dione M.M., Hide M., Mellul S. (2011). Seroprevalence of *Leishmania infantum* in a Rural Area of Senegal: Analysis of Risk Factors Involved in Transmission to Humans. Trans. R. Soc. Trop. Med. Hyg..

[311] Gold M., Dayer P., Faye M.C.A.S., Clair G., Seck A., Niang S., Morgenroth E., Strande L. (2016). Locally Produced Natural Conditioners for Dewatering of Faecal Sludge. Environ. Technol..

[312] Mathieu G., Meissa D. (2007). Traditional Leafy Vegetables in Senegal: Diversity and Medicinal Uses. Afr. J. Tradit. Complement. Altern. Med..

[313] Zeitoun H., Michael-Jubeli R., El Khoury R., Baillet-Guffroy A., Tfayli A., Salameh D., Lteif R. (2020). Skin Lightening Effect of Natural Extracts Coming from Senegal Botanical Biodiversity. Int. J. Dermatol..

[314] James P.B., Kamara H., Bah A.J., Steel A., Wardle J. (2018). Herbal Medicine Use among Hypertensive Patients Attending Public and Private Health Facilities in Freetown Sierra Leone. Complement. Ther. Clin. Pract..

[315] Ranasinghe S., Ansumana R., Lamin J.M., Bockarie A.S., Bangura U., Buanie J.A.G., Stenger D.A., Jacobsen K.H. (2015). Herbs and Herbal Combinations Used to Treat Suspected Malaria in Bo, Sierra Leone. J. Ethnopharmacol..

[316] Adetola O.Y., Kruger J., White Z., Taylor J.R.N. (2019). Comparison between Food-to-Food Fortification of Pearl Millet Porridge with Moringa Leaves and Baobab Fruit and with Adding Ascorbic and Citric Acid on Iron, Zinc and Other Mineral Bioaccessibility. LWT.

[317] Adhikari S., Siebert S.J., Jordaan A. (2021). Chromium Dust Deposition on *Moringa oleifera* Leaves Harvested by Local Communities in Sekhukhuneland, South Africa. Acta Hortic..

[318] Adhikari S., Struwig M., Siebert S.J. (2022). Identifying Common Trees and Herbaceous Plants to Mitigate Particulate Matter Pollution in a Semi-Arid Mining Region of South Africa. Climate.

[319] Bassey K., Mabowe M., Mothibe M., Witika B.A. (2022). Chemical Characterization and Nutritional Markers of South African *Moringa oleifera* Seed Oils. Molecules.

[320] Bopape-Mabapa M.P., Ayisi K.K., Mariga I.K., Mashao F.M. (2021). Moringa (*Moringa oleifera*) Leaf Nutritional Composition as Influenced by Soil Physical and Chemical Properties and Tree Age under Diverse Agro-Ecological Conditions. Appl. Ecol. Environ. Res..

[321] Chitiyo S.T., Ncube B., Ndhlala A.R., Tsvuura Z. (2021). Biochemical Responses of *Moringa oleifera* Lam. Plants to Graded Moisture Deficit. S. Afr. J. Bot..

[322] du Toit E.S., Fotouo H., Robbertse P.J. (2017). Seed Storage Conditions Influence Germination of *Moringa oleifera* Lam. Seed. Acta Hortic..

[323] du Toit E.S., Sithole J., Vorster J. (2020). Leaf Harvesting Severity Affects Total Phenolic and Tannin Content of Fresh and Dry Leaves of *Moringa oleifera* Lam. Trees Growing in Gauteng, South Africa. S. Afr. J. Bot..

[324] du Toit E.S., Sithole J., Vorster J. (2020). Pruning Intensity Influences Growth, Flower and Fruit Development of *Moringa oleifera* Lam. under Sub-Optimal Growing Conditions in Gauteng, South Africa. S. Afr. J. Bot..

[325] Govender K., Naicker A. (2020). Product Development, Nutrient Analysis and Sensory Evaluation of Maize Chips, Enhanced with *Moringa oleifera*. J. Consum. Sci..

[326] Hedhili A., Akinyemi B.E., Otunola G.A., Husson F., Valentin D. (2022). Food Habits and Beliefs about *Moringa oleifera* among South African Student Mothers: A Qualitative Study. Cah. Agric..

[327] Ibrahim T.A., Hassen A., Apostolides Z. (2022). The Antimethanogenic Potentials of Plant Extracts: Their Yields and Phytochemical Compositions as Affected by Extractive Solvents. Plants.

[328] Kivevele T., Huan Z. (2015). Influence of Metal Contaminants and Antioxidant Additives on Storage Stability of Biodiesel Produced from Non-Edible Oils of Eastern Africa Origin (*Croton megalocarpus* and *Moringa oleifera* Oils). Fuel.

[329] Lubaale J., Taylor J.R.N., Emmambux M.N., Duodu K.G. (2023). Extrusion Cooking of Food-to-food Fortified Wholegrain Sorghum-based Porridges Enhances Caco-2 Ferritin Formation. Cereal Chem..

[330] Mabapa P.M., Ayisi K.K., Mariga I.K. (2018). Comparison of Gas Exchange in *Moringa oleifera* and Other Drought Tolerant Tree Species for Climate Change Mitigation under Semi-Arid Condition of Northern South Africa. Int. J. Agric. Biol..

[331] Maiyo C.F., Moodley R., Singh M. (2016). Cytotoxicity, Antioxidant and Apoptosis Studies of Quercetin-3-O Glucoside and 4-(β-D-Glucopyranosyl-1→4-α-L-Rhamnopyranosyloxy)-Benzyl Isothiocyanate from *Moringa oleifera*. Anticancer. Agents Med. Chem..

[332] Managa L.R., du Toit E.S., Prinsloo G. (2021). Variations in the Leaf Metabolite Profile between Hydroponic and Field Grown *Moringa oleifera* Lam. Genotypes. Biochem. Syst. Ecol..

[333] Manduwa D.M., du Toit E.S., Robbertse P.J. (2018). Study on the Effect of Temperature and Flower Age on Pollen Performance, Stigma Receptivity and Fruit-Set of *Moringa oleifera* Lam. Acta Hortic..

[334] Masete F.M., Munjonji L., Ayisi K.K., Mopape-Mabapa M.P. (2022). Cowpea Growth and Nitrogen Fixation Performance under Different Mulch Treatments. Agriculture.

[335] Mhlomi Y.N., Oshiomame Unuofin J., Otunola G.A., Afolayan A.J. (2022). Assessment of Rats Fed Protein-Deficient Diets Supplemented with *Moringa oleifera* Leaf Meal. Curr. Res. Nutr. Food Sci. J..

[336] Modisaojang-Mojanaga M.M.C., Ogbuewu I.P., Mokolopi B.G., Oguttu J.W., Mbajiorgu C.A. (2019). Mineral Composition of *Moringa oleifera* Leaf Meal (Molm) and the Responses of Ross 308 Broilers to Molm Supplementation. Appl. Ecol. Environ. Res..

[337] Moichela F.T., Adefolaju G.A., Henkel R.R., Opuwari C.S. (2021). Aqueous Leaf Extract of *Moringa oleifera* Reduced Intracellular ROS Production, DNA Fragmentation and Acrosome Reaction in Human Spermatozoa in vitro. Andrologia.

[338] Mokgehle S., Araya N., Mofokeng M., Makgato M., Amoo S., Maboka K., du Plooy C., Araya H. (2022). Regrowth Response and Nutritional Composition of *Moringa oleifera* to Cutting Back in Three Agro-Ecological Zones in South Africa. Horticulturae.

[339] Motis T.N., Longfellow J.M., Jani A.D., Lingbeek B.J., D’Aiuto C.J., Bergen J.C.J. (2017). Productivity of *Moringa oleifera* Augmented with Intercropped Tropical Legumes. Acta Hortic..

[340] Moyo B., Masika P.J., Muchenje V. (2012). Effect of Supplementing Crossbred Xhosa Lop-Eared Goat Castrates with *Moringa oleifera* Leaves on Growth Performance, Carcass and Non-Carcass Characteristics. Trop. Anim. Health Prod..

[341] Mudau T.E., Olowoyo J.O., Amoo S.O. (2022). Ethnobotanical Assessment of Medicinal Plants Used Traditionally for Treating Diabetes in Vhembe District, Limpopo Province, South Africa. S. Afr. J. Bot..

[342] Muhl Q.E., Kanfer F.H.J., du Toit E.S., Steyn J.M. (2016). Quantification of Intracellular Seed Compounds of *Moringa oleifera* Lam. Using Digital Image Analysis. Acta Hortic..

[343] Naidoo P. (2023). Greener on the Other Side: Tracing Stories of Amaranth and *Moringa* through Indenture. Agenda.

[344] Ndhlala A., Mulaudzi R., Ncube B., Abdelgadir H., du Plooy C., Van Staden J. (2014). Antioxidant, Antimicrobial and Phytochemical Variations in Thirteen *Moringa oleifera* Lam. Cultivars. Molecules.

[345] Netshiheni K.R., Mashau M.E., Jideani A.I.O. (2019). Nutritional and Sensory Properties of Instant Maize Porridge Fortified with *Moringa oleifera* Leaves and Termite (*Macrotermes falciger*) Powders. Nutr. Food Sci..

[346] Ngcobo B.L., Bertling I. (2021). Influence of Foliar *Moringa oleifera* Leaf Extract (MLE) Application on Growth, Fruit Yield and Nutritional Quality of Cherry Tomato. Acta Hortic..

[347] Ntila S.L., Ndhlala A.R., Mashela P.W., Kolanisi U., Siwela M. (2020). Supplementation of a Complementary White Maize Soft Porridge with *Moringa oleifera* Powder as a Promising Strategy to Increase Nutritional and Phytochemical Values: A Research Note. S. Afr. J. Bot..

[348] Ntila S., Ndhlala A.R., Kolanisi U., Abdelgadir H., Siwela M. (2019). Acceptability of a Moringa-Added Complementary Soft Porridge to Caregivers in Hammanskraal, Gauteng Province and Lebowakgomo, Limpopo Province, South Africa. South Afr. J. Clin. Nutr..

[349] Olusanya R.N., Kolanisi U., van Onselen A., Ngobese N.Z., Siwela M. (2020). Nutritional Composition and Consumer Acceptability of *Moringa oleifera* Leaf Powder (MOLP)-Supplemented Mahewu. S. Afr. J. Bot..

[350] Omodanisi E., Aboua Y., Oguntibeju O. (2017). Assessment of the Anti-Hyperglycaemic, Anti-Inflammatory and Antioxidant Activities of the Methanol Extract of *Moringa oleifera* in Diabetes-Induced Nephrotoxic Male Wistar Rats. Molecules.

[351] Pakade V., Cukrowska E., Chimuka L. (2013). Comparison of Antioxidant Activity of *Moringa oleifera* and Selected Vegetables in South Africa. S. Afr. J. Sci..

[352] Pakade V., Cukrowska E., Chimuka L. (2013). Metal and Flavonol Contents of *Moringa oleifera* Grown in South Africa. S. Afr. J. Sci..

[353] Ralepele F.M., Chimuka L., Nuapia Y., Risenga I. (2021). UPLC-DAD-QTOF-MS/MS Analysis of Targeted Poly-Phenolic Compounds from *Moringa oleifera* Leaves as Function of Seasonal Responses. S. Afr. J. Bot..

[354] Rapatsa M., Moyo N. (2014). Effect of *Moringa oleifera* on the Histology, Haematology and Growth of *Oreochromis mossambicus* in Aquadams^®^ Fertilised with Chicken Manure in South Africa. Afr. J. Aquat. Sci..

[355] Ratshilivha N., Du Toit E.S., Vahrmeijer J.T., Eloff J.N. (2017). Yield and Quality Responses of *Moringa oleifera* Lam. to Nitrogen Fertilization. Acta Hortic..

[356] Ratshilivha N., Eloff K., du Toit E.S. (2021). Vegetative Propagation of *Moringa oleifera* Trees Growing in Pretoria, South Africa, with Offsets Producing Equally Consistent Bioactivity. Acta Hortic..

[357] Rikhotso M.M., Magwaza L.S., Tesfay S.Z., Mditshwa A. (2019). Evaluating the Efficacy of Chitosan and CMC Incorporated with *Moringa* Leaf Extracts on Reducing Peteca Spot Incidence on ‘Eureka’ Lemon. J. Food Sci. Technol..

[358] Rikhotso M.M., Magwaza L.S., Tesfay S.Z., Mditshwa A. (2021). The Effect of Chitosan and CMC-Moringa Leaf Extracts on Peteca Spot of ‘Eureka’ Lemon. Acta Hortic..

[359] Sebola N.A., Mlambo V., Mokoboki H.K., Muchenje V. (2015). Growth Performance and Carcass Characteristics of Three Chicken Strains in Response to Incremental Levels of Dietary *Moringa oleifera* Leaf Meal. Livest. Sci..

[360] Semenya S.S., Maroyi A. (2018). Ethnobotanical Study of Plants Used Medicinally by Bapedi Traditional Healers to Treat Sinusitis and Related Symptoms in the Limpopo Province, South Africa. J. Appl. Bot. Food Qual..

[361] Semenya S.S., Maroyi A. (2018). Exotic Plants Used Therapeutically by Bapedi Traditional Healers for Respiratory Infections and Related Symptoms in the Limpopo Province, South Africa. Indian J. Tradit. Knowl..

[362] Semenya S., Potgieter M., Erasmus L. (2012). Ethnobotanical Survey of Medicinal Plants Used by Bapedi Healers to Treat Diabetes Mellitus in the Limpopo Province, South Africa. J. Ethnopharmacol..

[363] Sharaf-Eldin M.A., Alam P., Mahfouz S.A. (2017). Efficacy of Hard Seed Shell and Wings on Seed Germination of *Moringa oleifera*. Acta Hortic..

[364] Swanepoel K.M. (2021). Moringa as Option in Intercropping and Complementary Crop to Existing Agricultural Activities in South Africa. Acta Hortic..

[365] Tiloke C., Phulukdaree A., Chuturgoon A.A. (2013). The Antiproliferative Effect of *Moringa oleifera* Crude Aqueous Leaf Extract on Cancerous Human Alveolar Epithelial Cells. BMC Complement. Altern. Med..

[366] Tiloke C., Phulukdaree A., Chuturgoon A.A. (2016). The Antiproliferative Effect of *Moringa oleifera* Crude Aqueous Leaf Extract on Human Esophageal Cancer Cells. J. Med. Food.

[367] Tshabalala T., Ncube B., Moyo H.P., Abdel-Rahman E.M., Mutanga O., Ndhlala A.R. (2020). Predicting the Spatial Suitability Distribution of *Moringa oleifera* Cultivation Using Analytical Hierarchical Process Modelling. S. Afr. J. Bot..

[368] Tshabalala T., Abdel-Rahman E.M., Ncube B., Ndhlala A.R., Mutanga O. (2021). Leveraging of Hyperspectral Remote Sensing on Estimating Biomass Yield of *Moringa oleifera* Lam. Medicinal Plant. S. Afr. J. Bot..

[369] Zeru A.E., Hassen A., Apostolides Z., Tjelele J. (2022). Relationships between Agronomic Traits of Moringa Accessions and In Vitro Gas Production Characteristics of a Test Feed Incubated with or without *Moringa* Plant Leaf Extracts. Plants.

[370] Zungu N., van Onselen A., Kolanisi U., Siwela M. (2020). Assessing the Nutritional Composition and Consumer Acceptability of *Moringa oleifera* Leaf Powder (MOLP)-Based Snacks for Improving Food and Nutrition Security of Children. S. Afr. J. Bot..

[371] Ali H., König G.M., Khalid S.A., Wright A.D., Kaminsky R. (2002). Evaluation of Selected Sudanese Medicinal Plants for Their in Vitro Activity against Hemoflagellates, Selected Bacteria, HIV-1-RT and Tyrosine Kinase Inhibitory, and for Cytotoxicity. J. Ethnopharmacol..

[372] Ezaldeen E.A., Fahal A.H., Osman A. (2013). Mycetoma Herbal Treatment: The Mycetoma Research Centre, Sudan Experience. PLoS Negl. Trop. Dis..

[373] Alphonce S., Kaale L.D., Rweyemamu L.M.P., Millinga F. (2019). Proximate Composition of Fermented Cassava Meal “Mchuchume” Fortified with Soya Bean Flour and *Moringa oleifera* Leaves Powder. J. Food Sci. Technol..

[374] Alphonce S., Kaale L.D., Millinga F., Rweyemamu L.M. (2021). Enrichment of Fermented Cassava Meal ‘Mchuchume’ with Micronutrient Ingredients from Soya Bean Flour and *Moringa oleifera* Leaves Powder. J. Sci. Food Agric..

[375] Bisanz J.E., Enos M.K., PrayGod G., Seney S., Macklaim J.M., Chilton S., Willner D., Knight R., Fusch C., Fusch G. (2015). Microbiota at Multiple Body Sites during Pregnancy in a Rural Tanzanian Population and Effects of *Moringa*-Supplemented Probiotic Yogurt. Appl. Environ. Microbiol..

[376] Hekmat S., Morgan K., Soltani M., Gough R. (2015). Sensory Evaluation of Locally-Grown Fruit Purees and Inulin Fibre on Probiotic Yogurt in Mwanza, Tanzania and the Microbial Analysis of Probiotic Yogurt Fortified with *Moringa oleifera*. J. Health Popul. Nutr..

[377] Kibazohi O., Sangwan R.S. (2011). Vegetable Oil Production Potential from *Jatropha curcas*, *Croton megalocarpus*, *Aleurites moluccana*, *Moringa oleifera* and *Pachira glabra*: Assessment of Renewable Energy Resources for Bio-Energy Production in Africa. Biomass Bioenergy.

[378] Lunyera J., Wang D., Maro V., Karia F., Boyd D., Omolo J., Patel U.D., Stanifer J.W. (2016). Traditional Medicine Practices among Community Members with Diabetes Mellitus in Northern Tanzania: An Ethnomedical Survey. BMC Complement. Altern. Med..

[379] Marobhe N.J., Sabai S.M. (2021). Treatment of Drinking Water for Rural Households Using Moringa Seed and Solar Disinfection. J. Water Sanit. Hyg. Dev..

[380] Mmanda F.P., Lindberg J.E., Norman Haldén A., Mtolera M.S.P., Kitula R., Lundh T. (2020). Digestibility of Local Feed Ingredients in Tilapia *Oreochromis niloticus* Juveniles, Determined on Faeces Collected by Siphoning or Stripping. Fishes.

[381] Munyanziza E., Sarwatt S.V. (2003). Evaluation of *Moringa oleifera* for Food Security and Environmental Rehabilitation in Tanzanian Rural Areas. J. Trop. For. Sci..

[382] Ndemanisho E.E., Kimoro B.N., Mtengeti E.J., Muhikambele V.R.M. (2006). The Potential of Albizia Lebbeck as a Supplementary Feed for Goats in Tanzania. Agrofor. Syst..

[383] Sarwatt S.V., Kapange S.S., Kakengi A.M.V. (2002). Substituting Sunflower Seed-Cake with *Moringa oleifera* Leaves as a Supplemental Goat Feed in Tanzania. Agrofor. Syst..

[384] Shija A.E., Rumisha S.F., Oriyo N.M., Kilima S.P., Massaga J.J. (2019). Effect of *Moringa oleifera* Leaf Powder Supplementation on Reducing Anemia in Children below Two Years in Kisarawe District, Tanzania. Food Sci. Nutr..

[385] Abotsi K.E., Fare Y., Auzanneau F., Villon F., Métro N., Mawussi G., Kokou K. (2017). Croissance et Productivité Du *Moringa oleifera* Lam. En Plantation Agroforestière Au Togo. Acta Hortic..

[386] Fare Y., Métro N., Villon F., Auzanneau F. (2017). Vers Un Nouveau Modèle de Partenariat Multi-Acteur Dans l’entreprise Sociale et Solidaire Du Moringa Au Togo (Afrique de l’Ouest). Acta Hortic..

[387] Bennour N., Mighri H., Eljani H., Zammouri T., Akrout A. (2020). Effect of Solvent Evaporation Method on Phenolic Compounds and the Antioxidant Activity of *Moringa oleifera* Cultivated in Southern Tunisia. S. Afr. J. Bot..

[388] Bennour N., Mighri H., Bouhamda T., Mabrouk M., Apohan E., Yesilada O., Küçükbay H., Akrout A. (2021). *Moringa oleifera* Leaves: Could Solvent and Extraction Method Affect Phenolic Composition and Bioactivities?. Prep. Biochem. Biotechnol..

[389] Boumenjel A., Papadopoulos A., Ammari Y. (2021). Growth Response of *Moringa oleifera* (Lam) to Water Stress and to Arid Bioclimatic Conditions. Agrofor. Syst..

[390] Gharsallah K., Rezig L., Msaada K., Chalh A., Soltani T. (2021). Chemical Composition and Profile Characterization of *Moringa oleifera* Seed Oil. S. Afr. J. Bot..

[391] Marzougui N., Guasmi F., Dhouioui S., Bouhlel M., Hachicha M., Berndtsson R., Sleimi N. (2021). Efficiency of Different *Moringa oleifera* (Lam.) Varieties as Natural Coagulants for Urban Wastewater Treatment. Sustainability.

[392] Marzougui N., Hammami A., Guasmi F., Rejeb S. (2021). Effects of Temperature and Treated Urban Wastewater on Seed Germination and Seedling Growth in Different Populations of *Moringa oleifera* (Lam.). Indian J. Exp. Biol..

[393] Jilcott S.B., Ickes S.B., Ammerman A.S., Myhre J.A. (2010). Iterative Design, Implementation and Evaluation of a Supplemental Feeding Program for Underweight Children Ages 6–59 Months in Western Uganda. Matern. Child. Health J..

[394] Kaggwa B., Kyeyune H., Munanura E.I., Anywar G., Lutoti S., Aber J., Bagoloire L.K., Weisheit A., Tolo C.U., Kamba P.F. (2022). Safety and Efficacy of Medicinal Plants Used to Manufacture Herbal Products with Regulatory Approval in Uganda: A Cross-Sectional Study. Evid. Based Complement. Altern. Med..

[395] Kasolo J.N., Bimenya G.S., Ojok L., Ochieng J., Ogwal-Okeng J.W. (2010). Phytochemicals and Uses of *Moringa oleifera* Leaves in Ugandan Rural Communities. J. Med. Plants Res..

[396] Choga T., Ngadze E., Rugare J.T., Mabasa S., Makaza W., Gwatidzo V.O., Chikuta S., Karubanga G. (2021). Effect of Botanical Extracts on Late Blight (*Phytopthora infestans*) and Productivity of Tomato (*Solanum esculentum*). Int. J. Agron..

[397] Jambwa P., Katsande S., Matope G., McGaw L.J. (2022). Ethnoveterinary Remedies Used in Avian Complementary Medicine in Selected Communal Areas in Zimbabwe. Planta Med..

[398] Maroyi A. (2013). Use of Weeds as Traditional Vegetables in Shurugwi District, Zimbabwe. J. Ethnobiol. Ethnomed..

[399] Smith L.E., Chagwena D.T., Bourke C., Robertson R., Fernando S., Tavengwa N.V., Cairns J., Ndhlela T., Matumbu E., Brown T. (2022). Child Health, Agriculture and Integrated Nutrition (CHAIN): Protocol for a Randomised Controlled Trial of Improved Infant and Young Child Feeding in Rural Zimbabwe. BMJ Open.

[400] Kumssa D.B., Joy E.J., Young S.D., Odee D.W., Ander E.L., Broadley M.R. (2017). Variation in the Mineral Element Concentration of *Moringa oleifera* Lam. and *M. stenopetala* (Bak. f.) Cuf.: Role in Human Nutrition. PLoS ONE.

[401] Chadha M.L., Olouch M. (2007). Healthy Diet Gardening Kit—For Better Health and Income. Acta Hortic..

[402] Chadha M.L., Oluoch M.O., Silue D. (2007). Promoting Indigenous Vegetables for Health, Food Security, and Income Generation in Africa. Acta Hortic..

[403] Ondiba R.N., Ogello E.O., Kembenya E., Gichana Z., Obiero K. (2022). Future Demand and Supply of Aquafeed Ingredients: Outlines to Commercialize Non-Conventional Protein Ingredients to Enhance Aquaculture Production for Food Security in Sub-Saharan Africa. Aquat. Ecosyst. Health Manag..

[404] Yamato M., Ikeda S., Iwase K. (2009). Community of Arbuscular Mycorrhizal Fungi in Drought-Resistant Plants, *Moringa* spp., in Semiarid Regions in Madagascar and Uganda. Mycoscience.

[405] Ebert A.W., Palada M.C. (2017). Moringa—A Vegetable Tree for Improved Nutrition, Health and Income of Smallholder Farmers. Acta Hortic..

[406] Meale S.J., Chaves A.V., Baah J., McAllister T.A. (2011). Methane Production of Different Forages in In Vitro Ruminal Fermentation. Asian-Australas. J. Anim. Sci..

[407] Palada M.C., Crossman S.M., O’Donnell J.J., Onokpise O., Rockwood D., Worthen D., Willis T. (2002). Integrating High Value Horticultural Crops into Agroforestry Systems in the Tropics with Focus on Alley Cropping. Proceedings Symposium on Celebrating Minority Professionals in Forestry and Natural Resources Conservation.

